# The Genome Sequence of the Rumen Methanogen *Methanobrevibacter ruminantium* Reveals New Possibilities for Controlling Ruminant Methane Emissions

**DOI:** 10.1371/journal.pone.0008926

**Published:** 2010-01-28

**Authors:** Sinead C. Leahy, William J. Kelly, Eric Altermann, Ron S. Ronimus, Carl J. Yeoman, Diana M. Pacheco, Dong Li, Zhanhao Kong, Sharla McTavish, Carrie Sang, Suzanne C. Lambie, Peter H. Janssen, Debjit Dey, Graeme T. Attwood

**Affiliations:** Rumen Microbial Genomics, Food Metabolism and Microbiology Section, Food and Textiles Group, AgResearch Limited, Grasslands Research Centre, Palmerston North, New Zealand; University of Hyderabad, India

## Abstract

**Background:**

Methane (CH_4_) is a potent greenhouse gas (GHG), having a global warming potential 21 times that of carbon dioxide (CO_2_). Methane emissions from agriculture represent around 40% of the emissions produced by human-related activities, the single largest source being enteric fermentation, mainly in ruminant livestock. Technologies to reduce these emissions are lacking. Ruminant methane is formed by the action of methanogenic archaea typified by *Methanobrevibacter ruminantium*, which is present in ruminants fed a wide variety of diets worldwide. To gain more insight into the lifestyle of a rumen methanogen, and to identify genes and proteins that can be targeted to reduce methane production, we have sequenced the 2.93 Mb genome of *M. ruminantium* M1, the first rumen methanogen genome to be completed.

**Methodology/Principal Findings:**

The M1 genome was sequenced, annotated and subjected to comparative genomic and metabolic pathway analyses. Conserved and methanogen-specific gene sets suitable as targets for vaccine development or chemogenomic-based inhibition of rumen methanogens were identified. The feasibility of using a synthetic peptide-directed vaccinology approach to target epitopes of methanogen surface proteins was demonstrated. A prophage genome was described and its lytic enzyme, endoisopeptidase PeiR, was shown to lyse M1 cells in pure culture. A predicted stimulation of M1 growth by alcohols was demonstrated and microarray analyses indicated up-regulation of methanogenesis genes during co-culture with a hydrogen (H_2_) producing rumen bacterium. We also report the discovery of non-ribosomal peptide synthetases in *M. ruminantium* M1, the first reported in archaeal species.

**Conclusions/Significance:**

The M1 genome sequence provides new insights into the lifestyle and cellular processes of this important rumen methanogen. It also defines vaccine and chemogenomic targets for broad inhibition of rumen methanogens and represents a significant contribution to worldwide efforts to mitigate ruminant methane emissions and reduce production of anthropogenic greenhouse gases.

## Introduction

Global surface temperatures are predicted to increase between 1°C to 6°C during the twenty-first century, primarily due to increased levels of greenhouse gases (GHGs) in the atmosphere [1]. Methane (CH_4_) is a particularly potent GHG, having a global warming potential 21 times that of carbon dioxide (CO_2_) [1], and accounts for 16% of total global GHG emissions [2]. CH_4_ emissions from agriculture represent around 40% of the emissions produced by human-related activities, the single largest source being enteric fermentation in livestock, mainly from ruminant animals [3]. The worldwide demand for meat and milk is predicted to double by 2050 [4] and ruminant-based agriculture is expected to continue to be an important contributor to global CH_4_ emissions. Therefore, reducing CH_4_ emissions from ruminants will be important in meeting international commitments under the Kyoto Protocol [5] and also in ensuring the long-term sustainability of ruminant-based agriculture. Furthermore, as CH_4_ production in the rumen accounts for 2–12% of the ingested energy [6], it is predicted that reducing CH_4_ emissions from ruminants will also make more energy available to the animal and therefore increase productivity. Ruminant animals are particularly important to agriculture in New Zealand (NZ), producing a third of NZ's commodity exports [7] and accounting for a large proportion of internationally traded lamb and milk products [8]. The large number of ruminant animals farmed relative to the small human population gives NZ an unusual GHG emission profile, with ruminant CH_4_ emissions accounting for 31% of NZ's total GHGs [9].

Methane is formed in the ruminant fore-stomach (rumen) by methanogens, a subgroup of the Archaea. During normal rumen function, plant material is broken down by fibre-degrading microorganisms and fermented mainly to volatile fatty acids (VFAs), ammonia, hydrogen (H_2_) and CO_2_. Rumen methanogens principally use H_2_ to reduce CO_2_ to CH_4_ in a series of reactions that are coupled to ATP synthesis. The rumen harbours a variety of different methanogen species, but analyses of archaeal small subunit ribosomal RNA genes from rumen samples of ruminants on differing diets around the world suggest the majority fall into three main groups: *Methanobrevibacter*, *Methanomicrobium*, and a large, as-yet uncultured, group of rumen archaea referred to as rumen cluster C [10]. Sequences affiliated with *Methanobrevibacter* dominate, on average accounting for 61.6% of rumen archaea, with sequences associated with *M. gottschalkii* (33.6%) and *M. ruminantium* (27.3%) being most prominent [10].

Attempts have been made to inhibit the action of methanogens in the rumen using a variety of interventions but most have failed, or met with only limited success, due to low efficacy, poor selectivity, toxicity of compounds against the host, or build-up of resistance to anti-methanogen compounds [11]. Currently there are few practical methane reduction technologies available for housed ruminant animals, and no effective technologies for pasture-grazed animals, the main feeding system employed in NZ. Methane mitigation interventions should ideally target features that are conserved across all rumen methanogens, so that all methanogens are affected and no unaffected species can fill the vacated niche. Interventions should also be specific for methanogens so that other rumen microbes continue their normal digestive functions. We have embarked on a programme to sequence the genomes of cultured representatives of the main rumen methanogen groups to better understand this important group of organisms and to define their conserved and specific features that can serve as targets for CH_4_ mitigation technologies. Here we report the genome sequence of *M. ruminantium* M1^T^ (DSM 1093), the first rumen methanogen genome to be completely sequenced.

Defining gene targets within rumen methanogens for CH_4_ mitigation technologies is somewhat akin to developing a therapeutic intervention for a microbial pathogen, except that there are limitations in applying interventions to pasture-grazed ruminants. To be useful over an extended period in grazing animals, an intervention needs to be applied continually, to prevent methanogen recolonization, and be effective at low concentrations to overcome problems of intake by the animal and dilution within the rumen. Practically, this limits the type of intervention to either an immunological approach, in which animals are vaccinated and produce salivary antibodies against rumen methanogens which subsequently bind to and inhibit their action in the rumen, or to interventions based on chemical inhibitors or enzymes targeting essential methanogen functions which are delivered via slow-release capsules administered to the rumen. Therefore, our analysis of the M1 genome is presented with an emphasis on identifying conserved methanogen surface proteins suitable for vaccine development via reverse vaccinology (RV) techniques [12] and enzyme targets susceptible to small molecule inhibitors through a chemogenomics approach [13].

## Results

### General Genome Characteristics

**Figure 1:**
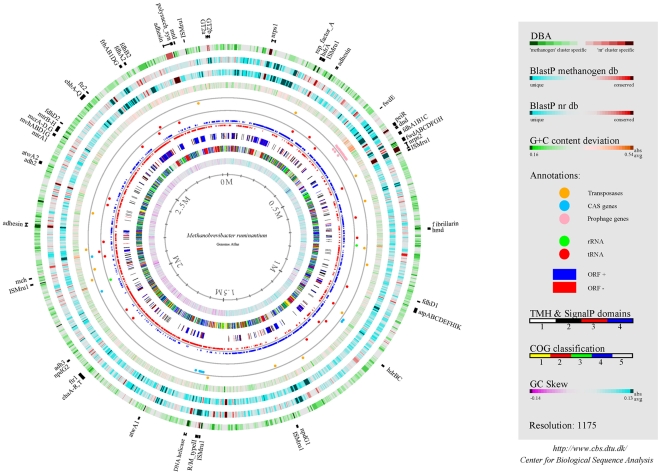
Genome atlas of M1. Single circles in the top-down- outermost-innermost direction are described. Outermost **1^st^ ring**: DBA between the nr database (Ring 3) and dbMethano, a custom methanogen database (Ring 2). Regions in green indicate protein sequences highly conserved between M1 and methanogens but not found in the nr database beyond methanogen genomes. Regions in red indicate protein sequences conserved between M1 and the nr database but not present in other methanogen genomes. **2^nd^ ring**: gapped BlastP results using dbMethano. **3^rd^ ring**: gapped BlastP results using the nr database minus published methanogen genome sequences. In both rings, regions in blue represent unique proteins in M1, whereas highly conserved features are shown in red. The degree of colour saturation corresponds to the level of similarity. **4^th^ ring**: G+C content deviation: green shading highlights low-GC regions, orange shading high-GC islands. Annotation **rings 5** and **6** indicate absolute position of functional features as indicated. **7^th^ ring**: ORF orientation. ORFs in sense orientation (ORF+) are shown in blue; ORFs oriented in antisense direction (ORF-) are shown in red. **8^th^ ring**: prediction of membrane bound and cell surface proteins. White: no transmembrane helices (TMH) were identified, Black: ORFs with at least one TMH, Red: ORFs predicted to encompass a signal peptide sequence and Blue: ORFs predicted to incorporate both TMH and a signal peptide sequence. **9^th^ ring:** COG classification. COG families were assembled into 5 major groups: information storage and processing (yellow); cellular processes and signalling (red); metabolism (green); poorly characterized (blue); and ORFs with uncharacterized COGs or no COG assignment (grey). **10^th^ ring**: GC-skew. Innermost ring: genome size (Mb). Selected features representing single ORFs are shown outside of circle 1 with bars indicating their absolute size.

The genome sequence of M1 consists of a single 2.93 megabase (Mb) circular chromosome, the assembly of which has been verified by pulsed-field gel electrophoresis (Figure S1). The general features of the M1 genome compared to other genomes of species within the order Methanobacteriales are summarized in Table 1 and Figure 1. M1 has the largest genome of the Methanobacteriales sequenced to date. This increased genome size is due in part to a lower overall coding density, but also to a large number of genes encoding surface adhesin-like proteins, the presence of a prophage, and a variety of genes unique to the M1 genome. M1 encodes 2217 open reading frames (ORFs) and a functional classification of each ORF is presented in Table S1 and Figure S2. Genomes of the Methanobacteriales display a GC skew similar to bacterial chromosomes [14] (Figure S3) and an X-shaped synteny pattern that is characteristic of moderately diverged genomes (Figure S4). Analysis of potential horizontal gene transfer (HGT) events in M1 identified a number of genes which show high sequence similarity to non-methanogens, typically from members of the bacterial phylum Firmicutes (Table S2). These potential HGT events can be visualized in a BLAST heat map analysis (Figure S5).

**Table 1:** Comparison of the M1 genome features with methanogens from the order Methanobacteriales.

	*M. ruminantium M1*	*M. smithii* PS [34]	*M. smithii* ALI^a^	*M. smithii* F1^a^	*Methanothermobacter thermoautotrophicus* ΔH [91]	*Methanosphaera stadtmanae* MCB-3 [20]
**Source**	Bovine rumen	Sewage digester	Human	Human faeces	Sewage sludge	Human faeces
**Project status**	complete	complete	draft	draft	complete	complete
**Genome size (bp)**	2,937,203	1,853,160	1,704,865	1,707,624	1,751,377	1,767,403
**G+C content (%)**	33	31	31	31	50	28
**Number of ORFs**	2217	1795	1709	1710	1873	1534
**Coding area (%)**	81	90	90	90	90	84
**rRNA operons**	2	2	nd	nd	2	4
**tRNAs (with intron)**	58 (2)	34 (1)	34	34	39 (3)	40 (1)
**Non-coding RNA**	3	3	nd	nd	2	2
**Insertion sequences**	4	8	nd	nd	0	4
**Prophage**	Yes	Yes	nd	nd	No	No
**CRISPR regions**	2	1	nd	nd	2	2
**Adhesin-like proteins**	105	48	nd	nd	0	37
**LPxTG motif**	1	2	nd	nd	0	0
**Sortases**	1	1	nd	nd	2	0

a Draft genome data obtained from National Centre for Biotechnology Information http://www.ncbi.nlm.nih.gov/;

Data not determined (nd) from draft genome.

### Growth and Methanogenesis

**Figure 2:**
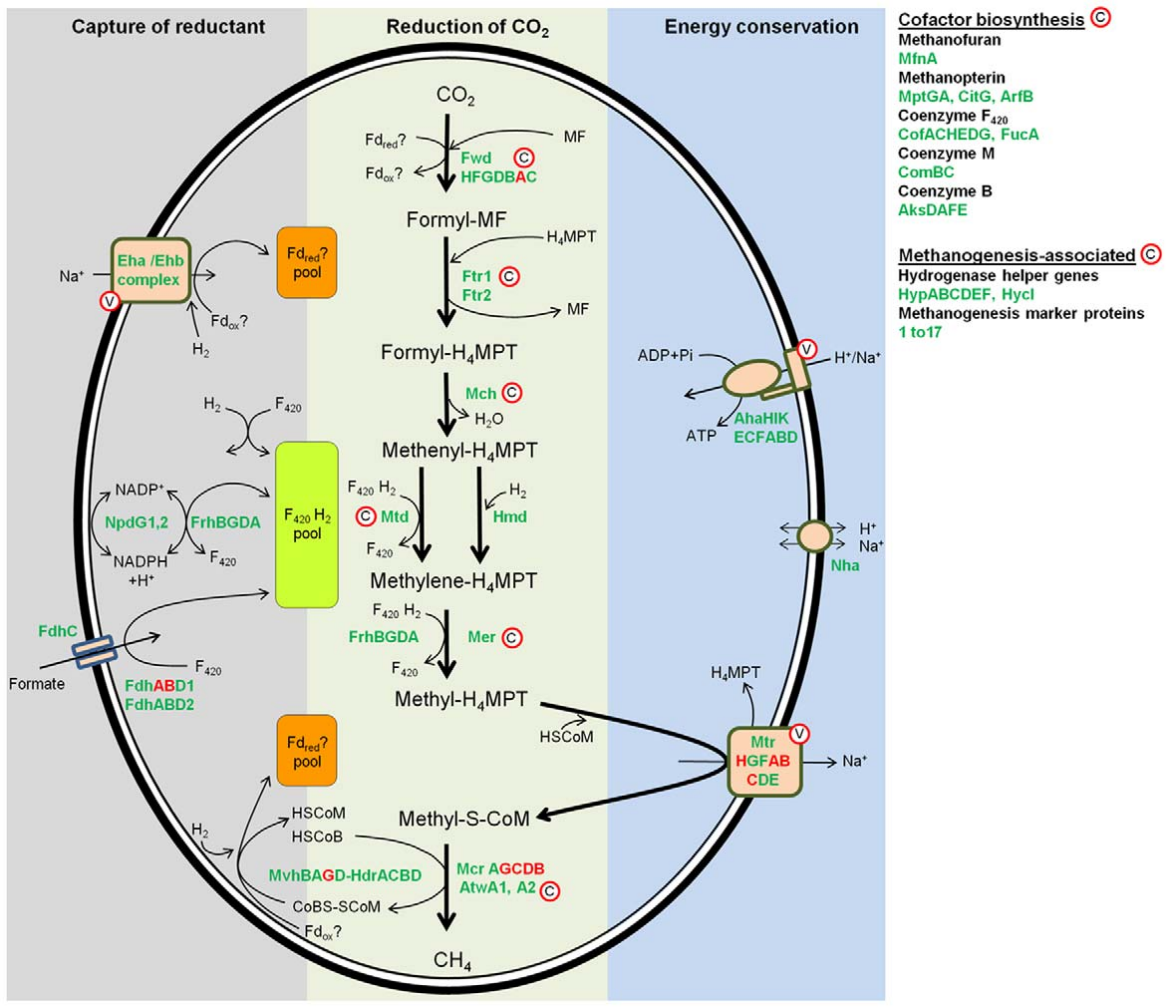
Methanogenesis pathway. The predicted pathway of methane formation in M1 based on the scheme of Thauer *et al.* [15] for methanogens without cytochromes is shown. The diagram is divided into three parts to show the capture of reductant, the reduction of CO_2_, and conservation of energy at the methyltransfer step. The main reactions are indicated by thick arrows and enzymes catalysing each step are coloured green. Protein subunits coloured red signify the corresponding genes that were up-regulated during co-culture with *Butyrivibrio proteoclasticus*. Cofactor participation is indicated with thin arrows. For simplicity, protons are not shown and the overall reaction is not balanced. Membrane-located proteins are contained in light brown boxes and potential vaccine and chemogenomic targets are labelled with a circled V or C, respectively. Full gene names and corresponding locus tag numbers can be found in Table S1. H_4_MPT; tetrahydromethanopterin; MF, methanofuran; F_420_, coenzyme F_420_ oxidised; F_420_H_2_, coenzyme F_420_ reduced; Fd_ox_?, unknown oxidised ferredoxin; Fd_red_?, unknown reduced ferredoxin; HSCoM, reduced coenzyme M; HSCoB, reduced coenzyme B, CoMS-SCoB, coenzyme B-coenzyme M heterodisulphide; NADP^+^, nicotinamide adenosine dinucleotide phosphate non-reduced; NADPH, nicotinamide adenosine dinucleotide phosphate reduced.

Many of the enzymes involved in the methanogenesis pathway are strongly conserved and found only among methanogens. Although this pathway has been well studied in methanogens from a range of other environments [15] the M1 genome shows for the first time details of this pathway in a rumen methanogen. M1 can grow with H_2_ plus CO_2_ and formate [16] and encodes the enzymes, and most of the cofactors, required for conversion of these substrates through to methane according to the metabolic scheme presented in Figure 2. Consistent with this hydrogenotrophic lifestyle, M1 lacks the methanophenazine-reducing [Ni-Fe] hydrogenase (VhoACG) and methanophenazine-dependent heterodisulphide reductase (HdrDE) found in methanophenazine-containing species within the order Methanosarcinales [17].

**Figure 3:**
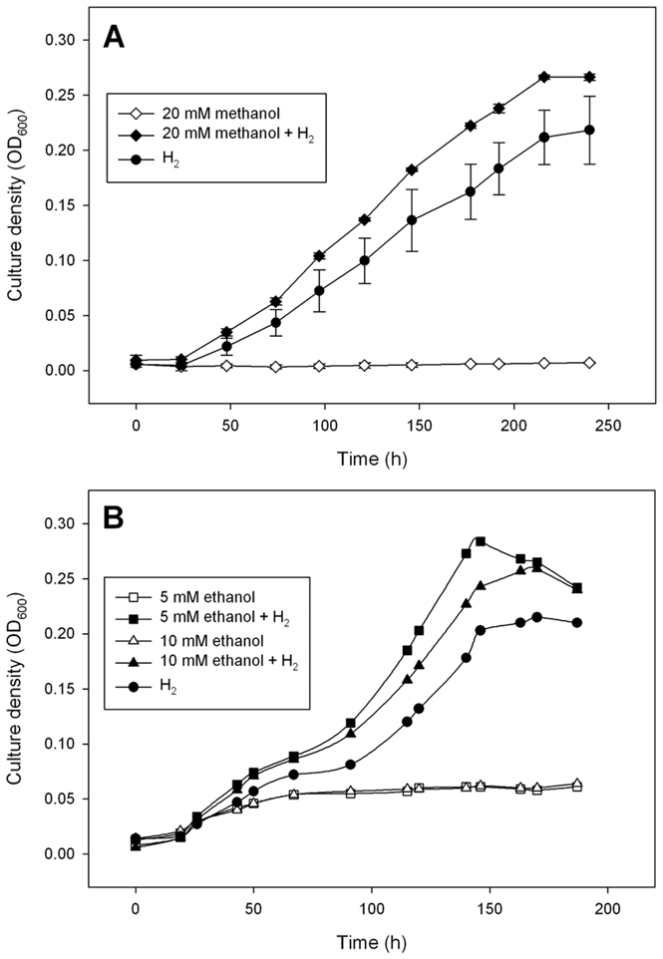
Stimulation of growth of M1 by alcohols. The inclusion of (A) 20 mM methanol or (B) 5 or 10 mM ethanol when M1 was grown on H_2_ resulted in an increase in culture density (measured as OD_600_ nm) compared to cultures grown on H_2_ alone. H_2_ was added once only, at the time of inoculation, by gassing the cultures with H_2_ plus CO_2_ (4∶1) to 180 kPa overpressure. Higher concentrations of ethanol (20 mM) resulted in some inhibition of growth (not shown), and there was no stimulation by isopropanol (5 to 20 mM; not shown). No growth occurred when cultures were supplemented with methanol (A), ethanol (B), or isopropanol (not shown) when no H_2_ was added, and no methane was formed by those cultures. The symbols in panel are means of 4 replicates, and the thin vertical bars in panel (A) represent one standard error on either side of the mean. Error bars are omitted from panel (B) for the sake of clarity.

Surprisingly, M1 has two NADPH-dependent F_420_ dehydrogenase (*npd*G1, 2) genes and three NADP-dependent alcohol dehydrogenase (*adh*1, 2 and 3) genes. In some methanogens, these enzymes allow growth on ethanol or isopropanol via NADP^+^-dependent oxidation of the alcohol coupled to F_420_ reduction of methenyl-H_4_MPT to methyl-H_4_MPT [18]. M1 is reported as not being able to grow on ethanol or methanol [16], although a ciliate-associated *M. ruminantium*-like isolate was able to use isopropanol to a limited degree but data were not presented [19]. Our attempts to grow M1 on alcohols indicate that ethanol and methanol stimulate growth in the presence of limiting amounts of H_2_+CO_2_, but they do not support growth when H_2_ is absent (Figure 3). M1 does not contain homologues of the *mta* genes known to be required for methanol utilization in other methanogens [20]. The *adh* genes may play a role in alcohol metabolism but the mechanism is unclear.

Hydrogenotrophic methanogens usually encode a methyl coenzyme reductase II (*mcr*II or *mrt*), an isoenzyme of the methyl CoM reductase I (*mcr*I) enzyme which is differentially regulated during growth [21] to mediate methane formation at high partial pressures of H_2_. Interestingly, M1 does not encode a *mcr*II system. In the rumen, methanogens depend on fermentative microbes to supply H_2_, usually at very low concentrations, and M1 appears to have adapted its lifestyle for growth at low levels of H_2_ using the *mcr*I system only.

To examine the expression of genes involved in methanogenesis, in the presence of a H_2_-forming rumen bacterium, M1 was grown in co-culture with *Butyrivibrio proteoclasticus* B316 [22] in a medium containing xylan as the sole carbon source, and gene expression analysed by microarrays. Formylmethanofuran dehydrogenase (*fwdA*), methyl CoM reductase (*mcrBCDG*), methyl viologen-reducing hydrogenase (*mvhG*), and H_4_MPT methyltransferase (*mtrABCH*) were significantly up-regulated (>2 fold) in the co-culture compared to the monoculture of M1 grown with H_2_+CO_2_ (Table S3). Interestingly, formate utilisation (*fdhAB*) genes were also up-regulated, suggesting that formate formed by *B. proteoclasticus* was an important methanogenic substrate transferred during this syntrophic interaction.

Analysis of the M1 genome has helped explain the growth requirements of M1 for acetate, 2-methylbutyrate and co-enzyme M (CoM) [23]. Acetate is required for cell carbon biosynthesis after activation to acetyl CoA (*acs*, *acsA*), followed by reductive carboxylation to pyruvate (*porABCDEF*, Table S1). Reductive carboxylation of 2-methylbutyrate is probably the route for isoleucine biosynthesis [24], as M1 lacks a gene encoding a homoserine kinase needed for the usual pathway from threonine (Table S1). Exogenously supplied CoM is essential for M1 growth as three genes needed in the CoM biosynthetic pathway, phosphosulfolactate synthase (*com*A) and sulfopyruvate decarboxylase (*com*D,E) [25], are missing in M1.

### Cell Envelope

**Figure 4:**
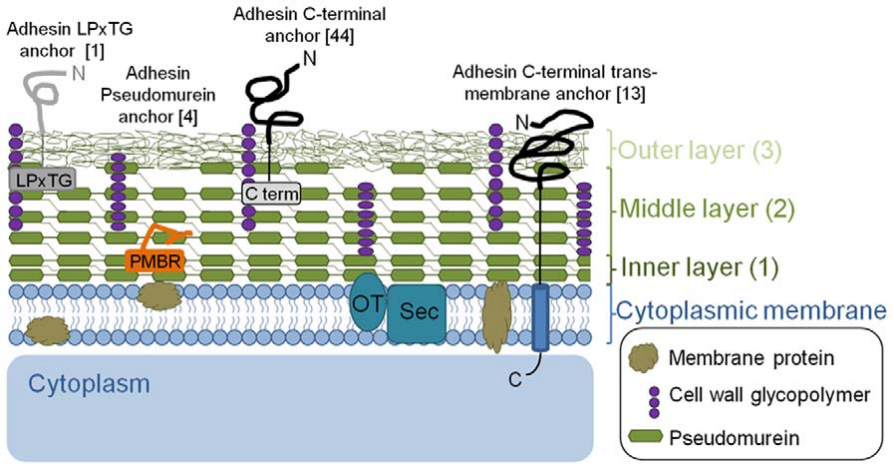
Putative cell envelope topography of M1. Ultrastructural studies of M1 [88], [89] show that the cell wall is composed of three layers and is comparable to the organization seen in Gram positive bacteria [90]. The three layers can be described as: (1) a thin electron-dense inner layer composed of compacted newly synthesised pseudomurein, (2) a thicker less-electron-dense middle layer which is also composed of pseudomurein, and (3) a rough irregular outer layer that is distal to the pseudomurein layers and assumed to be composed of cell wall glycopolymers, wall-associated proteins and possibly other components. Representative adhesin-like proteins with different cell-anchoring domains are shown. The number of these proteins predicted in the M1 genome is shown in brackets. OT, oligosaccharyl transferase; Sec, Sec protein secretion pathway; PMBR, pseudomurein binding repeat (PF09373); M1-C, M1 adhesin-like protein conserved C-terminal domain.

The methanogen cell envelope serves as the interface between the organism and its rumen environment, and as such represents a key area for the identification of vaccine and drug targets. The main structural component of the cell envelope of M1 (Figure 4), as with other Gram-positive methanogens, is pseudomurein. This is structurally analogous, but chemically different, to peptidoglycan, which performs the comparable function in bacteria [26]. Bacterial peptidoglycan biosynthesis has long been a major target of antimicrobials but these compounds are largely ineffective against pseudomurein-containing cells [27]. The pathway for pseudomurein biosynthesis and its primary structure have been proposed [27], but the enzymes involved have not been characterized. Our genomic analysis has identified several genes encoding enzymes likely to be involved both in the intracellular biosynthesis of the pseudomurein precursors and the processes involved in exporting and assembling these into the cell wall (Figure S6).

The original description of *M. ruminantium* reported the existence of a capsule surrounding the cells, and chemical analysis of the cell walls showed that galactose and rhamnose together with lower amounts of glucose and mannose were present in addition to pseudomurein [28], [29]. The cell walls are also reported to contain high levels of phosphate, comparable to that found in bacterial cell walls containing teichoic acid [28]. M1 contains homologues of genes involved in teichoic acid production in Gram-positive bacteria [30], [31] (Table S1), suggesting the presence of as-yet unidentified cell wall glycopolymers. Additionally, several genes are predicted to be involved in exopolysaccharide production, sialic acid biosynthesis and protein glycosylation (Table S1). The genome contains a homologue of the eukaryal oligosaccharyl transferase (mru0391), a membrane protein believed to be involved in glycosylating proteins translocated via the Sec pathway [32] (Figure 4). Glycoproteins derived from the cell wall of M1 have been shown to be highly immunogenic in sheep. The resulting antisera agglutinated M1 cells and significantly reduced their ability to grow and produce methane *in vitro* [33]. Overall, polysaccharides and glycosylated molecules are a major component of the M1 cell envelope, and their accessibility at the cell surface make these polymers viable methane mitigation targets.

**Figure 5:**
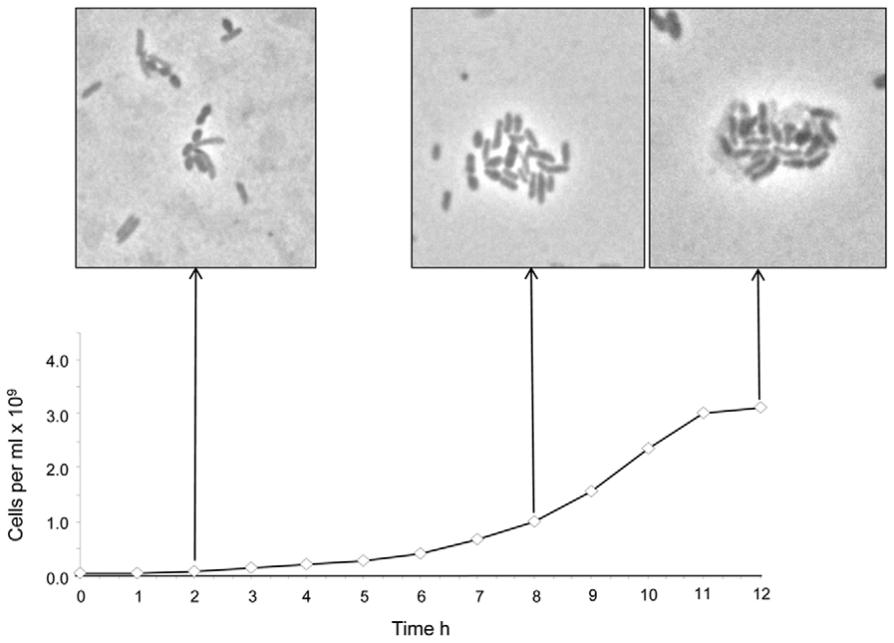
Observation of interspecies interactions between M1 and *B. proteoclasticus* B316. Graph displays growth rate of M1 in co-culture with B316. Microscopic images taken at 2, 8 and 12 h post innoculation of B316 (lighter, rod-shaped organism) into BY^+^ (+0.2% xylan) media containing a mid-exponential M1 culture (darker, short ovoid rod-shaped organism). Growth as determined by Thoma slide enumeration is shown along with sampling time.

**Figure 6:**
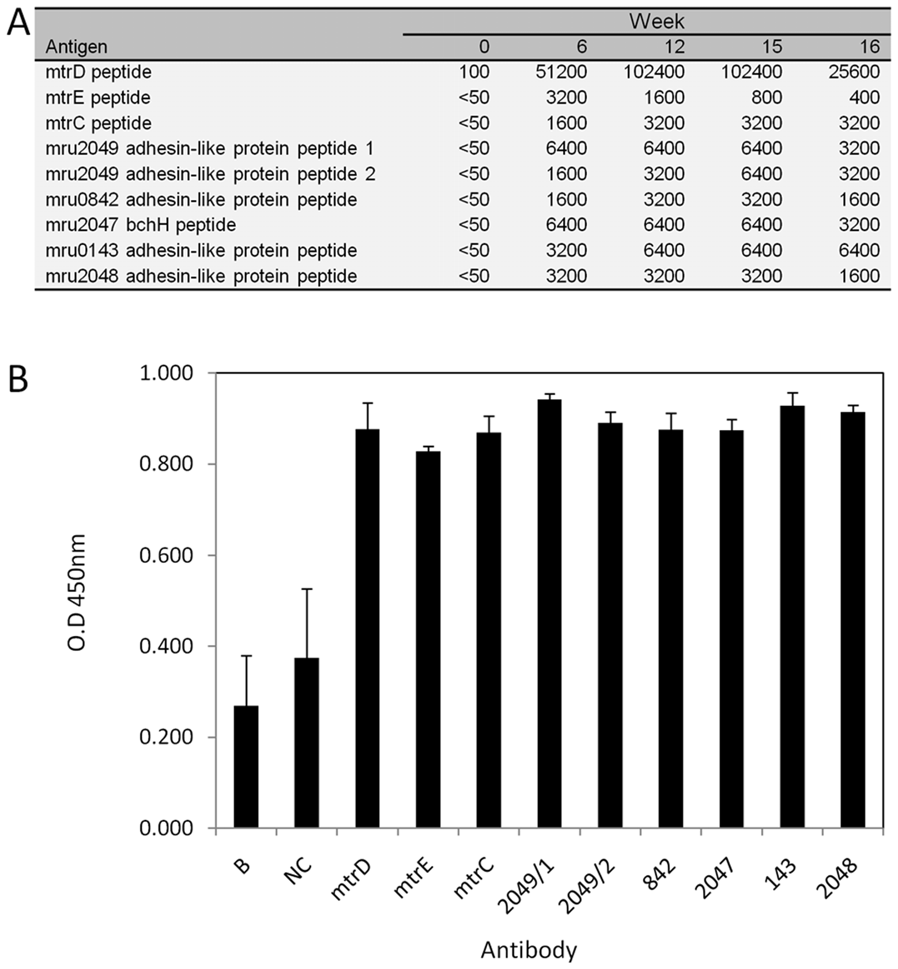
M1 peptide vaccine results. Sheep antibody responses to (A) vaccination with peptides designed against M1 genes (B) binding of antibodies to immobilised M1 cells. In the antibody-binding experiment a negative control (NC) serum from a sheep which had not had colostrum as a lamb was included, as was a sample without added serum which served as a blank, B.

Genomes of human gut methanogens encode large surface proteins that have features similar to bacterial adhesins [20], [34]. Similarly, M1 has an array of large adhesin-like proteins, much greater in number than those reported from other gut methanogens (Table 1). In the co-culturing experiments described above, six M1 adhesin-like proteins were upregulated (Table S3), and microscopic examination showed co-aggregation of M1 and *B. proteoclasticus* cells (Figure 5). In addition, immune sera produced by small peptides synthesized to correspond to four M1 adhesin-like proteins were shown to bind specifically to immobilized M1 cells (Figure 6). Identifying highly conserved methanogen-specific features of these adhesin-like proteins may present a pathway to vaccine development. Sixty-two adhesin-like proteins are predicted to be extracellular and contain a cell-anchoring domain (Figure 4). These proteins represent a significant component of the M1 cell envelope (Table S4). The largest group of these (44) contain a conserved C-terminal domain (M1-C, Figure S7) with weak homology to a Big_1 domain (Pfam accession number PF02369) which may be involved in attachment to the cell wall, possibly by interaction with pseudomurein or cell wall glycopolymers. Several of these proteins also contain a papain family cysteine protease domain (PF00112), and their role may be in the turnover of pseudomurein cell walls. A second group of 14 proteins is predicted to contain a C-terminal transmembrane domain, suggesting they are anchored in the cell membrane. Curiously, the genome contains one adhesin-like protein (mru2147) with a cell wall LPxTG-like sorting motif and three copies of a cell wall binding repeat (PF01473), both of which are commonly found in Gram-positive bacteria. There has only been one other report of a LPxTG-containing protein in a methanogen, the pseudomurein containing *Methanopyrus kandleri* [35]. Our analysis of the *M. smithii* PS genome revealed the presence of two LPxTG containing proteins (msm0173 and msm0411). Such proteins are covalently attached to the cell wall by membrane associated transpeptidases, known as sortases. Sortase activity has been recognised as a target for anti-infective therapy in bacteria [36] and a sortase (mru1832) has been identified in the M1 genome.

### Prophage

**Figure 7:**
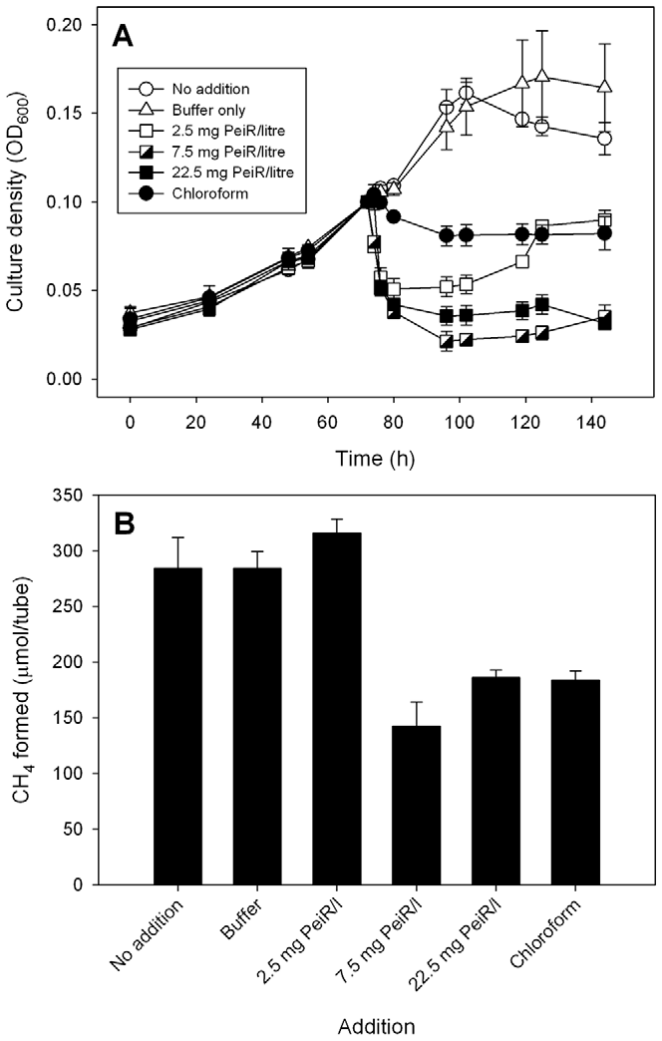
Effect of the lytic enzyme PeiR on M1 growth *in vitro*. (A) Addition of PeiR to growing cultures at 73 h resulted in a dramatic drop in culture density, indicative of cell lysis. At a low concentration of PeiR (final concentration of 2.5 mg per litre), the cultures were able to recover, indicated by the increase in culture density after 100 h, and (B) by production of methane at levels similar to that of cultures receiving no PeiR. Addition of higher concentrations of PeiR (7.5 and 22.5 mg per litre) resulted in a lasting effect, with (A) no subsequent recovery of growth and (B) a reduced methane yield. Chloroform, a known potent inhibitor of methanogens, resulted in a similarly reduced methane yield (B), but the decrease in culture density was less (A), as expected since it halts metabolism rather than lysing cells. PeiR was added to 10 ml cultures in 0.1 ml of buffer. The buffer alone had no effect. The symbols (A) and solid bars (B) are means of 3 replicates, and the thin vertical bars represent one standard error on either side of the mean.

Phage exert a significant ecological impact on microbial populations in the rumen, and have been suggested as biocontrol agents for rumen methanogens [37]. M1 has 70 ORFs (mru0256-0325) over a 62 Kb GC-rich (39% G+C content) region of the genome that encode a prophage genome, designated ϕ-mru. Based on a functional annotation, ϕ-mru is partitioned into distinct modules encoding integration, DNA replication, DNA packaging, phage capsid, lysis and lysogenic functions [38]. Within the lysis module, a gene encoding a putative lytic enzyme, endoisopeptidase PeiR (mru0320), was identified. Recombinant phage lytic enzymes have been used for controlling antibiotic-resistant bacterial pathogens [39], and a methanogen phage lytic enzyme may be a viable biocontrol option. We have confirmed the ability of recombinant PeiR to lyse M1 cells in pure culture (Figure 7) [40]. PeiR represents a novel enzyme, as it does not show significant homology to any sequence currently in public databases.

### Non-Ribosomal Peptide Synthetases

**Figure 8:**
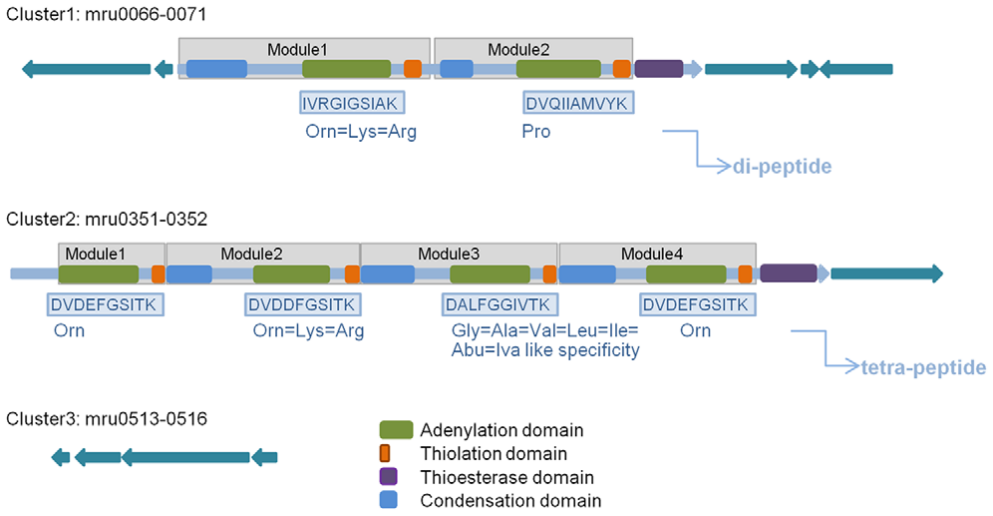
Organization of three gene clusters proposed to be involved in M1 NRP biosynthesis.

An unforeseen and novel feature of M1 is the presence of two large proteins (mru0068 and mru0351) showing the distinctive domain architecture of non-ribosomal peptide synthetases (NRPS) (Figure 8). NRPSs produce a wide variety of small molecule natural products that have biotechnological applications as peptide antibiotics, siderophores, immunosupressants or antitumor drugs [41]. The NRPS encoded by mru0068 is predicted to encode two modules, each containing condensation, adenylation and thiolation domains. The presence of a condensation domain in the first module is often associated with NRPSs that make *N*-acylated peptides [42]. The second module is followed by a terminal thioesterase domain which is thought to release the peptide from the final thiolation domain. Mru0068 is surrounded by genes that encode two serine phosphatases (mru0066, mru0071), an anti-sigma factor antagonist (mru0067), and a MatE efflux family protein (mru0069), which are likely to be involved in environment sensing, regulating NRPS expression and export of the NRP, respectively. Mru0068 displays full length protein alignment with a putative NRPS from *Syntrophomonas wolfei* subsp. *wolfei* strain Göttingen (Figure S8), a Gram-positive bacterium known to participate in syntrophic interactions with methanogens [43]. The second NRPS gene (mru0351) contains 4 modules and a thioesterase domain. Downstream of mru0351 is another MatE efflux family protein (mru0352), presumably involved in NRP export. A third, smaller cluster of genes located elsewhere in the genome (mru0513-0516) appear to encode NRPS-associated functions. This cluster includes a 4′-phosphopantetheinyl transferase (mru0514) which primes NRPSs by adding a phosphopantetheinyl group to a conserved serine within the thiolation domain, an acyltransferase (mru0512) possibly involved in NRP acylation, a serine phosphatase (mru0515), an anti-sigma factor antagonist (mru0513), and an anti-sigma regulatory factor serine/threonine protein kinase (mru0516) that may function in sensing the environment and NRPS regulation. Although the products of each NRPS are unknown, an analysis of adenylation domain amino acid sequences predicts 10 residues (boxed, Figure 8) which are important for substrate binding and catalysis. HGT studies indicate that these genes may be bacterial in origin (Table S2).

### Identification of Targets for Methane Mitigation

**Figure 9:**
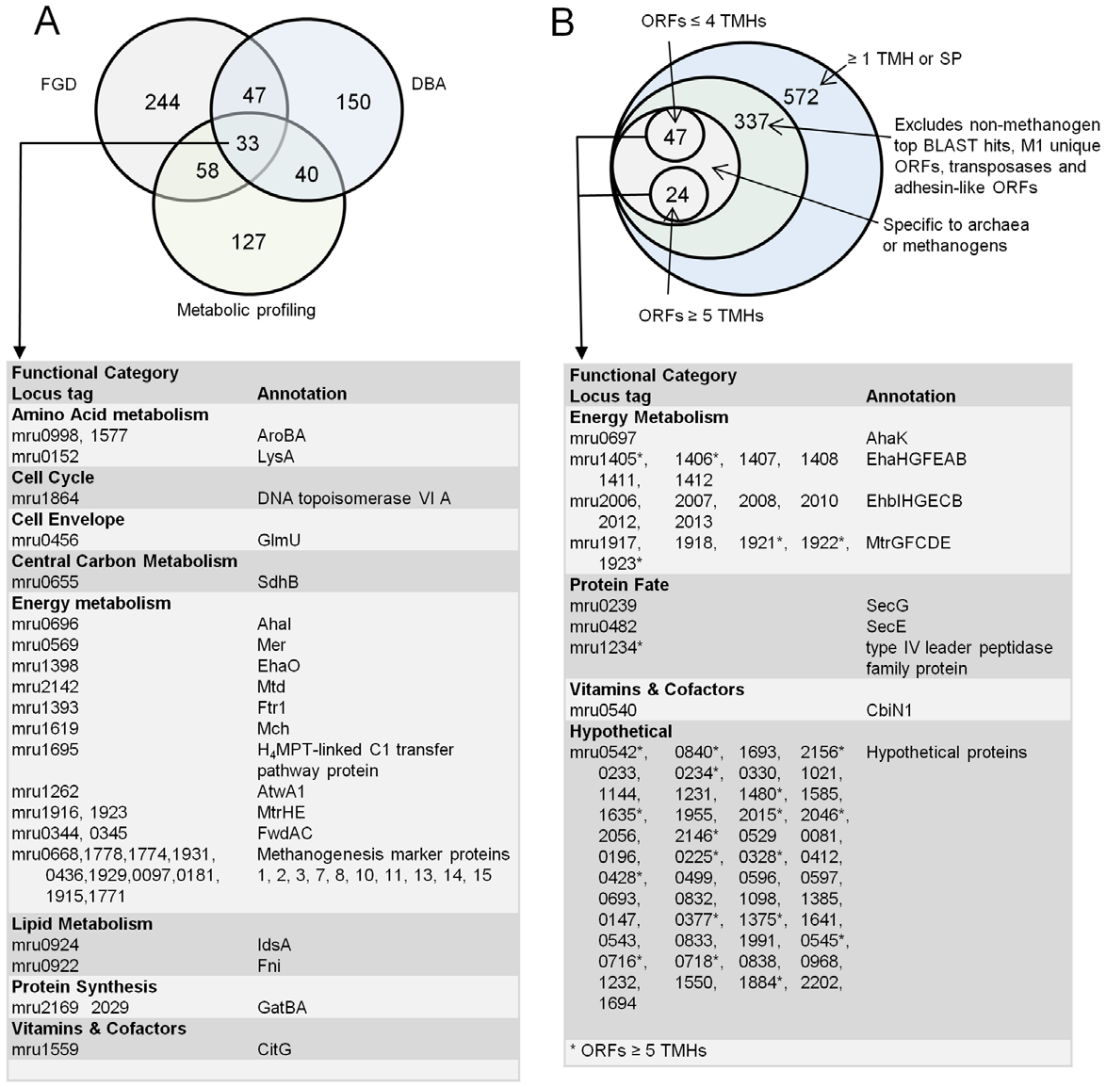
Chemogenomic and vaccine gene targets within M1. The number of genes identified by each analysis is shown in the Venn diagram and a selection of the gene targets are summarized in the boxes grouped by functional category. (A) Chemogenomic gene targets were defined by identification of genes that occurred across three separate analyses: the Functional Genome Distribution (FGD), Differential BLAST Analysis (DBA), and metabolic profile of M1 (b) Vaccine target genes were defined as described in Materials and Methods. TMH, transmembrane helices, SP, signal peptide.

Several approaches were used to define potential gene targets from M1 for CH_4_ mitigation via chemogenomic and vaccine approaches (Figure 9). Genes suitable as chemogenomic targets were identified using a combination of metabolic profiling, review of the literature pertaining to the biochemistry and physiology of methanogens, and comparative genomics. The 33 candidate genes commonly identified by these approaches are shown in Figure 9A. The full list of ORFs identified as chemogenomic targets by metabolic profiling of M1 and literature can be found in Table S5. Comparative studies were based on M1 and 26 complete and draft phase methanogen genome sequences, using a functional genome distribution (FGD) analysis (Table S6, Figure S9). This analysis of whole genome gene conservation among methanogens showed that M1 and other members of the Methanobacteriales formed a functional cluster that shared a large number of conserved genes predicted to be involved in core biological functions (low e-value cut-off 1e-100, Table S6). In addition, a differential blast analysis (DBA) was conducted using the non-redundant (nr) database and a methanogen genome sequence database (dbMethano). The DBA analysis highlighted genes present in at least one methanogen genome within dbMethano but not present in any other organism within the nr database and vice versa (Figure 1), thus identifying methanogen-specific genes. The majority of the 33 selected conserved and methanogen-specific genes encode enzymes that fall within the energy metabolism category, mainly within the methanogenesis pathway (Table S1). This also included several methanogenesis marker proteins found in methanogen genomes, but currently without defined function. Most of these methanogenesis enzymes are located within the cell cytoplasm, and therefore have been tagged as key targets for inhibitor discovery via a chemogenomics approach (Figure 2).

The alternative approach of inducing the ruminant immune system to produce salivary antibodies against conserved features of rumen methanogens is an attractive methane mitigation strategy. The rumen epithelium is not immunologically active, and rumen contents do not contain complement proteins, therefore specific immune responses in the rumen do not occur. The effectiveness of a vaccination approach relies on the binding of salivary antibodies to methanogen surface features which results in their inactivation or clearance from the rumen. Vaccines are typically composed of proteins or polysaccharides derived from killed or attenuated whole cells or components presented on the outside of the cell such as flagella, capsules, cell walls, fimbrae, or secreted toxins. In the case of rumen methanogens, the primary vaccine targets are likely to be surface-exposed or membrane-associated proteins that are conserved among methanogens and which encode functions vital to methanogen growth and survival in the rumen. *In silico* analysis of the M1 ORFeome (all ORFs) identified an initial pool of 572 ORFs containing one or more transmembrane helices (TMH) or signal peptide (SP) indicating a cell membrane or cell surface location and therefore potential vaccine targets. Those ORFs with a top BLAST hit to a non-methanogen or with no homology to the nr database were removed from the analysis, as were transposase sequences (which are unlikely to represent good vaccine targets), while adhesin-like ORFs are dealt with separately above. This gave a new total of 337 ORFs. Examination of the remaining 337 ORFs, assessing their predicted function, degree of conservation among methanogens and the nature of their transmembrane structures, refined the list to 71 ORFs (Figure 9B). Heterologous expression of membrane proteins with more than 4 TMHs has been difficult in RV studies of other microbes [44]. Therefore, a cut-off of 4 THMs was applied to define two final groups: Group A with 47 ORFs with 4 or fewer TMHs suitable for cloning and heterologous expression studies; and Group B composed of 24 ORFs with more than 4 TMHs more suitable for a synthetic peptide-directed vaccine approach.

The majority of vaccine targets identified above correspond to hypothetical proteins of unknown function. While these ORFs are presumed to be of value to M1, their importance to M1 growth and survival in the rumen is not evident, and therefore they are of lower priority as vaccine candidates. Of the remaining ORFs, those involved in energy metabolism are again prime vaccine candidates (Figure 2). Of particular interest is the Mtr enzyme complex, which catalyses the essential methanogen function of transferring the methyl group from methyl-H_4_MPT to CoM, coupled to the efflux of Na^+^ ions [45]. Three of the Mtr subunits (MtrEDC) are each predicted to have >4 membrane-spanning regions and, in each of the membrane-spanning regions, the transmembrane helices have peptide loops located outside the cell membrane. These loops are potential antibody binding sites. We synthesised peptides corresponding to the loop regions of MtrE, MtrD and MtrC which were coupled to a carrier protein and then used as antigens to vaccinate sheep. The resulting immune sera bound specifically to immobilized M1 cells (Figure 6), demonstrating the feasibility of such a peptide-directed RV approach.

## Discussion

The analysis of the M1 genome has provided new perspectives on the lifestyle and cellular processes of this prominent rumen methanogen. The genome sequence confirms the hydrogenotrophic lifestyle of M1 and gene expression data indicate that formate may be an important substrate for methanogenesis during syntrophic interaction with *B. proteoclasticus*. The ability of short chain alcohols to stimulate growth on H_2_ but not support growth themselves is intriguing. We speculate that methanol or ethanol are oxidised by the NADP-dependent alcohol dehydrogenases and the reducing potential used to form F_420_H_2_ using NADPH-dependent F_420_ dehydrogenase, thus augmenting the cellular pool of F_420_H_2._ This metabolism of alcohols could spare some of the H_2_ or formate normally used to produce F_420_H_2_ and would explain the stimulation of growth by alcohols in the presence of H_2._ The lack of a means of reducing ferredoxins with electrons from alcohols explains why growth is not possible on alcohols alone. Further work is required to test this hypothesis.

The abundance of genes encoding adhesin-like proteins in M1 indicates a significant ability to modulate cell surface topology. While the exact role of these proteins is currently unknown, initial observations from co-culture experiments indicate that at least some are involved in mediating close associations with hydrogen-producing bacteria in the rumen and others may be concerned with similar interactions with rumen protozoa and fungi.

The ϕ-mru prophage sequence within the M1 genome yielded the PeiR enzyme which is able to lyse methanogen cells. The variety of methanogen cell wall types means a combination of different lytic enzymes would be required for effective methanogen lysis in the rumen. However, the expression of PeiR and demonstration of its effectiveness against a major rumen methanogen is an important step towards this goal. The PeiR enzyme and the ϕ-mru phage may also be useful in increasing the permeability of M1 and other pseudomurein-containing methanogens to facilitate DNA entry and for developing tools for genetic manipulation of M1.

Methanogens are not known as producers of secondary metabolites, so the discovery of two NRPS genes was surprising, and to our knowledge, they are the first reported in an archaeal genome. Non-ribosomal peptides (NRPs) are known to contribute to microbial growth and ecological interactions and therefore their function is of interest as they could lead to a means of modulating methanogen growth.

The metabolic profiling and comparative genomics carried out in this study identified several sets of conserved, methanogen-specific genes that are currently being investigated further in our laboratory. Chemogenomic targets are being investigated via heterologous expression of genes in *Escherichia coli* coupled with the development of bioassays for screening these enzymes against libraries of chemical compounds to find specific inhibitors with efficacy at low concentrations. Vaccine candidate proteins with <4 TMHs are being investigated via heterologous expression in *E. coli* and vaccination of sheep. We have also shown the use of synthetic peptides in a reverse vaccinology approach to elicit specific antibody responses against M1 proteins. This demonstrates that membrane-embedded M1 proteins, that are unlikely to be amenable to expression in a heterologous host, are viable targets as vaccine antigens.

A wider representation of rumen methanogen genomes will be essential to verify that the selected vaccine and chemogenomics targets are conserved among other rumen methanogens, and ensure a successful, long-term CH_4_ mitigation technology for the rumen. The wealth of biological information provided by the M1 genome represents a starting point from which ruminant methane mitigation efforts, aimed at identifying anti-methanogen technologies with broad efficacy can begin.

## Materials and Methods

### Strain Information and Growth Conditions

*Methanobrevibacter ruminantium* M1^T^ (DSM1093) was obtained from the German Collection of Microorganisms and Cell Cultures (DSMZ), Braunschweig, Germany. The original description of *Methanobacterium ruminantium* was made by Smith and Hungate [16] and the genus designation later changed to *Methanobrevibacter* [46]. *Methanobrevibacter ruminantium* M1^T^ (DSM1093) was isolated from bovine rumen contents by Bryant [47]. It is designated the neotype strain for this species because the original strain of Smith and Hungate was not maintained. *M. ruminantium* strain M1^T^ was routinely grown in basal medium [48] with added trace elements [46] (BY^+^ medium), with H_2_ plus CO_2_ (4∶1) at 180 kPa overpressure. The culture tubes were incubated on their sides, at 39°C in the dark, on a platform shaken at 200 rpm.

### Co-Culture of *M. ruminantium* and *Butyrivibrio proteoclasticus*

M1 was grown in co-culture with *Butyrivibrio proteoclasticus* B316^T^ (DSM14932) to examine gene expression under rumen-like conditions. Eighteen pure cultures of M1 were grown in BY^+^ medium with H_2_ plus CO_2_ (4∶1) at 180 kPa overpressure in 100 ml volumes in 125 ml serum bottles sealed with blue butyl septum stoppers and aluminium seals (Bellco Glass, Vineland, NJ, USA). When the cultures reached mid-exponential phase, as measured by optical density at 600 nm (Ultrospec 1100 pro UV/Vis spectrophotometer, Amersham Biosciences, Little Chalfont, Buckinghamshire, UK) they were flushed with O_2_-free 100% CO_2_ gas until H_2_ was not detectable by gas chromatography. All 18 cultures were supplemented with oat spelt xylan (Sigma-Aldrich, St. Louis, MO, USA) to 0.2% (w/v) final concentration, then nine of the cultures were inoculated with 0.5 ml of a late-exponential phase culture of *B. proteoclasticus.* The other nine were re-pressurized to H_2_ plus CO_2_ (4∶1) at 180 kPa overpressure. Three further serum bottles of BY^+^ medium supplemented with 0.2% (w/v) xylan were also inoculated with an equivalent inoculum of *B. proteoclasticus*. Growth in the co-culture was monitored periodically by Thoma slide enumeration (Webber Scientific International Ltd., Teddington, England). Mid-exponential phase co-cultures and monocultures were harvested by centrifugation (10,000×*g*, 5 min at 4°C), and the cell pellets resuspended in 10 ml of BY^+^ medium (+ 0.2% [w/v] xylan) and 20 ml of RNAprotect (Qiagen, Hilden, Germany) and incubated for 5 min at room temperature, and were immediately processed for RNA extraction.

### Microarray Analyses

#### RNA isolation, cDNA synthesis and labeling

Cells of M1 and *B. proteoclasticus* from mono- or co-cultures prepared as described above, were pelleted by centrifugation (5,000×*g*, 10 min room temperature), air-dried and frozen under liquid N_2_. Frozen pellets were ground in a sterile pre-chilled (−20°C) mortar and pestle under liquid N_2_, and the ground samples resuspended in excess TRIzol (Invitrogen, Carlsbad, CA, USA). The mixtures were incubated at 20°C for 5 min. Chloroform (200 µl) was then added, mixed vigorously, and incubated for a further 3 min. The samples were centrifuged (12,000×*g*, 15 min, 4°C) and the aqueous phases transferred to fresh tubes, mixed with 0.5 volumes isopropanol and incubated at 20°C for 10 min to precipitate the RNAs. Precipitated RNAs were pelleted by centrifugation (12,000×*g*, 10 min, 4°C), the supernatants removed and the RNAs washed with 5 ml of 75% (v/v) ethanol before being re-pelleted by centrifugation. Ethanol was removed, the pellets air dried on ice and finally each resuspended in 1 ml of diethyl pyrocarbonate (DEPC) treated Milli-Q water. The RNAs were further purified using an RNeasy Midi kit (Qiagen, Hilden, Germany) and quantified using an Agilent 2100 Bioanalyzer (Agilent Technologies, Santa Clara, CA, USA) following the respective manufacturer's instructions. cDNA synthesis, labeling and purification were carried out using the Invitrogen cDNA labelling purification kit, while the Cy3 and Cy5 dyes were from GE Healthcare (Uppsala, Sweden).

#### Quantification of co-culture mRNA

The relative quantities of RNAs contributed by each organism to the co-culture samples were determined by quantitative PCR of the *B. proteoclasticus* butyryl-CoA dehydrogenase (*bcd*) gene (using primers bcdq*fp*: tgagaagggaacacctggat, and bcdq*rp*: ttgctcttccgaactgctt), and the M1 gene encoding N^5^,N^10^-methenyl-H_4_MPT cyclohydrolase (*mch*) (using primers mchq*fp*: gtattgcctggtgaagatgt and mchq*rp*: gtcgatttggtagaagtca). Homologues of both genes have previously been shown to be constitutively expressed in closely related species [21], [49]. The mono-culture RNAs were then combined in equal proportions to normalise mRNA abundance with their co-culture replicates.

#### Probe synthesis and slide printing

Oligonucleotide 70mer probes were designed against the draft genomes of M1 and *Butyrivibrio proteoclasticus* B316^T^ using ROSO software [50] and synthesised by Illumina (San Diego, CA, USA). Oligonucleotides were spotted onto epoxy-coated slides (Corning, Lowell, MA, USA) using an ESI robot (Engineer Service Inc., Toronto, Ontario, Canada).

#### Microarray hybridization and scanning

Microarrays were replicated 6 times (3 biological replicates per treatment, each with a dye swap) and each gene was represented on the array 3 to 7 times. Microarray slides were pre-warmed in microarray prehybridization buffer (50°C for 30 min), and transferred into hybridization chambers (Corning, Lowell, MA, USA) and lifter cover slips (Erie Scientific, Portsmouth, NH, USA) were laid over the probe areas. Samples of RNA to be compared (e.g., Cy3 co-culture versus combined Cy5 individual mono-cultures) were combined, denatured at 95°C for 10 min, and mixed with 60 µl of pre-warmed (68°C) Slide Hyb buffer #1 (Ambion, Austin, TX, USA). The mixture was loaded onto the slide, the hybridization chamber sealed, and incubated in a water bath at 50°C for 24 h. Following hybridization, the slides were washed by vigorous shaking by hand in pre-warmed (50°C) wash solutions 1 to 3 (wash solution 1∶ 10%SDS, 2× SSC; wash solution 2∶ 1× SSC; wash solution 3∶ 0.1× SSC), 7 min per wash in aluminium foil-covered Falcon tubes (Becton, Dickinson and Co. Sparks, MD, USA). Following the third wash, the slides were dried by low speed centrifugation (1,500 ×*g*, 4 min) followed by incubation for 20 min in a 37°C vacuum oven (Contherm, Wellington, NZ) in the dark. Microarray slides were scanned using a GenePix® Professional 4200 scanner and GenePix Pro 6.0 software (Molecular Devices, Sunnyvale, CA, USA) and analysed using the Limma package in Bioconductor [51]. Genes with an up- or down-regulation of 2-fold or greater and an FDR value <0.05 were deemed statistically significant. Microarray data has been submitted to the Gene Expression Omnibus (GEO) in accordance with MIAME standards under GEO accession number GSE18716.

### Growth Experiments to Test Effects of PeiR and Alcohols

M1 was grown in medium RM02 in anaerobic culture tubes (16 mm internal diameter, 18 mm outer diamater, 150 mm long; Bellco Glass, Vineland, NJ, USA), essentially as described by Balch and Wolfe [52]. The mineral salts base of RM02 contained (per litre of medium): 1.4 g of KH_2_PO_4_, 0.6 g of (NH_4_)_2_SO_4_, 1.5 g of KCl, 1 ml trace element solution SL10 [53], 1 ml of selenite/tungstate solution [54] and 4 drops of 0.1% (w/v) resazurin solution. This solution was mixed and then boiled under O_2_-free 100% CO_2_, before being cooled in an ice bath while it was bubbled with 100% CO_2_. Once the medium was cool, 4.2 g of NaHCO_3_ and 0.5 g of L-cysteine·HCl·H_2_O was added per litre. The medium was dispensed into the culture tubes while being gassed with 100% CO_2_, at 9.5 ml of medium per tube, and the tubes sealed with blue butyl septum stoppers and aluminium seals (Bellco), with a headspace of 100% CO_2_. These tubes were sterilised by autoclaving for 20 min at 121°C. Before use, the tubes were stored in the dark for at least 24 h. Sodium acetate (20 mM final conc.), sodium formate (60 mM final conc.), coenzyme M (10 µM final conc.), and vitamin-supplemented clarified rumen fluid were added to sterile media, before inoculation with 0.5 ml of an actively growing culture of *M. ruminantium*, then gassed with H_2_ plus CO_2_ (4∶1) to 180 kPa overpressure. In some experiments, the formate was omitted, and alcohols were added, as noted in the experimental descriptions accompanying the results. The culture tubes were incubated on their sides, at 39°C in the dark, on a platform shaken at 200 rpm.

To prepare the clarified rumen fluid, rumen contents were collected from a ruminally-fistulated cow that had been fed hay for 48 h after being on a ryegrass/clover pasture. Feed was withheld from the animal overnight and rumen contents collected the next morning. The material was filtered through a single layer of cheesecloth and then fine particulate material removed by centrifugation at 10,000×*g* for 20 min. The supernatant was stored at −20°C. Before further use, it was thawed, and any precipitates removed by centrifugation at 12,000×*g* for 15 min. The supernatant was bubbled for 10 min with 100% N_2_ gas, before being autoclaved under 100% nitrogen for 15 min to remove viruses. The following was then added per 100 ml of rumen fluid while stirring under air: 1.63 g of MgCl_2_·6H_2_O and 1.18 g of CaCl_2_·2H_2_O. The resulting heavy precipitate was removed by centrifuging at 30,000×*g* and 4°C for 60 min. The supernatant was designated the clarified rumen fluid. Two grams of yeast extract powder was added, and the mixture then bubbled with N_2_ gas for 15 min, before being transferred to a N_2_-flushed sterile serum vial through a 0.2-µm pore size sterile filter using a syringe and needle. Two ml of Vitamin 10 concentrate was then added per 100 ml of preparation by syringe and needle.

Vitamin 10 concentrate contained 1000 ml of distilled water, 40 mg of 4-aminobenzoate, 10 mg of D-(+)-biotin, 100 mg of nicotinic acid, 50 mg of hemicalcium D-(+)-pantothenate, 150 mg of pyridoxamine hydrochloride, 100 mg of thiamine chloride hydrochloride, 50 mg of cyanocobalamin, 30 mg of D,L-6,8-thioctic acid, 30 mg of riboflavin and 10 mg of folic acid. After preparation, the solution was well mixed and then bubbled with N_2_ gas for 15 min, before being transferred to a N_2_-flushed sterile serum vial through a 0.2 µm pore size sterile filter using a syringe and needle.

Growth of M1 was followed by measuring the culture density at 600 nm by inserting the tubes directly into an Ultrospec 1100 pro UV/Vis spectrophotometer (Amersham Biosciences, Little Chalfont, Buckinghamshire, UK). Tubes containing 10 ml of medium RM02 were inoculated with 0.5 ml of an actively growing culture of M1, then gassed with H_2_ plus CO_2_ (4∶1) to 180 kPa overpressure. Additions of PeiR in 0.1 ml of buffer (20 mM 3-[N-morpholino]propane sulfonic acid: 1 mM dithiothreitol: 0.3 M NaCl, 20% glycerol [v/v], pH 7.0 with NaOH), 0.1 ml of buffer only, or 0.1 ml of chloroform were made when the cultures had grown to mid-exponential phase (optical density at 600 nm [OD_600_] ∼0.1, 16 mm path length). In the experiments testing the effects of PeiR addition, the culture densities were mathematically normalised to an OD_600_ of 0.1 at the time the additions were made, and all other readings corrected by the same ratio. This was done to remove the effect of lack of absolute synchronicity between cultures, a common phenomenon when culturing methanogens. This normalisation was not done for experiments testing the utilisation of alcohols. Methane was measured by gas chromatography, taking a 0.3 ml sample from the culture headspace, at the pressure in the culture tube, and injecting it into an Aerograph 660 (Varian Associates, Palo Alto, CA, USA) fitted with a Porapak Q 80/100 mesh column (Waters Corporation, Milford, MA, USA) and a thermal conductivity detector operated at 100°C. The column was operated at room temperature with N_2_ as the carrier gas at 12 cm^3^/min.

### DNA Extraction

Genomic DNA was extracted from M1 grown on BY^+^ medium with H_2_ plus CO_2_ (4∶1), using the liquid N_2_ freezing and grinding method of Jarrell *et al.* [55]. Briefly, M1 cultures were harvested by centrifugation at 27,000×*g* for 20 min at 4°C and cell pellets combined and placed into 40 ml Oakridge centrifuge tubes (Thermo Fisher Scientific, Inc.). The cells were frozen at −20°C and kept frozen for at least 4 days. The frozen cell pellets were placed in a sterile, pre-cooled (−85°C) mortar and liquid N_2_ poured over the pellet. After the N_2_ had evaporated, the pellet was ground to a powder with a sterile glass rod. Immediately, 0.5 ml of TES buffer (10 mM Tris-HCl∶1 mM EDTA∶0.25 M sucrose, pH 7.5) was added to the powdered cell pellet and mixed gently into a slurry. Sodium dodecyl sulfate was added to a final concentration of 1% (w/v) and Proteinase K (Roche Diagnostics, Mannheim, Germany) added to a final concentration of 50 µg/ml. The mixture was incubated at 60°C for 30 min. NaCl was added to a final concentration of 0.5 M and the lysate was placed on ice for 1 h. The lysate was centrifuged at 25,000×*g* for 15 min at 4°C and the supernatant recovered carefully. An equal volume of cold (4°C) isopropanol was added to the supernatant, and the precipitated DNA was collected by centrifugation at 12,000×*g* for 10 min at room temperature and re-dissolved in TE buffer (10 mM Tris-HCl∶1 mM EDTA, pH 7.5). The DNA was treated with RNase (10 µg/ml), (Sigma-Aldrich) for 30 min at 37°C, and extracted twice with an equal volume of phenol/chloroform/isoamyl alcohol (25∶24∶1) and twice with an equal volume of chloroform alone. NaCl was added to a final concentration of 0.5 M and the DNA precipitated by adding 2.5 volumes of cold (4°C) ethanol. The precipitated DNA was collected by centrifugation at 14,000×*g* for 10 min at 4°C and re-dissolved in TE buffer.

### Pulsed-Field Gel Electrophoresis (PFGE)

Standard PFGE protocol involves embedding cells in agarose and lysis with lysozyme and/or proteases, but this was not possible with M1 because its pseudomurein-containing cell wall was resistant to lysis by commercially available enzymes. In order to overcome this, the cell pellet from a centrifuged 50 ml culture was frozen with liquid N_2_ and very gently ground in a pestle and mortar to damage the cell wall. The ground material was allowed to thaw, 2 ml of 1 M NaCl plus 10 mM Tris (pH 7.6) was added and 300 µl aliquots were mixed with an equal volume of 2% (w/v) low melt agarose (Bio-Rad Laboratories, Hercules, CA, USA). Embedded cells were treated with 0.1 mg/ml Proteinase K in lysis buffer (50 mM Tris-HCl∶50 mM EDTA∶1% [w/v] sarkosyl, pH 8.0) at 50°C for up to 24 h. The agarose plugs were washed twice with sterile water and three times with TE buffer (10 mM Tris-HCl∶1 mM EDTA, pH 8.0) before storage in 10 mM Tris-HCl∶100 mM EDTA (pH 8.0) at 4°C. DNA embedded in agarose was digested for 16 h with 1.0 U of ApaI, BssHII or MluI (New England Biolabs, Beverly, MA, USA) in 100 µl of restriction enzyme buffer, loaded into wells of 1% (w/v) agarose gels (SeaKem Gold agarose, Cambrex Bio Science, Rockland, ME, USA), and run at 200 V for 20 h at 14°C in 0.5X Tris-borate buffer using a CHEF DR III PFGE apparatus and model 1000 mini chiller (Bio-Rad). Double-digest combinations of these enzymes were digested and run in the same way. DNA was visualized by staining with ethidium bromide and the image captured using a Gel Doc 1000 system (Kodak Gel Logic 200 Imaging System, Eastman Kodak, Rochester, NY, USA).

### Genome Sequencing, Assembly and Validation

The genome sequence of M1 was determined using a whole genome shotgun strategy (Agencourt Biosciences, USA) and a pyrosequencing approach (Macrogen, USA). A hybrid assembly [56] was performed utilising the Staden package [57], Phred [58], Phrap (http://www.phrap.org), Paracel (Paracel Inc.) and Repeatmasker (http://repeatmasker.org) resulting in a 27 contig assembly. Gaps were closed using additional sequencing by PCR-based techniques. Quality improvement of the genome sequence was performed using standard PCR to ensure correct assembly and the resolution of any remaining base-conflicts. Assembly validation was confirmed by pulsed-field gel electrophoresis (see above). The nucleotide sequence of the *M. ruminantium* M1 chromosome has been deposited in Genbank under accession number CP001719.

### Genome Analysis and Annotation

A GAMOLA [59]/Artemis [60] software suite was used to manage genome annotation. Protein-encoding open reading frames (ORFs) were identified using the ORF-prediction program Glimmer [61] and BLASTX [62]. A manual inspection was performed to verify or, if necessary, redefine the start and stop of each ORF. Assignment of protein function to ORFs was performed manually using results from the following sources; BLASTP [63] to both a non-redundant protein database provided by the National Centre for Biotechnology Information (NCBI) [64] and clusters of orthologous groups (COG) database [65]. HMMER [66] was used to identify protein motifs to both the PFAM [67] and TIGRFAM [68] libraries. TMHMM [69] (http://www.cbs.dtu.dk/services/TMHMM/) was used to predict transmembrane sequences, and SignalP [70] was used for the prediction of signal peptides. Ribosomal RNA genes were detected on the basis of BLASTN searches to a custom GAMOLA ribosomal database. Transfer RNA genes were identified using tRNAscan-SE [71]. Miscellaneous-coding RNAs were identified using the Rfam database [72] utilizing the INFERNAL software package [73]. Insertion sequence elements were identified using Repeatfinder [74] and BLAST and annotated manually. Genome atlas visualisations were constructed using GENEWIZ [75]. Horizontal gene transfer studies were performed using Darkhorse [76], GC% content [77] and the Codon Adaptation Index [78]. A BLAST analysis was performed against the arCOG [79] database. Analysis of non-ribosomal peptide synthetases (NRPSs) was performed using NRPSpredictor [80]. An LPxTG-HMM [35] was used for the identification of LPxTG motifs. Metabolic pathway reconstructions were performed using Pathway Voyager [81] and the KEGG (Kyoto Encyclopedia of Genes and Genomes) database [82] combined with an extensive review of the literature. Genome sequence was prepared for National Center for Biotechnology Information (NCBI) submission using Sequin [83].The adenine residue of the start codon of the Cdc6-1(mru0001) gene was chosen as the first base for the M1 genome. For GC skew and synteny analysis, the sequences of genomes of other members of the order Methanobacteriales were rotated to begin at the same location. GC skew analysis was performed by circular_diagram.pl (Rutherford, K, Sanger Centre software) and synteny plots were generated using MUMmer3.0 [84].

### Vaccine Target ORF Identification

To identify the surface-exposed or membrane-associated ORFs of M1 a combination of methods was utilized. To date, there is no signal peptide model for archaea. There are simply too few experimentally verified secretory proteins available for Archaea to train a specific model. For this reason ORF sequences were analysed for the presence of signal peptides using SignalP Version 3.0 [70] trained against the Gram-positive, Gram-negative and Eukaryotic models and the results combined. SignalP-HMM (hidden markov models) was used to discriminate between signal peptide and non-signal peptide ORFs whereas SignalP-NN (neural networks) was utilized for the prediction of cleavage sites as described by Emanuelsson *et al.* [85]. TMHMM [69] (http://www.cbs.dtu.dk/services/TMHMM/) was used for the prediction of transmembrane domains and PSORT [86] trained on a Gram-positive model was used to predict a protein's subcellular localization. BLASTP results were analyzed to identify methanogen specific ORFs.

### Chemogenomics Target ORF Identification

#### Metabolic profiling analyses

Several factors were taken into consideration when performing this analysis. Utilizing the metabolic reconstruction of M1 and an extensive review of the literature, archaeal- or methanogen-specific enzymes, or enzymes with sufficient structural or biochemical differences compared to their bacterial or eukaryl counterparts were identified. Some methanogen enzymes or pathways that have been previously targeted by researchers for inhibition demonstrating the essentiality of certain enzymes/pathways were also taken into consideration. In addition, a few enzymes which represent key enzymes to several pathways or are well known validated targets in pathogenic bacteria or parasites, whilst still retaining sufficient sequence divergence to potentially be able to be targeted effectively were also included. Most of the cell wall enzymes are listed as the majority of successful antibiotics that have been developed against pathogenic bacteria target cell wall biosynthesis. Methanobacterial cell wall synthesis, despite apparently sharing some common enzymes (e.g. mur ligases) is widely divergent in biochemical terms from bacterial cell wall synthesis and the homologous enzymes share only limited sequence homology. Unfortunately, the degree to which strain M1, or other rumen methanogens, are able to utilize amino acids, vitamins, or purine or pyrimidine compounds in rumen fluid is still unknown, and thus the targeting of these pathways would carry some risks.

#### Functional genome distribution (FGD)

A FGD analysis (Altermann 2009, manuscript in preparation) was performed using 26 publicly available draft and complete methanogen genome sequences (dbMethano, Table S7). In contrast to an evolutionary phylogeny, FGD analyzes the functional relationship between microbes based on their predicted ORFeomes. FGD is a comparative genomics approach to genome-genome comparisons, emphasizing functional relationships rather than ancestral lineages. Briefly, pooled ORFeomes are subjected to all-vs-all analyses, evaluating the level and quality of amino-acid similarities between individual ORFs pairings. Individual results for each genome-genome combination are then combined into a symmetrical distance matrix and can be visualized using the Unweighted Pair Group Method with Arithmetic mean (UPGMA) method [87]. Strain and cluster conserved and specific gene sets were mined based on respective BLAST e-values, using custom developed software.

#### Differential blast analysis (DBA)

The reference genome of M1 was subjected to analysis against two BLASTP databases using GAMOLA [59]. The first amino-acid database employed all methanogen ORFeomes used in the FGD analysis (dbMethano), while the second database was comprised of the non-redundant database (nr) as provided by NCBI, excluding hits to genera used in dbMethano. E-values of best BLASTP hits for both database sets were consolidated into an empirically determined e-value trust level range ([T_e-value_]) and their respective differential calculated as follows: Δ = (T_nr_ – T_dbMethano_). Results were visualized on a genome atlas using Genewiz and software developed in-house.

### Peptide Vaccine Methods

The use of synthetic peptides to raise antibodies against predicted M1 surface proteins was investigated. The M1 proteins encoding the membrane-spanning subunits of tetrahydromethanopterin S-methyltransferase (MtrCDE, mru1921, 1922 and 1923), adhesin-like proteins (mru2049, 0842, 0143 and 2048) and a magnesium chelatase subunit H (BchH, mru2047) containing N-terminal and C-terminal TMHs, were analysed to identify extracellular peptide sequences which might serve as potential antibody binding sites. Nine suitable peptide sequences from extracellular regions of these eight proteins were identified and used to guide the manufacture of the corresponding synthetic peptides. Each peptide was coupled to the Keyhole Limpet hemocyanin (KLH) protein via an additional N- or C-terminal cysteine residue and a maleimidocaproyl-N-hydroxysuccinimide linker and used to raise antibodies in sheep (Invitrogen, USA). The conjugated peptides (200 µg) were injected intradermally (ID) into sheep (1–3 yr age) in Complete Freund's Adjuvant (CFA) at 10–15 sites on day 0, and secondary boosters in CFA were given on day 14. Six ID injections of 200 µg KLH-peptide in Incomplete Freund's Adjuvant at 10–15 sites were given at days 28, 56, 70, 84, 98 and 112. Test bleeds (2–5 ml) were taken on days 42, 56, 84, and 112 for ELISA analyses. Antibody titer was determined with an ELISA with Peptide-GGG (goat gamma globulin) bound in solid phase (0.1 µg/100 µl/well) on high binding 96 well plates. The serum was first diluted 50-fold and then further diluted in 2-fold serial dilutions. The ELISA titer is the estimated dilution factor that resulted in an OD_405_nm of 0.2 and is derived from nonlinear regression analysis of the serial dilution curve. Detection was obtained using an HRP (horseradish peroxidase)-conjugated secondary antibody and ABTS (2,2′-azino-bis(3-ethylbenzthiazoline-6-sulphonic acid). In the antibody-binding experiment M1 cells (40 µl of cells in 2 ml of sodium carbonate buffer) were immobilised on Maxisorp ELISA plates and antibody binding was determined by ELISA. Serum samples were diluted 1/20 in diluent (1% w/v casein in PBS Tween 20 (g/l NaCl, 8.0; KCl, 0.2; Na2HPO4, 1.15; KH2PO4, 0.2; pH 7.2–7.4; Tween 20, 0.5 ml) and incubated at room temperature for 1 hr. Plates were washed 6 times with PBS Tween 20 and conjugate (donkey anti-sheep/goat IgG HRP, 50 µl/well of a 1/5000 diluted solution) and substrate (3, 3′,5, 5′ tetramethyl benzidine, 50 µl/well) were added. After incubation at room temperature in the dark for 15 min, stop solution (50 µl/well of 0.05 M H_2_SO_4_) was added and OD_450_ nm readings were taken.

## Supporting Information

Table S1 Manual functional annotation of the Methanobrevibacter ruminantium M1 predicted open reading frames.
(0.14 MB DOC)

Table S2 HGT analysis.
(0.46 MB DOC)

Table S3 Selection of upregulated genes of the M1 genome when grown in co-culture with Butyrivibrio proteoclasticus B316.
(0.04 MB DOC)

Table S4 Predicted cell surface associated adhesin-like proteins in M1.
(1.00 MB RTF)

Table S5 Potential chemogenomic gene targets of the M1 genome based on in-depth literature and metabolic analyses.
(0.17 MB DOC)

Table S6 Summary of the functional genome distribution analysis.
(0.06 MB DOC)

Table S7 Genome sequences used in this study.
(0.04 MB DOC)

Figure S1 (A) PFGE of genomic DNA from M1. Lane 1, λ ladder (New England Biolabs); Lane 2, ApaI/BssHII double digest; Lane 3, ApaI digest; Lane 4, MluI digest; Lane 5, Sizes of MluI fragments. The bands in the λ ladder are multiples of 48.5 kb. (B) In silico restriction map of the M1 chromosome showing the position and fragment size of the Mlu1 digest.
(0.98 MB TIF)

Figure S2 Distribution of genes in the predicted ORFeomes of members of the Methanobacteriales. ORFs are classified according to functional categories in the archaeal COG database [S1].
(0.36 MB TIF)

Figure S3 GC analysis. Base-pair scale (outer circle), G+C content (middle circle) and GC skew (inner circle, (G-C/G+C), green indicates values >1, purple <1). Genomes of members of the Methanobacteriales display a DNA skew similar to bacterial chromosomes. In M1, the origin of replication (oriC) was identified as being immediately upstream of the cdc6-1 gene (mru0001), based on GC skew analysis and homology to the origin of replication experimentally verified for M. thermoautotrophicus [S2]. As with genomes from related methanogens, M1 contains a second cdc6-2 homolog (mru0423). It also contains a truncated third cdc6-3 homolog (mru0259) within the prophage sequence.
(0.93 MB TIF)

Figure S4 Synteny analysis. PROmer [S3] alignment of the genome of M1 against genomes from members of the Methanobacteriales. Whenever the two sequences agree, a coloured line or dot is plotted. The forward matches are displayed in red, while the reverse matches are plotted in blue. If the two sequences were perfectly identical, a single red line would go from the bottom left to the top right. An X-shape pattern is visible is all three synteny plots. It has been proposed that the X-pattern is generated by symmetric chromosomal inversions around the origin of replication [S4]. Units displayed in base-pairs.
(0.49 MB TIF)

Figure S5 Blast Heat Map depicting BLAST-result distribution across the M1 ORFeome. In both figures, the Y-axis (vertical axis) shows all genera with at least 500 and 250 BLAST hits throughout the ORFeome, respectively. Genera are phylogenetically sorted based on a semi-dynamically re-parsed phylogenetic tree obtained from the Ribosomal Database Project II (RDP II) (http://rdp.cme.msu.edu/hierarchy/hierarchy_browser.jsp), selecting NCBI taxonomy, level 10 genera display list and set to include archaeal sequences. Bacterial or archaeal genera not covered within the RDPII data were entered and parsed from a separate data file, where appropriate. Phylogenetic distribution and grouping of genera is indicated using an ASCII based tree-abstraction. The X-axis indicates e-value ranges, and the Z-axis (colour coded) represents the frequency of hits for each genus in each e-value range in log-scale. Respective Log-colour-scales are indicated in each figure, whereby warmer colours indicate higher frequencies. Figure part (A) allows all BLAST hits per genus per ORF, accepting multiple genus hits per ORF. Figure part (B) employs a frequency cutoff of one hit per genus per ORF, effectively limiting the hit rate to the best BLAST hit found in each given ORF and genus.
(1.89 MB TIF)

Figure S6 Proposed biosynthetic pathway for pseudomurein in M1 [S63, S266]. The disaccharide backbone of M1 pseudomurein consists of N-acetylgalactosamine (GalNAc) and N-acetyltalosaminuronic acid (TalNAc) in a β(1–3) linkage. TalNac has not been detected as a monomer and it is believed to be formed during the synthesis of the disaccharide probably by epimerization and oxidation of UDP-GalNAc (Step 1). Synthesis of the pentapeptide involved in crosslinking is believed to start with UDP-glutamic acid followed by stepwise addition of L-amino acids (Step 2). The amino acids found in the pentapeptide are usually alanine, lysine (Lys) and glutamic acid (Glu), but M1 is reported to contain threonine (Thr) instead of alanine [S267]. The UDP activated pentapetide is linked to the disaccharide to give a UDP-disaccharide pentapeptide (Step 3) which is subsequently translocated to the membrane via covalent bond formation with a membrane embedded undecaprenyl phosphate (Step 4). Following their intracellular biosynthesis the pseudomurein repeating units must be exported and assembled. Homologues of the Escherichia coli peptidoglycan lipid II flippase (MurJ) have been reported for pseudomurein producing methanogens [S65] (Step 5), but there are no genes similar to the penicillin binding proteins that carry out the transglycosylation (Step 6) and transpeptidation reactions in bacterial peptidoglycan assembly. Peptide crosslinking of pseudomurein requires removal of a terminal residue of one peptide and linkage from a glutamic acid to the lysine of an adjacent peptide (Step 7), and is probably carried out by transglutaminases. None of the enzymes involved in pseudomurein biosynthesis have been characterized, but analysis of the genome sequence has suggested candidates to carry out several of the steps. Several of these have homologues only in those methanogens with pseudomurein-containing cell walls. Two other transmembrane proteins of unknown function (mru1585 and mru1635) are also only found in pseudomurein-containing species.
(0.50 MB TIF)

Figure S7 M1 M1-C domain. (A) Consensus sequence of forty-four C-terminal regions (200 amino acids) from adhesin-like proteins of M1 (M1-C). (B) LogoBar [S268] display of this consensus. In both figures the region of homology to Big_1 domain (PF02369) is highlighted in grey.
(1.27 MB TIF)

Figure S8 ClustalW [S269] alignment of non-ribosomal peptide synthetases from M1 (mru0068) and Syntrophomonas wolfei subsp. wolfei str. Goettingen (swol1094). Alignment was visualized using Jalview [S270]. Conserved residues are shown in blue. NRPS domain organisation of M1 is displayed via coloured boxes (light blue -condensation domain; green - adenylation domain; orange - phosphopantetheine attachment site; purple - thioester reductase domain).
(2.50 MB TIF)

Figure S9 Functional Genome Distribution of 26 methanogen genomes. Publicly available complete genomes were downloaded in GenBank format where possible. Publicly available draft phase genomes were downloaded in FASTA format, concatenated using a universal spacer-stop-spacer sequence (TTAGTTAGTTAG) and automatically annotated using GAMOLA. Predicted ORFeomes of all genomes were subjected to an FGD analysis and the resulting distance matrix was imported into MEGA4 [S6]. The functional distribution was visualized using the UPGMA method [S7]. The optimal tree with the sum of branch length  = 49.7 is shown. The tree is drawn to scale, with branch lengths in the same units as those of the functional distances used to infer the distribution tree. Accession numbers for individual genomes can be found in Table S7.
(0.39 MB TIF)

References S1 (0.18 MB DOC)

## References

[1] IPCC (Intergovernmental Panel on Climate change) (2007). Climate Change 2007: The Physical Science Basis, Contribution of Working Group I to the Fourth Assessment Report of the Intergovernmental Panel on Climate Change.. http://www.ipcc.ch/publications_and_data/publications_ipcc_fourth_assessment_report_wg1_report_the_physical_science_basis.htm.

[2] Scheehle EA, Kruger D (2006). Global anthropogenic methane and nitrous oxide emissions.. The Energy Journal, Special Issue.

[3] Steinfeld H, Gerber P, Wassenaar T, Castel V, Rosales M (2006). Livestock's Long Shadow: Environmental Issues and Options. Rome: Food and Agriculture Organization of the United Nations.. http://www.fao.org/docrep/010/a0701e/a0701e00.HTM.

[4] Food and Agriculture Organization of the United Nations (FAO) (2008). The State of Food Insecurity in the World.. http://www.fao.org/docrep/011/i0291e/i0291e00.htm.

[5] United Nations (1997). Kyoto Protocol to the United Nations Framework Convention on Climate Change. UNEP.IUC/99/10. Chatlelaine, Switzerland, United Nations Environment Programme's Information Unit for Conventions, for the Climate Change Secretariat.. http://unfccc.int/resource/docs/convkp/kpeng.pdf.

[6] Johnson KA, Johnson DE (1995). Methane emissions from cattle.. J Anim Sci.

[7] Statistics New Zealand (2008). Global New Zealand International Trade, Investment and Travel Profile. New Zealand Ministry of Foreign Affairs & Trade.. http://www.stats.govt.nz/publications/businessindicators/global-nz-dec-08.aspx.

[8] Leslie M, Aspin M, Clark H (2008). Greenhouse gas emissions from New Zealand agriculture: issues, perspectives and industry response.. Aust J Exp Agric.

[9] (2007). Ministry for the Environment. New Zealand's Greenhouse Gas Inventory 1990-2005. http://www.mfe.govt.nz/publications/climate/nir-jul07/html/index.html.

[10] Janssen PH, Kirs M (2008). Structure of the archaeal community of the rumen.. Appl Environ Microbiol.

[11] McAllister TA, Newbold CJ (2008). Redirecting rumen fermentation to reduce methanogenesis.. Aust J Exp Agric.

[12] Rappuoli R (2001). Reverse vaccinology, a genome-based approach to vaccine development.. Vaccine.

[13] Caron PR, Mullican MD, Mashal RD, Wilson KP, Su MS (2001). Chemogenomic approaches to drug discovery.. Curr Opin Chem Biol.

[14] Lobry JR (1996). Asymmetric substitution patterns in the two DNA strands of bacteria.. Mol Biol Evol.

[15] Thauer RK, Kaster AK, Seedorf H, Buckel W, Hedderich R (2008). Methanogenic archaea: ecologically relevant differences in energy conservation.. Nat Rev Microbiol.

[16] Smith PH, Hungate RE (1958). Isolation and characterization of *Methanobacterium ruminantium* n. sp.. J Bacteriol.

[17] Abken HJ, Tietze M, Brodersen J, Bäumer S, Beifuss U (1998). Isolation and characterization of methanophenazine and function of phenazines in membrane-bound electron transport of *Methanosarcina mazei* Gö1.. J Bacteriol.

[18] Berk H, Thauer RK (1997). Function of coenzyme F_420_-dependent NADP reductase in methanogenic archaea containing an NADP-dependent alcohol dehydrogenase.. Arch Microbiol.

[19] Tokura M, Tajima K, Ushida K (1999). Isolation of *Methanobrevibacter* sp. as a ciliate-associated ruminal methanogen.. J Gen Appl Microbiol.

[20] Fricke WF, Seedorf H, Henne A, Krüer M, Liesegang H (2006). The genome sequence of *Methanosphaera stadtmanae* reveals why this human intestinal archaeon is restricted to methanol and H_2_ for methane formation and ATP synthesis.. J Bacteriol.

[21] Reeve JN, Nolling J, Morgan RM, Smith DR (1997). Methanogenesis: genes, genomes, and who's on first?. J Bacteriol.

[22] Moon CD, Pacheco DM, Kelly WJ, Leahy SC, Li D (2008). Reclassification of *Clostridium proteoclasticum* as *Butyrivibrio proteoclasticus* comb. nov., a butyrate-producing ruminal bacterium.. Int J Syst Evol Microbiol.

[23] Bryant MP, Tzeng SF, Robinson IM, Joyner AE (1971). Nutritional requirements of methanogenic bacteria. In F.G. Pohland (ed.), Anaerobic biological treatment processes..

[24] Robinson IM, Allison MJ (1969). Isoleucine biosynthesis from 2-methylbutyric acid by anaerobic bacteria from the rumen.. J Bacteriol.

[25] Graham DE, Xu H, White RH (2002). Identification of coenzyme M biosynthetic phosphosulfolactate synthase: a new family of sulfonate-biosynthesizing enzymes.. J Biol Chem.

[26] König H, Hartmann E, Kärcher U (1994). Pathways and principles of the biosynthesis of methanobacterial cell wall polymers.. Syst Appl Microbiol.

[27] Kandler O, König H (1998). Cell wall polymers in Archaea (Archaebacteria).. Cell Mol Life Sci.

[28] Kandler O, König H (1978). Chemical composition of the peptidoglycan-free cell walls of methanogenic bacteria.. Arch Microbiol.

[29] Kandler O, König H (1985).

[30] Bhavsar AP, Brown ED (2006). Cell wall assembly in *Bacillus subtilis*: how spirals and spaces challenge paradigms.. Mol Microbiol.

[31] Weidenmaier C, Peschel A (2008). Teichoic acids and related cell-wall glycopolymers in Gram-positive physiology and host interactions.. Nat Rev Microbiol.

[32] Yurist-Doutsch S, Chaban B, VanDyke DJ, Jarrell KF, Eichler J (2008). Sweet to the extreme: protein glycosylation in Archaea.. Mol Microbiol.

[33] Wedlock DN, Pedersen G, Denis M, Dey D, Janssen PH (2009). Development of a vaccine to mitigate greenhouse gas emissions in agriculture: Vaccination of sheep with methanogen fractions induces antibodies that block methane production *in vitro*.. NZ Vet J. In press.

[34] Samuel BS, Hansen EE, Manchester JK, Coutinho PM, Henrissat B (2007). Genomic and metabolic adaptations of *Methanobrevibacter smithii* to the human gut.. Proc Natl Acad Sci USA.

[35] Boekhorst J, de Been MW, Kleerebezem M, Siezen RJ (2005). Genome-wide detection and analysis of cell wall-bound proteins with LPxTG-like sorting motifs.. J Bacteriol.

[36] Maresso AW, Schneewind O (2008). Sortase as a target of anti-infective therapy.. Pharmacol Rev.

[37] Klieve AV, Hegarty RS (1999). Opportunities for biological contol of ruminal methanogenesis.. Aust J Exp Agric.

[38] Attwood GT, Kelly WJ, Altermann EH, Leahy SC (2008). Analysis of the *Methanobrevibacter ruminantium* draft genome: understanding methanogen biology to inhibit their action in the rumen.. Aust J Exp Agric.

[39] Hermoso JA, García JL, García P (2007). Taking aim on bacterial pathogens: from phage therapy to enzybiotics.. Curr Opin Microbiol.

[40] Altermann E, Attwood GT, Leahy SC, Kelly WJ, Ronimus RS (2009). Phage ϕ-mru polynucleotides and polypeptides and uses thereof. International application no. PCT/NZ2008/000248.. http://www.wipo.int/pctdb/en/wo.jsp?WO=2009041831.

[41] Amoutzias GD, Van de Peer Y, Mossialos D (2008). Evolution and taxonomic distribution of nonribosomal peptide and polyketide synthases.. Future Microbiol.

[42] Fischbach MA, Walsh CT (2006). Assembly-line enzymology for polyketide and nonribosomal peptide antibiotics: logic, machinery and mechanisms.. Chem Rev.

[43] McInerney MJ, Bryant MP, Pfennig N (1979). Anaerobic bacterium that degrades fatty acids in syntrophic association with methanogens.. Arch Microbiol.

[44] Vivona S, Gardy JL, Ranachandran S, Brinkman FSL, Raghava GPS (2008). Computer-aided biotechnology: from immune-informatics to reverse vaccinology.. Trends Biotechnol.

[45] Lienard T, Becher B, Marschall M, Bowien S, Gottschalk G (1996). Sodium ion translocation by N^5^-methyltetrahydromethanopterin:coenzyme M methyltransferase from *Methanosarcina mazei* Gö1 reconstituted in ether lipid liposomes.. Eur J Biochem.

[46] Balch WE, Fox CE, Magrum LJ, Woese CR, Wolfe RS (1979). Methanogens: reevaluation of a unique biological group.. Microbiol Rev.

[47] Bryant MP, Dougherty RW, Allen RS, Burroughs W, Jaskson NL, McGillard (1965). Rumen methanogenic bacteria.. Physiology of digestion of the ruminant.

[48] Joblin KN, Naylor GE, Williams AG (1990). Effect of *Methanobrevibacter smithii* on xylanolytic activity of anaerobic ruminal fungi.. Appl Environ Microbiol.

[49] Asanuma N, Ishiwata M, Yoshii T, Kikuchi M, Nishina Y (2005). Characterization and transcription of the genes involved in butyrate production in *Butyrivibrio fibrisolvens* type I and type II strains.. Curr Microbiol.

[50] Reymond N, Charles H, Duret L, Calevro F, Beslon G (2004). ROSO: optimizing oligonucleotides probes for microarrays.. Bioinformatics.

[51] Smyth G K (2005). Limma: linear models for microarray data..

[52] Balch WE, Wolfe RS (1976). New approach to cultivation of methanogenic bacteria: 2-mercaptoethanesulfonic acid (HS-CoM)-dependent growth of *Methanobacterium ruminantium* in a pressurized atmosphere.. Appl Environ Microbiol.

[53] Widdel F, Kohring GW, Mayer F (1983). Studies on dissimilatory sulfate-reducing bacteria that decompose fatty acids. III. Characterization of the filamentous gliding *Desulfonema limicola* gen. *nov*. sp. *nov*., and *Desulfonema magnum* sp. *nov*.. Arch Microbiol.

[54] Tschech A, Pfennig N (1984). Growth yield increase linked to caffeate reduction in *Acetobacterium woodii*.. Arch Microbiol.

[55] Jarrell KF, Faguy D, Herbert AM, Kalmakoff ML (1992). A general method of isolating high molecular weight DNA from methanogenic archaea (archaebacteria). Can J Microbiol.

[56] Goldberg SM, Johnson J, Busam D, Feldbylum T, Ferriera S A Sanger/pyrosequencing hybrid approach for the generation of high-quality draft assemblies of marine microbial genomes.. Proc Natl Acad Sci USA.

[57] Staden R, Beal KF, Bonfield JK (2000). The Staden package, 1998.. Methods Mol Biol.

[58] Ewing B, Hillier L, Wendl MC, Green P (1998). Base-calling of automated sequencer traces using phred. I. Accuracy assessment.. Genome Res.

[59] Altermann E, Klaenhammer TR (2003). GAMOLA: a new local solution for sequence annotation and analyzing draft and finished prokaryotic genomes.. Omics.

[60] Rutherford K, Parkhill J, Crook J, Horsnell T, Rice P Artemis: sequence visualization and annotation.. Bioinformatics.

[61] Delcher AL, Harmon D, Kasif S, White O, Salzberg SL (1999). Improved microbial gene identification with GLIMMER.. Nucleic Acids Res.

[62] Gish W, States DJ (1993). Identification of protein coding regions by database similarity search.. Nat Gen.

[63] Altschul SF, Gish W, Miller W, Myers EW, Lipman DJ (1990). Basic local alignment search tool.. J Mol Biol.

[64] Sayers EW, Barrett T, Benson DA, Bryant SH, Canese K (2009). Database resources of the National Center for Biotechnology Information.. Nucleic Acids Res.

[65] Tatusov RL, Galperin MY, Natale DA, Koonin EV (2000). The COG database: a tool for genome-scale analysis of protein functions and evolution.. Nucleic Acids Res.

[66] Eddy SR (1998). Profile hidden Markov models.. Bioinformatics.

[67] Finn RD, Tate J, Mistry J, Coggill PC, Sammut SJ (2008). The Pfam protein families database.. Nucleic Acids Res.

[68] Haft DH, Selengut JD, White O (2003). The TIGRFAMs database of protein families.. Nucleic Acids Res.

[69] Krogh A, Larsson B, von Heijne G, Sonnhammer EL (2001). Predicting transmembrane protein topology with a hidden Markov model: application to complete genomes.. J Mol Biol.

[70] Bendtsen JD, Nielsen H, von Heijne G, Brunak S (2004). Improved prediction of signal peptides: SignalP 3.0.. J Mol Biol.

[71] Lowe TM, Eddy SR (1997). tRNAscan-SE: a program for improved detection of transfer RNA genes in genomic sequence.. Nucleic Acids Res.

[72] Gardner PP, Daub J, Tate JG, Nawrocki EP, Kolbe DL (2009). Rfam: updates to the RNA families database.. Nucleic Acids Res.

[73] Eddy SR (2002). A memory-efficient dynamic programming algorithm for optimal alignment of a sequence to an RNA secondary structure.. BMC Bioinformatics.

[74] Volfovsky N, Haas BJ, Salzberg SL (2001). A clustering method for repeat analysis in DNA sequences.. Genome Biol.

[75] Jensen LJ, Friis C, Ussery DW (1999). Three views of microbial genomes.. Res Microbiol.

[76] Podell S, Gaasterland T (2007). Darkhorse: a method for genome-wide prediction of horizontal gene transfer.. Genome Biol.

[77] Rice P, Longden I, Bleasby A (2000). EMBOSS: The European Molecular Biology Open Software Suite.. Trends Genet.

[78] Sharp PM, Li WH (1987). The codon Adaptation Index—a measure of directional synonymous codon usage bias, and its potential applications.. Nucleic Acids Res.

[79] Makarova KS, Sorokin AV, Novichkov PS, Wolf YI, Koonin EV (2007). Clusters of orthologous genes for 41 archaeal genomes and implications for evolutionary genomics of archaea.. Biol Direct.

[80] Rausch C, Weber T, Kohlbacher O, Wohlleben W, Huson DH (2005). Specificity prediction of adenylation domains in nonribosomal peptide synthetases (NRPS) using Transductive Support Vector Machines (TSVM).. Nucleic Acids Res.

[81] Altermann E, Klaenhammer TR (2005). PathwayVoyager: pathway mapping using the Kyoto Encyclopedia of Genes and Genomes (KEGG) database.. BMC Genomics.

[82] Kanehisa M, Goto S (2000). KEGG: Kyoto Encyclopedia of Genes and Genomes.. Nucleic Acids Res.

[83] Benson DA, Karsch-Mizrachi I, Lipman DJ, Ostell J, Sayers EW (2009). Genbank.. Nucleic Acids Res.

[84] Delcher AL, Salzberg SL, Phillippy AM (2003). Using MUMmer to identify similar regions in large sequence sets..

[85] Emanuelsson O, Brunak S, von Heijne G, Nielsen H (2007). Locating proteins in the cell using TargetP, SignalP and related tools.. Nat Protoc.

[86] Nakai K, Horton P (1999). PSORT: a program for detecting the sorting signals of proteins and predicting their subcellular localization.. Trends Biochem Sci.

[87] Sneath PHA, Sokal RR (1973). *Numerical Taxonomy. Freeman*, San Francisco..

[88] Zeikus JG, Bowen BG (1975). Comparative ultrastructure of methanogenic bacteria.. Can J Microbiol.

[89] Miller TL (2001).

[90] Graham LL, Beveridge TJ (1994). Structural differentiation of the *Bacillus subtilis* 168 cell wall.. J Bacteriol.

[91] Smith DR, Doucette-Stamm LA, Deloughery C, Lee H, Dubois J (1997). Complete genome sequence of *Methanobacterium thermoautotrophicum* ΔH: functional analysis and comparative genomics.. J Bacteriol.

