# Supplementary material for: The Genome Sequence of the Rumen Methanogen Methanobrevibacter ruminantium Reveals New Possibilities for Controlling Ruminant Methane Emissions
Source: PLoS One. 2010 Jan 28;5(1):e8926. doi: 10.1371/journal.pone.0008926 (PMC2812497; doi:10.1371/journal.pone.0008926)
Supplement: Table S1 — Manual functional annotation of the Methanobrevibacter ruminantium M1 predicted open reading frames. (0.14 MB DOC) [file pone.0008926.s001.doc]

**Table S1.** Manual functional annotation of the *Methanobrevibacter ruminantium* M1 predicted open reading frames. Table excludes hypothetical proteins.

*************************************

**AMINO ACID METABOLISM**

**Arginine**

mru1029 acetylglutamate kinase ArgB

mru0149 acetylornithine aminotransferase ArgD

mru1476 argininosuccinate lyase ArgH

mru2017 argininosuccinate synthase ArgG

mru1023 bifunctional ornithine acetyltransferase/N-acetylglutamate synthase protein ArgJ

mru1719 N-acetyl-gamma-glutamyl-phosphate reductase ArgC

mru2115 ornithine carbamoyltransferase ArgF

**Aspartate/asparagine**

mru1143 asparagine synthase (glutamine-hydrolyzing) AsnB

**Chorismate**

mru1089 3-dehydroquinate dehydratase type I AroD

mru0998 3-dehydroquinate synthase AroB

mru1577 3-phosphoshikimate 1-carboxyvinyltransferase AroA

mru1561 chorismate synthase AroC

mru1244 shikimate 5-dehydrogenase AroE

mru1676 shikimate kinase AroK

**Cysteine**

mru1574 cysteine synthase CysKM1

mru2096 cysteine synthase CysKM2

mru1573 serine O-acetyltransferase CysE

**General**

mru0506 aspartate aminotransferase

**Glutamate/glutamine**

mru1761 glutamate dehydrogenase GdhA

mru2082 glutamate synthase alpha subunit GltA

mru2080 glutamate synthase beta subunit GltB

mru0810 glutamate synthase domain-containing protein

mru2079 glutamine amidotransferase

mru0350 glutamine synthetase GlnA1

mru2078 glutamine synthetase GlnA2

mru0349 transcriptional repressor of nif and glnA operons NrpR

**Glycine**

mru0122 serine hydroxymethyltransferase GlyA

**Histidine**

mru0010 ATP phosphoribosyltransferase HisG1

mru1050 ATP phosphoribosyltransferase HisG2

mru2139 bifunctional imidazoleglycerol-phosphate dehydratase HisB

mru1337 hisA/hisF family protein HisAF

mru1015 histidinol dehydrogenase HisD

mru0454 histidinol-phosphate aminotransferase HisC

mru0182 imidazole glycerol phosphate synthase glutamine amidotransferase subunit HisH

mru0135 imidazoleglycerol-phosphate synthase cyclase subunit HisF

mru1249 phosphoribosyl-AMP cyclohydrolase HisI

mru2031 phosphoribosyl-ATP pyrophosphohydrolase HisE

mru1717 phosphoribosylformimino-5- aminoimidazole carboxamide ribotide isomerase HisA

**Homoserine**

mru1141 allosteric regulator of homoserine dehydrogenase

mru1140 homoserine dehydrogenase Hom

**Lysine**

mru1672 aspartate kinase Ask

mru1669 aspartate-semialdehyde dehydrogenase Asd

mru0941 diaminopimelate aminotransferase DapL

mru0152 diaminopimelate decarboxylase LysA

mru0153 diaminopimelate epimerase DapF

mru1670 dihydrodipicolinate reductase DapB

mru1671 dihydrodipicolinate synthase DapA

**Methionine**

mru0611 homoserine O-acetyltransferase MetX1

mru1205 homoserine o-acetyltransferase MetX2

mru1620 methionine synthase MetE

mru1148 O-acetylhomoserine/O-acetylserine sulfhydrylase MetZ/CysK1

mru1569 O-acetylhomoserine/O-acetylserine sulfhydrylase MetZ/CysK2

**Phenylalanine/tyrosine**

mru1674 chorismate mutase AroH

mru1992 prephenate dehydratase PheA

mru0468 prephenate dehydrogenase TyrA1

mru1975 prephenate dehydrogenase TyrA2

**Polyamines**

mru1741 arginase/agmatinase family protein

mru0603 N-carbamoyl-D-amino acid amidohydrolase AguB

mru1743 pyruvoyl-dependent arginine decarboxylase PdaD

**Proline**

mru0518 delta 1-pyrroline-5-carboxylate synthetase

mru1509 pyrroline-5-carboxylate reductase ProC

**Serine**

mru0414 aminotransferase class V family

mru0678 phosphoglycerate dehydrogenase SerA

mru0388 phosphoserine phosphatase SerB

**Threonine**

mru1492 threonine synthase ThrC

**Tryptophan**

mru0210 anthranilate phosphoribosyltransferase TrpD

mru0208 anthranilate synthase component I TrpE

mru0209 anthranilate synthase component II TrpG

mru0211 indole-3-glycerol phosphate synthase TrpC

mru0212 phosphoribosylanthranilate isomerase TrpF

mru0214 tryptophan synthase alpha subunit TrpA

mru2159 tryptophan synthase beta subunit TrpB

mru0213 tryptophan synthase beta subunit TrpB1

mru0477 tryptophan-binding repressor TrpY

**Valine/leucine/isoleucine**

mru2155 2-isopropylmalate synthase LeuA

mru0105 3-isopropylmalate dehydratase large subunit LeuC

mru0104 3-isopropylmalate dehydratase small subunit LeuD

mru0103 3-isopropylmalate dehydrogenase LeuB

mru0410 acetolactate synthase large subunit IlvB1

mru2112 acetolactate synthase large subunit IlvB2

mru2111 acetolactate synthase small subunit IlvN

mru2107 branched-chain-amino-acid aminotransferase IlvE

mru1414 citramalate synthase CimA

mru2119 dihydroxy-acid dehydratase IlvD

mru2110 ketol-acid reductoisomerase IlvC

**Salvage- general**

mru0656 indolepyruvate ferredoxin oxidoreductase alpha subunit IorA

mru0657 indolepyruvate ferredoxin oxidoreductase beta subunit IorB

mru1432 ketoisovalerate ferredoxin oxidoreductase alpha subunit VorA

mru1431 ketoisovalerate ferredoxin oxidoreductase beta subunit VorB

mru1433 ketoisovalerate ferredoxin oxidoreductase gamma subunit VorC

**Salvage- methionine**

mru0427 methylthioadenosine phosphorylase MtnP

mru0380 S-adenosyl-L-homocysteine hydrolase AhcY

mru0125 S-adenosylmethionine synthetase MetK

**Salvage- tyrosine**

mru0007 4-hydroxyphenylacetate degradation bifunctional isomerase/decarboxylase HpaG

*************************************

**CELL CYCLE**

**Cell division**

mru2160 cell division ATPase MinD

mru0435 cell division control protein Cdc48

mru0481 cell division protein FtsZ

**Chromosome replication**

mru0445 ATP-dependent DNA ligase DnlI

mru0001 cdc6 family replication initiation protein Cdc6-1

mru0423 cdc6 family replication initiation protein Cdc6-2

mru1983 DNA polymerase family B PolB1

mru1553 DNA polymerase family B PolB2

mru0240 DNA polymerase large subunit DP2 PolD2

mru0173 DNA polymerase sliding clamp subunit PCNA family Pcn

mru2212 DNA polymerase small subunit DP1 PolD1

mru0711 DNA primase DnaG

mru0969 DNA primase large subunit PriB

mru0974 DNA primase small subunit PriA

mru0685 DNA-binding protein

mru0382 flap endonuclease Fen

mru1667 HIRAN domain-containing protein

mru0446 OB fold nucleic acid binding domain-containing protein

mru0114 replication factor A

mru1129 replication factor C large subunit RfcL

mru1130 replication factor C small subunit RfcS

mru0591 replicative DNA helicase Mcm

mru1838 ribonuclease HII RnhB

mru0710 tyrosine recombinase XerC

**Genome segregation**

mru1603 chromosome partitioning ATPase ParA

mru0390 DNA topoisomerase I TopA

mru1864 DNA topoisomerase VI subunit A

mru1865 DNA topoisomerase VI subunit B

mru1158 RecF/RecN/SMC N terminal domain-containing protein

*************************************

**CELL ENVELOPE**

**Cell surface proteins**

mru0004 adhesin-like protein

mru0019 adhesin-like protein

mru0031 adhesin-like protein

mru0032 adhesin-like protein

mru0033 adhesin-like protein

mru0036 adhesin-like protein

mru0038 adhesin-like protein

mru0048 adhesin-like protein

mru0064 adhesin-like protein

mru0072 adhesin-like protein

mru0076 adhesin-like protein

mru0077 adhesin-like protein

mru0079 adhesin-like protein

mru0082 adhesin-like protein

mru0083 adhesin-like protein

mru0084 adhesin-like protein

mru0085 adhesin-like protein

mru0086 adhesin-like protein

mru0090 adhesin-like protein

mru0160 adhesin-like protein

mru0245 adhesin-like protein

mru0255 adhesin-like protein

mru0326 adhesin-like protein

mru0327 adhesin-like protein

mru0331 adhesin-like protein

mru0338 adhesin-like protein

mru0417 adhesin-like protein

mru0418 adhesin-like protein

mru0419 adhesin-like protein

mru0450 adhesin-like protein

mru0451 adhesin-like protein

mru0493 adhesin-like protein

mru0687 adhesin-like protein

mru0704 adhesin-like protein

mru0723 adhesin-like protein

mru0775 adhesin-like protein

mru0811 adhesin-like protein

mru0881 adhesin-like protein

mru0896 adhesin-like protein

mru0962 adhesin-like protein

mru0963 adhesin-like protein

mru0970 adhesin-like protein

mru0977 adhesin-like protein

mru0978 adhesin-like protein

mru0979 adhesin-like protein

mru1076 adhesin-like protein

mru1077 adhesin-like protein

mru1124 adhesin-like protein

mru1210 adhesin-like protein

mru1222 adhesin-like protein

mru1246 adhesin-like protein

mru1247 adhesin-like protein

mru1263 adhesin-like protein

mru1299 adhesin-like protein

mru1312 adhesin-like protein

mru1313 adhesin-like protein

mru1314 adhesin-like protein

mru1315 adhesin-like protein

mru1342 adhesin-like protein

mru1358 adhesin-like protein

mru1376 adhesin-like protein

mru1386 adhesin-like protein

mru1417 adhesin-like protein

mru1424 adhesin-like protein

mru1465 adhesin-like protein

mru1500 adhesin-like protein

mru1506 adhesin-like protein

mru1513 adhesin-like protein

mru1650 adhesin-like protein

mru1651 adhesin-like protein

mru1659 adhesin-like protein

mru1661 adhesin-like protein

mru1726 adhesin-like protein

mru1798 adhesin-like protein

mru1971 adhesin-like protein

mru1996 adhesin-like protein

mru2043 adhesin-like protein

mru2048 adhesin-like protein

mru2049 adhesin-like protein

mru2052 adhesin-like protein

mru2053 adhesin-like protein

mru2054 adhesin-like protein

mru2055 adhesin-like protein

mru2059 adhesin-like protein

mru2090 adhesin-like protein

mru2134 adhesin-like protein

mru2147 adhesin-like protein

mru2178 adhesin-like protein

mru2189 adhesin-like protein

mru0015 adhesin-like protein with cysteine protease domain

mru0020 adhesin-like protein with cysteine protease domain

mru0143 adhesin-like protein with cysteine protease domain

mru0222 adhesin-like protein with cysteine protease domain

mru0727 adhesin-like protein with cysteine protease domain

mru0772 adhesin-like protein with cysteine protease domain

mru0839 adhesin-like protein with cysteine protease domain

mru0842 adhesin-like protein with cysteine protease domain

mru0843 adhesin-like protein with cysteine protease domain

mru1387 adhesin-like protein with cysteine protease domain

mru0824 adhesin-like protein with transglutaminase domain

mru0828 adhesin-like protein with transglutaminase domain

mru1497 adhesin-like protein with transglutaminase domain

mru1499 adhesin-like protein with transglutaminase domain

mru1604 adhesin-like protein with transglutaminase domain

**Expolysaccharide synthesis**

mru1067 acetyltransferase

mru0433 dolichol kinase

mru0109 dTDP-4-dehydrorhamnose 3,5- epimerase RfbC1

mru1061 dTDP-4-dehydrorhamnose 3,5-epimerase RfbC

mru0107 dTDP-4-dehydrorhamnose reductase RfbD

mru0110 dTDP-glucose 4,6-dehydratase RfbB1

mru1060 dTDP-glucose 4,6-dehydratase RfbB2

mru0113 exopolysaccharide biosynthesis polyprenyl glycosylphosphotransferase

mru1062 glucose-1-phosphate thymidylyltransferase RfbA

mru0108 glucose-1-phosphate thymidylyltransferase RfbA1

mru1527 glycosyl transferase

mru1528 glycosyl transferase

mru0101 glycosyl transferase GT2 family

mru0111 glycosyl transferase GT2 family

mru0112 glycosyl transferase GT2 family

mru1049 glycosyl transferase GT2 family

mru1064 glycosyl transferase GT2 family

mru1069 glycosyl transferase GT2 family

mru1074 glycosyl transferase GT2 family

mru1214 glycosyl transferase GT2 family

mru1264 glycosyl transferase GT2 family

mru1458 glycosyl transferase GT2 family

mru1525 glycosyl transferase GT2 family

mru1545 glycosyl transferase GT2 family

mru0099 glycosyl transferase GT4 family

mru1066 glycosyl transferase GT4 family

mru1068 glycosyl transferase GT4 family

mru1378 glycosyl transferase GT4 family

mru1679 glycosyl transferase GT4 family

mru1883 glycosyl transferase GT4 family

mru1072 glycosyl transferase GT2 family

mru1459 NAD dependent epimerase/dehydratase

mru1526 nucleotidyl transferase

mru1522 polysaccharide biosynthesis protein

mru1523 polysaccharide biosynthesis protein

mru1524 polysaccharide biosynthesis protein

mru1071 polysaccharide/polyol phosphate ABC transporter ATP-binding protein

mru1457 polysaccharide/polyol phosphate ABC transporter ATP-binding protein

mru1070 polysaccharide/polyol phosphate ABC transporter permease protein

mru1456 polysaccharide/polyol phosphate ABC transporter permease protein

mru1529 UDP-galactopyranose mutase Glf

mru1461 UDP-glucose pyrophosphorylase GalU

mru1051 UDP-glucose/GDP-mannose dehydrogenase

mru1075 UDP-glucose/GDP-mannose dehydrogenase

mru0106 UDP-N-acetyl-D-mannosaminuronate dehydrogenase

mru1697 UDP-N-acetylglucosamine 2-epimerase

**Other**

mru1836 cell shape determining protein MreB/Mrl family

mru1047 poly-gamma-glutamate biosynthesis protein

**Pseudomurein biosynthesis**

mru2175 cell wall biosynthesis glycosyl transferase

mru0707 cell wall biosynthesis protein Mur ligase family

mru1042 cell wall biosynthesis protein Mur ligase family

mru1118 cell wall biosynthesis protein Mur ligase family

mru1745 cell wall biosynthesis protein Mur ligase family

mru2091 cell wall biosynthesis protein Mur ligase family

mru2092 cell wall biosynthesis protein Mur ligase family

mru0964 cell wall biosynthesis protein phospho-N-acetylmuramoyl-pentapeptide-transferase family

mru1041 cell wall biosynthesis protein phospho-N-acetylmuramoyl-pentapeptide-transferase family

mru2126 cell wall biosynthesis protein UDP-glycosyltransferase family

mru1293 glucosamine-fructose-6-phosphate aminotransferase GlmS1

mru1536 glucosamine-fructose-6-phosphate aminotransferase GlmS2

mru1388 NAD dependent epimerase/dehydratase

mru1413 NAD dependent epimerase/dehydratase

mru0458 phosphoglucosamine mutase GlmM1

mru0449 phosphoglucosamine mutase GlmM2

mru1733 phosphosugar-binding protein

mru2136 polysaccharide biosynthesis protein

mru1470 UDP-glucose 4-epimerase GalE

mru0456 UDP-N-acetylglucosamine diphosphorylase/glucosamine-1-phosphate N-acetyltransferase GlmU

mru1005 undecaprenyl pyrophosphate synthetase UppS

mru2108 undecaprenyl-diphosphatase UppP

**Sialic acid biosynthesis**

mru1876 CMP-N-acetylneuraminic acid synthetase NeuA

mru1878 N-acetyl neuramic acid synthetase NeuB

mru1879 sialyltransferase

mru1880 polysaccharide biosynthesis protein

**Teichoic acid biosynthesis**

mru0715 glycosyl transferase GT2 family

mru1056 2-C-methyl-D-erythritol 4-phosphate cytidylyltransferase

mru1057 alcohol dehydrogenase

mru1058 glycosyl transferase GT2 family

mru1078 glycosyl transferase GT2 family/CDP-glycerol:poly(glycerophosphate) glycerophosphotransferase

mru1079 CDP-glycerol:poly(glycerophosphate) glycerophosphotransferase

mru1718 glycerol-3-phosphate cytidylyltransferase

mru2181 glycosyl transferase GT2 family/CDP-glycerol:poly(glycerophosphate) glycerophosphotransferase

mru2182 glycosyl transferase GT2 family/CDP-glycerol:poly(glycerophosphate) glycerophosphotransferase

mru2183 glycosyl transferase GT2 family

mru2190 nucleotidyl transferase

mru2191 CDP-glycerol:poly(glycerophosphate) glycerophosphotransferase

*************************************

**CELLULAR PROCESSES**

**Oxidative stress response**

mru1564 desulfoferrodoxin Dfx

mru1507 F420H2 oxidase FprA1

mru0131 F420H2 oxidase FprA2

mru1257 ferritin-like domain-containing protein

mru0958 NADH oxidase Nox

mru0457 rubredoxin Rub1

mru1258 rubredoxin Rub2

mru1259 rubredoxin Rub3

mru0735 rubrerythrin Rbr1

mru1367 rubrerythrin Rbr2

**Stress response**

mru1368 bile salt hydrolase

mru0183 protein disulfide-isomerase thioredoxin-related

mru1261 universal stress protein UspA1

mru0440 universal stress protein UspA2

*************************************

**CENTRAL CARBON METABOLISM**

**Acetate**

mru1434 acetyl-CoA synthetase AcsA

mru1570 ADP-dependent acetyl-CoA synthetase Acs

mru0550 pyruvate ferredoxin oxidoreductase alpha subunit PorA

mru0551 pyruvate ferredoxin oxidoreductase beta subunit PorB

mru0549 pyruvate ferredoxin oxidoreductase delta subunit PorD

mru0548 pyruvate ferredoxin oxidoreductase gamma subunit PorC

mru0552 pyruvate ferredoxin oxidoreductase-associated PorE

mru0553 pyruvate ferredoxin oxidoreductase-associated PorF

mru1786 transporter SSS family

**Aromatic compounds**

mru1045 4-oxalocrotonate tautomerase family enzyme DmpI

mru1381 carboxymuconolactone decarboxylase family protein PcaC1

mru1382 carboxymuconolactone decarboxylase family protein PcaC2

**Bicarbonate**

mru0951 bicarbonate ABC transporter ATP-binding protein BtcA

mru0950 bicarbonate ABC transporter permease protein BtcB

mru0949 bicarbonate ABC transporter substrate-binding protein BtcC

mru1602 carbonic anhydrase Cab

**Butanol**

mru0990 3-hydroxybutyryl-CoA dehydrogenase Hbd

**Formate**

mru0453 pyruvate formate-lyase-activating enzyme PflA1

mru1965 pyruvate formate-lyase-activating enzyme PflA2

mru1519 pyruvate-formate lyase Pfl

**Gluconeogenesis**

mru0459 2,3-bisphosphoglycerate-independent phosphoglycerate mutase ApgM1

mru1139 2,3-bisphosphoglycerate-independent phosphoglycerate mutase ApgM2

mru0628 2-phosphoglycerate kinase Pgk2A

mru0822 2-phosphoglycerate kinase Pgk2B

mru1017 cyclic 2,3-diphosphoglycerate-synthetase

mru0498 fructose 1,6-bisphosphatase Fbp

mru1856 glyceraldehyde-3-phosphate dehydrogenase Gap

mru1897 phosphoenolpyruvate synthase PpsA1

mru2083 phosphoenolpyruvate synthase/pyruvate phosphate dikinase

mru1821 phosphoglycerate kinase Pgk

mru0914 phosphopyruvate hydratase Eno

mru0997 phospho-2-dehydro-3-deoxyheptonate aldolase/fructose-bisphosphate aldolase

mru0635 pyruvate kinase PykA

mru1822 triosephosphate isomerase TpiA

**Glycolate salvage pathway**

mru1874 phosphoglycolate phosphatase Gph

**Inositol biosynthesis**

mru1744 bifunctional inositol-1 monophosphatase/fructose-1,6-bisphosphatase/ATP-NAD kinase

mru1887 myo-inositol-1-phosphate synthase

**Propanoate**

mru0688 2-methylcitrate dehydratase PrpD

mru0691 2-methylcitrate synthase/citrate synthase II PrpC/CitZ

**PRPP synthesis**

mru0957 ribose 5-phosphate isomerase A RpiA

mru1634 ribose-phosphate diphosphokinase Prs

**Ribulose monophosphate pathway**

mru0250 3-hexulose-6-phosphate isomerase Phi1

mru1310 3-hexulose-6-phosphate isomerase Phi2

mru2131 bifunctional formaldehyde-activating enzyme/3- hexulose-6-phosphate synthase Fae/Hps

**Tricarboxylic cycle**

mru1828 2-oxoglutarate ferredoxin oxidoreductase subunit alpha KorA

mru1827 2-oxoglutarate ferredoxin oxidoreductase subunit beta KorB

mru1829 2-oxoglutarate ferredoxin oxidoreductase subunit delta KorD

mru1826 2-oxoglutarate ferredoxin oxidoreductase subunit gamma KorC

mru2095 aconitase

mru0556 fumarate hydratase FumA1

mru0690 fumarate hydratase FumA2

mru0841 fumarate hydratase FumA3

mru1895 fumarate hydratase FumA4

mru1255 malate dehydrogenase Mdh

mru0847 pyruvate carboxylase subunit A PycA

mru1888 pyruvate carboxylase subunit B PycB

mru0088 succinate dehydrogenase/fumarate reductase flavoprotein subunit SdhA

mru0655 succinate dehydrogenase/fumarate reductase iron-sulfur protein SdhB

mru1091 succinate-CoA ligase alpha subunit SucD

mru1824 succinyl-CoA synthetase beta subunit SucC

**Other**

mru1685 deoxyribose-phosphate aldolase DeoC

*************************************

**ENERGY METABOLISM**

**Electron transfer**

mru0915 4Fe-4S binding domain-containing protein

mru1345 4Fe-4S binding domain-containing protein

mru1711 4Fe-4S binding domain-containing protein

mru1715 4Fe-4S binding domain-containing protein

mru1716 4Fe-4S binding domain-containing protein

mru2036 4Fe-4S binding domain-containing protein

mru1211 4Fe-4S binding domain-containing protein

mru0188 4Fe-4S ferredoxin binding domain-containing protein

mru0362 4Fe-4S ferredoxin binding domain-containing protein

mru0373 4Fe-4S ferredoxin binding domain-containing protein

mru0123 archaeoflavoprotein AfpA

mru0184 cytochrome C-type biogenesis protein DsbD

mru0544 ferredoxin

mru0653 ferredoxin

mru0830 ferredoxin

mru1472 ferredoxin

mru2138 ferredoxin

mru0363 flavodoxin

mru1003 flavodoxin

mru1720 flavodoxin

mru0009 flavodoxin domain containing protein

mru0006 flavodoxin domain-containing protein

mru0155 iron-sulfur cluster binding protein

mru0219 NADPH-dependent FMN reductase

mru0364 NADPH-dependent FMN reductase

mru0580 NADPH-dependent FMN reductase

mru1260 NADPH-dependent FMN reductase

mru1369 NADPH-dependent FMN reductase

mru1609 NADPH-dependent FMN reductase

mru1732 NADPH-dependent FMN reductase

mru2135 NADPH-dependent FMN reductase

mru1391 polyferredoxin

mru0701 A1A0 archaeal ATP synthase subunit A AhaA

mru0702 A1A0 archaeal ATP synthase subunit B AhaB

mru0699 A1A0 archaeal ATP synthase subunit C AhaC

mru0703 A1A0 archaeal ATP synthase subunit D AhaD

mru0698 A1A0 archaeal ATP synthase subunit E AhaE

mru0700 A1A0 archaeal ATP synthase subunit F AhaF

mru0695 A1A0 archaeal ATP synthase subunit H AhaH

mru0696 A1A0 archaeal ATP synthase subunit I AhaI

mru0697 A1A0 archaeal ATP synthase subunit K AhaK

**Alcohol metabolism**

mru0065 NADP-dependent alcohol dehydrogenase Adh1

mru1847 NADP-dependent alcohol dehydrogenase Adh2

mru1445 NADP-dependent alcohol dehydrogenase Adh3

mru0991 NADPH-dependent F420 reductase NpdG1

mru1444 NADPH-dependent F420 reductase NpdG2

**Formate metabolism**

mru0681 formate dehydrogenase accessory protein FdhD1

mru1939 formate dehydrogenase accessory protein FdhD2

mru2074 formate dehydrogenase alpha chain FdhA2

mru0333 formate dehydrogenase alpha subunit FdhA1

mru2075 formate dehydrogenase beta chain FdhB2

mru0334 formate dehydrogenase beta subunit FdhB1

mru0332 formate/nitrite transporter FdhC

**H_2_ metabolism**

mru2064 coenzyme F420 hydrogenase alpha subunit FrhA

mru2061 coenzyme F420 hydrogenase beta subunit FrhB1

mru2081 coenzyme F420 hydrogenase beta subunit FrhB2

mru2063 coenzyme F420 hydrogenase delta subunit FrhD

mru2062 coenzyme F420 hydrogenase gamma subunit FrhG

mru1412 energy-converting hydrogenase A subunit A EhaA

mru1411 energy-converting hydrogenase A subunit B EhaB

mru1410 energy-converting hydrogenase A subunit C EhaC

mru1409 energy-converting hydrogenase A subunit D EhaD

mru1408 energy-converting hydrogenase A subunit E EhaE

mru1407 energy-converting hydrogenase A subunit F EhaF

mru1406 energy-converting hydrogenase A subunit G EhaG

mru1405 energy-converting hydrogenase A subunit H EhaH

mru1404 energy-converting hydrogenase A subunit I EhaI

mru1403 energy-converting hydrogenase A subunit J EhaJ

mru1402 energy-converting hydrogenase A subunit K EhaK

mru1401 energy-converting hydrogenase A subunit L EhaL

mru1400 energy-converting hydrogenase A subunit M EhaM

mru1399 energy-converting hydrogenase A subunit N EhaN

mru1398 energy-converting hydrogenase A subunit O EhaO

mru1397 energy-converting hydrogenase A subunit P EhaP

mru1396 energy-converting hydrogenase A subunit Q EhaQ

mru1394 energy-converting hydrogenase A subunit R EhaR

mru2014 energy-converting hydrogenase B subunit A EhbA

mru2013 energy-converting hydrogenase B subunit B EhbB

mru2012 energy-converting hydrogenase B subunit C EhbC

mru2011 energy-converting hydrogenase B subunit D EhbD

mru2010 energy-converting hydrogenase B subunit E EhbE

mru2009 energy-converting hydrogenase B subunit F EhbF

mru2008 energy-converting hydrogenase B subunit G EhbG

mru2007 energy-converting hydrogenase B subunit H EhbH

mru2006 energy-converting hydrogenase B subunit I EhbI

mru2005 energy-converting hydrogenase B subunit J EhbJ

mru2004 energy-converting hydrogenase B subunit K EhbK

mru2003 energy-converting hydrogenase B subunit L EhbL

mru2002 energy-converting hydrogenase B subunit M EhbM

mru2001 energy-converting hydrogenase B subunit N EhbN

mru2000 energy-converting hydrogenase B subunit O EhbO

mru1999 energy-converting hydrogenase B subunit P EhbP

mru1998 energy-converting hydrogenase B subunit Q EhbQ

mru1632 hydrogenase accessory protein HypB

mru0473 hydrogenase assembly chaperone HypC

mru1875 hydrogenase expression/formation protein HypD

mru0190 hydrogenase expression/formation protein HypE1

mru1551 hydrogenase maturation factor HypE2

mru2034 hydrogenase maturation factor HypF

mru1039 hydrogenase maturation protease HycI

mru1633 hydrogenase nickel insertion protein HypA

mru1906 methyl viologen-reducing hydrogenase alpha subunit MvhA

mru1905 methyl viologen-reducing hydrogenase beta subunit MvhB

mru1908 methyl viologen-reducing hydrogenase delta subunit MvhD1

mru2076 methyl viologen-reducing hydrogenase delta subunit MvhD2

mru1907 methyl viologen-reducing hydrogenase gamma subunit MvhG

**Methanogenesis pathway**

mru0569 5,10-methylenetetrahydromethanopterin reductase Mer

mru0117 CoB--CoM heterodisulfide reductase subunit A HdrA

mru0817 CoB--CoM heterodisulfide reductase subunit B HdrB

mru1212 CoB--CoM heterodisulfide reductase subunit B HdrB2

mru0816 CoB--CoM heterodisulfide reductase subunit C HdrC

mru0526 coenzyme F420-dependent N(5),N(10)-methenyltetrahydromethanopterin reductase Hmd

mru2142 F420-dependent methylenetetrahydromethanopterin dehydrogenase Mtd

mru1393 formylmethanofuran-tetrahydromethanopterin formyltransferase Ftr1

mru2022 formylmethanofuran-tetrahydromethanopterin formyltransferase Ftr2

mru1619 methenyltetrahydromethanopterin cyclohydrolase Mch

mru1924 methyl-coenzyme M reductase alpha subunit McrA

mru1928 methyl-coenzyme M reductase beta subunit McrB

mru1926 methyl-coenzyme M reductase C subunit McrC

mru1262 methyl-coenzyme M reductase component A2 AtwA1

mru1850 methyl-coenzyme M reductase component A2 AtwA2

mru1927 methyl-coenzyme M reductase D subunit McrD

mru1925 methyl-coenzyme M reductase gamma subunit McrG

mru1919 tetrahydromethanopterin S-methyltransferase subunit A MtrA1

mru0441 tetrahydromethanopterin S-methyltransferase subunit A MtrA2

mru1920 tetrahydromethanopterin S-methyltransferase subunit B MtrB

mru1921 tetrahydromethanopterin S-methyltransferase subunit C MtrC

mru1922 tetrahydromethanopterin S-methyltransferase subunit D MtrD

mru1923 tetrahydromethanopterin S-methyltransferase subunit E MtrE

mru1918 tetrahydromethanopterin S-methyltransferase subunit F MtrF

mru1917 tetrahydromethanopterin S-methyltransferase subunit G MtrG

mru1916 tetrahydromethanopterin S-methyltransferase subunit H MtrH

mru0344 tungsten formylmethanofuran dehydrogenase subunit A FwdA

mru0343 tungsten formylmethanofuran dehydrogenase subunit B FwdB

mru0345 tungsten formylmethanofuran dehydrogenase subunit C FwdC

mru0342 tungsten formylmethanofuran dehydrogenase subunit D FwdD

mru0254 tungsten formylmethanofuran dehydrogenase subunit E FwdE

mru0340 tungsten formylmethanofuran dehydrogenase subunit F FwdF

mru0341 tungsten formylmethanofuran dehydrogenase subunit G FwdG

mru0339 tungsten formylmethanofuran dehydrogenase subunit H FwdH

**Other**

mru1695 H_4_MPT-linked C1 transfer pathway protein

*************************************

**LIPID METABOLISM**

**Biosynthesis bacterial**

mru1031 3-oxoacyl-(acyl-carrier-protein) reductase FabG1

mru1630 3-oxoacyl-(acyl-carrier-protein) reductase FabG2

mru1289 diacylglycerol kinase DagK

mru2188 glycerol-3-phosphate dehydrogenase (NAD)

**Biosynthesis general**

mru1341 geranylgeranyl reductase family protein

mru1441 geranylgeranyl reductase family protein

mru1471 geranylgeranyl reductase family protein

**Lipid backbone**

mru0955 NAD(P)-dependent glycerol-1-phosphate dehydrogenase EgsA

**Phospholipid biosynthesis**

mru1885 digeranylgeranylglyceryl phosphate synthase

mru1102 geranylgeranylglyceryl phosphate synthase

mru0503 phosphatidylglycerophosphate synthase PgsA

mru1833 phosphatidylserine decarboxylase Psd

mru1834 phosphatidylserine synthase PssA

**Mevalonate pathway**

mru1639 acetyl-CoA acetyltransferase

mru1092 hydroxymethylglutaryl-CoA reductase (NADPH) HmgA

mru1640 hydroxymethylglutaryl-CoA synthase

mru0922 isopentenyl diphosphate delta-isomerase Fni

mru0921 isopentenyl diphosphate kinase

mru0920 mevalonate kinase Mvk

mru0919 phosphomevalonate decarboxylase

**Elongation of isoprenoid side chains**

mru0924 bifunctional short chain isoprenyl diphosphate synthase IdsA

*************************************

**MOBILE ELEMENTS**

**CRISPR-associated genes**

mru0796 CRISPR-associated helicase Cas3

mru0798 CRISPR-associated protein Cas1-1

mru1174 CRISPR-associated protein Cas1-2

mru1647 CRISPR-associated protein Cas1-3

mru1648 CRISPR-associated protein Cas1-4

mru0799 CRISPR-associated protein Cas2-1

mru1176 CRISPR-associated protein Cas2-2

mru1649 CRISPR-associated protein Cas2-3

mru0797 CRISPR-associated protein Cas4-1

mru1549 CRISPR-associated protein Cas4-2

mru0795 CRISPR-associated protein Cas5 Hmari subtype

mru0792 CRISPR-associated protein Cas6

mru1183 CRISPR-associated protein Csm1 family

mru1182 CRISPR-associated protein Csm2 family

mru0794 CRISPR-associated protein CT1132 family

mru1178 CRISPR-associated protein TIGR02710 family

mru1188 CRISPR-associated protein TIGR02710 family

mru1181 CRISPR-associated RAMP protein Csm3 family

mru1180 CRISPR-associated RAMP protein Csm4 family

mru1179 CRISPR-associated RAMP protein Csm5 family

**Prophage**

mru0057 phage-related protein

mru0058 phage-related protein

mru0256 phage integrase

mru0257 hypothetical protein

mru0258 hypothetical protein

mru0259 cdc6 family replication initiation protein Cdc6-3

mru0260 hypothetical protein

mru0262 hypothetical protein

mru0263 hypothetical protein

mru0264 hypothetical protein

mru0265 hypothetical protein

mru0266 hypothetical protein

mru0267 hypothetical protein

mru0268 hypothetical protein

mru0269 ATPase involved in DNA replication control MCM family

mru0270 phage-related protein

mru0271 hypothetical protein

mru0272 hypothetical protein

mru0273 hypothetical protein

mru0274 hypothetical protein

mru0275 hypothetical protein

mru0276 hypothetical protein

mru0277 hypothetical protein

mru0278 hypothetical protein

mru0279 hypothetical protein

mru0280 ParB-like nuclease domain-containing protein

mru0281 hypothetical protein

mru0282 phage-related protein

mru0283 hypothetical protein

mru0284 phage-related protein

mru0285 terminase large subunit

mru0286 hypothetical protein

mru0287 phage portal protein

mru0288 phage-related protein

mru0289 hypothetical protein

mru0290 hypothetical protein

mru0291 hypothetical protein

mru0292 hypothetical protein

mru0293 hypothetical protein

mru0294 hypothetical protein

mru0295 hypothetical protein

mru0296 hypothetical protein

mru0297 hypothetical protein

mru0298 hypothetical protein

mru0299 hypothetical protein

mru0300 hypothetical protein

mru0301 hypothetical protein

mru0302 hypothetical protein

mru0303 hypothetical protein

mru0305 hypothetical protein

mru0306 hypothetical protein

mru0307 phage-related protein

mru0308 phage-related protein

mru0309 hypothetical protein

mru0310 phage-related protein

mru0311 phage-related protein

mru0312 hypothetical protein

mru0313 phage-related protein

mru0314 hypothetical protein

mru0315 phage tail tape measure protein

mru0316 phage-related protein

mru0317 phage-related protein

mru0318 hypothetical protein

mru0319 hypothetical protein

mru0320 endoisopeptidase PeiR

mru0321 dnd system-associated protein 3

mru0322 dnd system-associated protein 1

mru0323 dnd system-associated protein 2

mru0324 type II restriction enzyme, methylase subunit

mru0325 hypothetical protein

**Transposase**

mru0119 transposase

mru0355 transposase

mru0434 transposase

mru0578 transposase

mru0623 transposase remnant

mru0624 transposase remnant

mru0625 transposase remnant

mru0777 transposase

mru0948 transposase

mru0992 transposase

mru1162 transposase

mru1583 transposase

mru1608 transposase

mru1662 transposase

mru1766 transposase

mru2148 transposase

*************************************

**NITROGEN METABOLISM**

**Fixation**

mru1466 4Fe-4S iron sulfur cluster binding protein NifH/frxC family

mru1567 NifU-like FeS cluster assembly scaffold protein

**General**

mru0094 nitroreductase family protein

mru0471 nitroreductase family protein

mru0692 nitroreductase family protein

mru0994 nitroreductase family protein

mru0995 nitroreductase family protein

mru1941 nitroreductase family protein

**Other**

mru0749 ADP-ribosylglycohydrolase family protein

mru1455 ADP-ribosylglycohydrolase family protein

mru1580 ADP-ribosylglycohydrolase family protein

mru2121 hydroxylamine reductase Hcp

**Regulation**

mru1324 nitrogen regulatory protein P-II GlnK

**Transport**

mru1325 ammonium transporter Amt

*************************************

**NUCLEIC ACID METABOLISM**

**DNA-binding proteins**

mru0011 archaeal histone

mru0774 archaeal histone

mru1491 archaeal histone

mru1686 archaeal histone

mru1731 archaeal histone

mru1760 archaeal histone

mru0397 DNA-binding protein

mru1684 histone acetyltransferase ELP3 family

mru0606 NAD-dependent protein deacetylase

**Helicase**

mru0133 ATP-dependent DNA helicase

mru1111 ATP-dependent DNA helicase UvrD/REP family

mru1138 ATP-dependent DNA helicase UvrD/REP family

mru1184 ATP-dependent DNA helicase UvrD/REP family

mru0600 DEAD/DEAH box helicase domain-containing protein

mru0778 DEAD/DEAH box helicase domain-containing protein

mru1110 DEAD/DEAH box helicase domain-containing protein

mru1319 DEAD/DEAH box helicase domain-containing protein

mru1681 DEAD/DEAH box helicase domain-containing protein

mru1121 DNA helicase

mru1157 helicase RecD/TraA family

mru0620 helicase SNF2 family

mru0981 Rad3-related DNA helicase

**Recombination and repair**

mru1575 6-O-methylguanine DNA methyltransferase Ogt

mru0136 8-oxoguanine DNA glycosylase Ogg

mru2027 archaeal Holliday junction resolvase Hjc

mru1566 archaea-specific RecJ-like exonuclease

mru1105 DNA double-strand break repair protein Mre11

mru1106 DNA double-strand break repair protein Rad50

mru1429 DNA mismatch endonuclease Vsr

mru0583 DNA mismatch repair ATPase MutS family

mru0115 DNA repair and recombination protein RadA

mru0505 DNA repair and recombination protein RadB

mru2068 DNA-3-methyladenine glycosylase I Tag

mru1576 endonuclease III Nth

mru1855 endonuclease IV

mru1127 excinuclease ABC A subunit UvrA1

mru1256 excinuclease ABC A subunit UvrA2

mru1087 excinuclease ABC B subunit UvrB

mru1223 excinuclease ABC C subunit UvrC

mru1115 exodeoxyribonuclease III Xth1

mru1557 exodeoxyribonuclease III Xth2

mru0812 exodeoxyribonuclease VII large subunit XseA

mru0813 exodeoxyribonuclease VII small subunit XseB

mru0770 exonuclease

mru2089 Hef nuclease

mru2099 RdgB/HAM1 family non-canonical purine NTP pyrophosphatase

mru1547 ssDNA exonuclease RecJ2

mru2097 ssDNA exonuclease RecJ1

mru1383 staphylococcal nuclease domain-containing protein

mru0218 uracil-DNA glycosylase Ung

**Restriction and modification**

mru0029 5-methylcytosine restriction system component protein

mru1167 DNA modification methylase

mru0026 DNA-cytosine methyltransferase

mru0027 DNA-cytosine methyltransferase

mru1135 restriction endonuclease

mru1165 restriction enzyme methylase subunit

mru0927 type I restriction-modification enzyme S subunit HsdS

mru0928 type I restriction-modification system M subunit HsdM

mru1166 type II restriction endonuclease

mru2106 type II restriction endonuclease

*************************************

**PROTEIN FATE**

**Protein degradation**

mru1637 ATP-dependent protease S16 family

mru2100 glycoprotease M22 family

mru2058 methionine aminopeptidase Map

mru1028 peptidase C39 family

mru2168 peptidase C39 family

mru1128 peptidase M48 family

mru1504 peptidase M48 family

mru1755 peptidase M48 family

mru0130 peptidase M50 family

mru0238 peptidase M50 family

mru1698 peptidase M50 family

mru1677 peptidase S49 family

mru0584 peptidase U32 family

mru0585 peptidase U32 family

mru1467 peptidase U32 family

mru1727 peptidase U62 family

mru1873 peptidase U62 family

mru2021 transglutaminase domain-containing protein

mru0669 Xaa-Pro aminopeptidase

**Protein folding**

mru1305 DnaK-related protein

mru1812 DnaK-related protein

mru1730 heat shock protein Hsp20/alpha crystallin family

mru2040 molecular chaperone DnaJ

mru2039 molecular chaperone DnaK

mru2038 molecular chaperone GrpE

mru1511 nascent polypeptide-associated complex protein

mru0145 peptidyl-prolyl cis-trans isomerase

mru1901 peptidyl-prolyl cis-trans isomerase

mru0403 prefoldin alpha subunit PfdA

mru1347 prefoldin beta subunit PfdB

mru1357 proteasome alpha subunit

mru1977 proteasome beta subunit

mru1448 proteasome-activating nucleotidase

mru1501 thermosome subunit

mru1645 thermosome subunit

**Protein secretion**

mru0391 oligosaccharyl transferase

mru0482 preprotein translocase subunit SecE

mru0239 preprotein translocase subunit SecG

mru0875 preprotein translocase subunit SecY

mru1722 protein export membrane protein SecD

mru1721 protein export membrane protein SecF

mru1581 signal peptidase I

mru2118 signal peptidase I

mru0404 signal recognition particle receptor FtsY

mru1546 signal recognition particle SRP19 protein

mru0164 signal recognition particle SRP54 protein

mru1832 sortase family protein

mru0522 type II secretion system protein E GspE

mru0524 type II secretion system protein F GspF1

mru0670 type II secretion system protein F GspF2

mru1234 type IV leader peptidase family protein

*************************************

**PROTEIN SYNTHESIS**

**Other**

mru0507 ATPase RIL

mru0508 peptidyl-tRNA hydrolase

mru0163 pseudouridylate synthase

mru2127 ribonuclease

mru2128 ribonuclease inhibitor

mru1349 ribosomal biogenesis protein

mru1038 RNA methylase

mru1440 RNA methylase

mru0159 RNA-binding protein

mru0395 RNA-binding protein

mru0475 RNA-binding protein

mru0519 RNA-binding protein

mru0729 RNA-binding protein

mru1284 RNA-binding protein

mru1866 RNA-binding protein

mru0923 RNA-metabolising metallo-beta-lactamase

mru1978 RNA-metabolising metallo-beta-lactamase

mru1610 rRNA methylase

mru0504 Sua5/YciO/YrdC/YwlC family translation factor

mru0180 translation-associated GTPase

mru0398 tRNA methyltransferase subunit

**Ribosomal proteins**

mru0486 acidic ribosomal protein P0 RplPO

mru1898 ribosomal protein L10e Rpl10e

mru0484 ribosomal protein L11P Rpl11p

mru0487 ribosomal protein L12P Rpl12p

mru0910 ribosomal protein L13P Rpl13p

mru0880 ribosomal protein L14e Rpl14e

mru0862 ribosomal protein L14P Rpl14p

mru1362 ribosomal protein L15e Rpl15e

mru0874 ribosomal protein L15P Rpl15p

mru0909 ribosomal protein L18e Rpl18e

mru0871 ribosomal protein L18P Rpl18p

mru0870 ribosomal protein L19e Rpl19e

mru0485 ribosomal protein L1P Rpl1p

mru0162 ribosomal protein L21e Rpl21e

mru0856 ribosomal protein L22P Rpl22p

mru0853 ribosomal protein L23P Rpl23p

mru0483 ribosomal protein L24 family

mru1488 ribosomal protein L24e Rpl24e

mru0863 ribosomal protein L24P Rpl24p

mru0858 ribosomal protein L29P Rpl29p

mru0854 ribosomal protein L2P Rpl2p

mru1810 ribosomal protein L30e Rpl30e

mru0873 ribosomal protein L30P Rpl30p

mru0400 ribosomal protein L31e Rpl31e

mru0869 ribosomal protein L32e Rpl32e

mru0878 ribosomal protein L34e Rpl34e

mru1351 ribosomal protein L37Ae Rpl37ae

mru1281 ribosomal protein L37e Rpl37e

mru0399 ribosomal protein L39e Rpl39e

mru0851 ribosomal protein L3P Rpl3p

mru1100 ribosomal protein L40e Rpl40e

mru0175 ribosomal protein L44e Rpl44e

mru0852 ribosomal protein L4p Rpl4p

mru0865 ribosomal protein L5P Rpl5p

mru0868 ribosomal protein L6P Rpl6p

mru1490 ribosomal protein L7Ae Rpl7ae

mru0402 ribosomal protein LX RplX

mru1804 ribosomal protein S10P Rps10p

mru0907 ribosomal protein S11P Rps11p

mru1808 ribosomal protein S12P Rps12p

mru0905 ribosomal protein S13P Rps13p

mru0866 ribosomal protein S14P Rps14p

mru2098 ribosomal protein S15P Rps15p

mru1673 ribosomal protein S17e Rps17e

mru0861 ribosomal protein S17P Rps17p

mru0396 ribosomal protein S19e Rps19e

mru0855 ribosomal protein S19P Rps19p

mru1478 ribosomal protein S24e Rps24e

mru1477 ribosomal protein S27ae Rps27ae

mru0176 ribosomal protein S27e Rps27e

mru1489 ribosomal protein S28e Rps28e

mru0916 ribosomal protein S2P Rps2p

mru0461 ribosomal protein S3Ae Rps3ae

mru0857 ribosomal protein S3P Rps3p

mru0864 ribosomal protein S4e Rps4e

mru0906 ribosomal protein S4P Rps4p

mru0872 ribosomal protein S5P Rps5p

mru1485 ribosomal protein S6e Rps6e

mru1807 ribosomal protein S7P Rps7p

mru1552 ribosomal protein S8e Rps8e

mru0867 ribosomal protein S8P Rps8p

mru0911 ribosomal protein S9P Rps9p

**RNA processing**

mru0996 2'-5' RNA ligase LigT

mru0439 7-cyano-7-deazaguanosine biosynthesis protein QueE

mru0525 archaeal fibrillarin-like protein

mru0814 archaeosine tRNA-ribosyltransferase TgtA1

mru1512 archaeosine tRNA-ribosyltransferase TgtA2

mru0158 dimethyladenosine transferase KsgA

mru1353 exosome complex exonuclease Rrp41

mru0168 exosome complex RNA-binding protein Csl4

mru1354 exosome complex RNA-binding protein Rrp4

mru1352 exosome complex RNA-binding protein Rrp42

mru1355 exosome subunit

mru1361 exosome subunit

mru1988 fibrillarin

mru0898 H/ACA RNA-protein complex component Cbf5p

mru0713 H/ACA RNA-protein complex component Gar1

mru0178 H/ACA RNA-protein complex component Nop10p

mru1687 MiaB-like tRNA modifying enzyme

mru1960 N2,N2-dimethylguanosine tRNA methyltransferase Trm1

mru0589 NMD3 family protein

mru1987 pre-mRNA splicing ribonucleoprotein PRP31

mru0437 queuosine biosynthesis protein QueC

mru0438 queuosine biosynthesis protein QueD

mru1359 ribonuclease P subunit P14

mru0860 ribonuclease P subunit P29

mru1360 ribonuclease P subunit P30

mru0394 ribonuclease P subunit RPR2

mru0617 ribonuclease Z Rnz

mru0849 ribosomal protein L11 methyltransferase PrmA

mru0593 ribosomal RNA large subunit methyltransferase J RrmJ

mru1800 ribosomal-protein-alanine acetyltransferase RimI

mru1025 RNA ligase DRB0094 family

mru0654 RNA methyltransferase TrmH family

mru0157 SAM-dependent methyltransferase HemK-related

mru1495 tRNA intron endonuclease EndA

mru0987 tRNA nucleotidyltransferase Cca

mru1703 tRNA pseudouridine synthase A TruA

mru0197 tRNA pseudouridine synthase D TruD

mru2088 tRNA(1-methyladenosine) methyltransferase

mru0676 tRNA(His) guanylyltransferase ThgL

mru0096 tRNA-dihydrouridine synthase DusA1

mru1846 tRNA-dihydrouridine synthase DusA2

mru1823 tRNA-modifying enzyme

**Translation factors**

mru0469 cell division protein pelota PelA

mru1053 deoxyhypusine synthase Dys

mru0166 diphthamide biosynthesis protein

mru1764 diphthine synthase DphB

mru0728 peptide chain release factor aRF1

mru1805 translation elongation factor aEF-1 alpha

mru0520 translation elongation factor aEF-1 beta

mru1300 translation elongation factor aEF-2

mru1806 translation elongation factor aEF-2

mru1869 translation initiation factor aIF-1A

mru0177 translation initiation factor aIF-2 alpha subunit

mru0590 translation initiation factor aIF-2 beta subunit

mru1484 translation initiation factor aIF-2 gamma subunit

mru1768 translation initiation factor aIF-2B alpha subunit

mru1742 translation initiation factor aIF-5A

mru0401 translation initiation factor aIF-6

mru0859 translation initiation factor aSUI1

mru1486 translation initiation factor IF-2

**tRNA aminoacylation**

mru0492 alanyl-tRNA synthetase AlaS

mru2117 arginyl-tRNA synthetase ArgS

mru1014 aspartyl-tRNA synthetase AspS

mru2169 Asp-tRNA(Asn)/Glu-tRNA(Gln) amidotransferase subunit A GatA

mru2029 Asp-tRNA(Asn)/Glu-tRNA(Gln) amidotransferase subunit B GatB

mru1142 Asp-tRNA(Asn)/Glu-tRNA(Gln) amidotransferase subunit C GatC

mru1571 cysteinyl-tRNA synthetase CysS

mru0137 D-tyrosyl-tRNA(Tyr) deacylase

mru0938 glutamyl-tRNA synthetase GltX

mru1427 glutamyl-tRNA(Gln) amidotransferase subunit D GatD

mru1426 glutamyl-tRNA(Gln) amidotransferase subunit E GatE

mru0651 glycyl-tRNA synthetase GlyS

mru1248 histidyl-tRNA synthetase HisS

mru0126 isoleucyl-tRNA synthetase IleS

mru2077 leucyl-tRNA synthetase LeuS

mru0242 lysyl-tRNA synthetase LysS

mru0959 methionyl-tRNA synthetase MetG

mru1558 phenylalanyl-tRNA synthetase alpha subunit PheS

mru1586 phenylalanyl-tRNA synthetase subunit beta PheT

mru0954 prolyl-tRNA synthetase ProS

mru1947 seryl-tRNA synthetase SerS

mru2129 threonyl-tRNA synthetase ThrS

mru0673 tRNA binding domain-containing protein

mru1494 tryptophanyl-tRNA synthetase TrpS

mru0588 tyrosyl-tRNA synthetase TyrS

mru1584 valyl-tRNA synthetase ValS

*************************************

**PURINES AND PYRIMIDINES**

**Purine biosynthesis**

mru0595 5-formaminoimidazole-4-carboxamide-1-(beta)-D- ribofuranosyl 5'-monophosphate-formate ligase PurP

mru0165 adenine phosphoribosyltransferase Apt

mru0229 adenylosuccinate lyase PurB

mru1468 amidophosphoribosyltransferase PurF

mru1839 IMP cyclohydrolase PurO

mru2113 phosphoribosylamine-glycine ligase PurD

mru2033 phosphoribosylaminoimidazole carboxylase catalytic subunit PurE1

mru2186 phosphoribosylaminoimidazole carboxylase purE2

mru1538 phosphoribosylaminoimidazole-succinocarboxamide synthase PurC

mru0127 phosphoribosylformylglycinamidine (FGAM) synthase II PurL

mru1540 phosphoribosylformylglycinamidine (FGAM) synthase PurQ

mru1539 phosphoribosylformylglycinamidine (FGAM) synthase PurS

mru1979 phosphoribosylformylglycinamidine cyclo-ligase PurM

**Purine interconversion**

mru1736 adenine deaminase Ade

mru0386 adenylate cyclase CyaA

mru0876 adenylate kinase Adk

mru0946 adenylosuccinate synthetase PurA

**Pyrimidine biosynthesis**

mru0005 aspartate carbamoyltransferase PyrB

mru1725 aspartate carbamoyltransferase regulatory subunit PyrI

mru1791 carbamoyl-phosphate synthase large subunit CarB

mru1792 carbamoyl-phosphate synthase small subunit CarA

mru1904 dihydroorotase PyrC

mru1985 dihydroorotate dehydrogenase electron transfer subunit PyrK

mru1986 dihydroorotate dehydrogenase PyrD

mru1748 orotate phosphoribosyltransferase PyrE1

mru1783 orotate phosphoribosyltransferase PyrE2

mru1055 orotidine 5'-phosphate decarboxylase PyrF

**Pyrimidine interconversion**

mru1286 CMP/dCMP deaminase

mru2199 CMP/dCMP deaminase

mru1237 CTP synthase PyrG

mru0879 cytidylate kinase Cmk

mru0652 deoxycytidine triphosphate deaminase Dcd

mru1048 dUTP diphosphatase Dut

mru1425 thioredoxin-disulfide reductase TrxB

mru0586 thymidylate kinase Tmk1

mru1226 thymidylate kinase Tmk2

mru2105 thymidylate synthase ThyA

mru0722 uridylate kinase PyrH

**Interconversion**

mru1422 GMP synthase subunit A GuaA

mru1420 GMP synthase subunit B GuaAb

mru1208 inosine-5'-monophosphate dehydrogenase GuaB

mru2104 5'-nucleotidase SurE1

mru0420 5'-nucleotidase SurE2

mru0241 anaerobic ribonucleoside-triphosphate reductase NrdD

mru1487 nucleoside diphosphate kinase Ndk

**Salvage**

mru1372 uracil phosphoribosyltransferase Upp

**Transport**

mru1373 xanthine/uracil permease

*************************************

**REGULATION**

**Protein interaction**

mru0039 biotin-binding and phosphotyrosine protein phosphatase domain-containing protein

mru0582 phosphate uptake regulator PhoU

mru1295 phosphotyrosine protein phosphatase

mru0044 serine phosphatase

mru1306 serine/threonine protein kinase

mru1868 serine/threonine protein kinase RIO1 family

mru1168 serine/threonine protein kinase with TPR repeats

mru1288 serine/threonine protein phosphatase

mru0452 TPR repeat-containing protein

mru0748 TPR repeat-containing protein

mru0800 TPR repeat-containing protein

mru1018 TPR repeat-containing protein

mru1086 TPR repeat-containing protein

mru1186 TPR repeat-containing protein

mru1216 TPR repeat-containing protein

mru1316 TPR repeat-containing protein

mru1813 TPR repeat-containing protein

mru1825 TPR repeat-containing protein

mru2116 TPR repeat-containing protein

mru2166 TPR repeat-containing protein

mru2167 TPR repeat-containing protein

**Transcriptional regulator**

mru1338 iron dependent repressor

mru1496 iron dependent repressor

mru1037 nickel responsive transcriptional regulator NikR

mru0073 transcriptional regulator

mru0232 transcriptional regulator

mru0430 transcriptional regulator

mru0649 transcriptional regulator

mru0650 transcriptional regulator

mru0684 transcriptional regulator

mru1052 transcriptional regulator

mru1134 transcriptional regulator

mru1160 transcriptional regulator

mru1327 transcriptional regulator

mru1435 transcriptional regulator

mru1447 transcriptional regulator

mru1449 transcriptional regulator

mru1871 transcriptional regulator

mru1944 transcriptional regulator

mru2122 transcriptional regulator

mru2165 transcriptional regulator AbrB family

mru0132 transcriptional regulator ArsR family

mru0365 transcriptional regulator ArsR family

mru1334 transcriptional regulator ArsR family

mru1446 transcriptional regulator ArsR family

mru1762 transcriptional regulator ArsR family

mru2037 transcriptional regulator ArsR family

mru1961 transcriptional regulator AsnC family

mru0359 transcriptional regulator HxlR family

mru0370 transcriptional regulator HxlR family

mru0249 transcriptional regulator LysR family

mru0662 transcriptional regulator LytR family

mru0138 transcriptional regulator MarR family

mru0217 transcriptional regulator MarR family

mru0442 transcriptional regulator MarR family

mru0815 transcriptional regulator MarR family

mru0988 transcriptional regulator MarR family

mru1548 transcriptional regulator MarR family

mru1556 transcriptional regulator MarR family

mru1629 transcriptional regulator MarR family

mru0142 transcriptional regulator TetR family

mru0577 transcriptional regulator TetR family

mru0645 transcriptional regulator TetR family

mru1739 transcriptional regulator TetR family

mru0220 transcriptional regulator TetR family with acetyltransferase GNAT family domain

**Other**

mru0187 carbon starvation protein CstA

mru0576 sugar fermentation stimulation protein SfsA1

mru2018 sugar fermentation stimulation protein SfsA2

*************************************

**SECONDARY METABOLITES**

**NRPS**

mru0068 non-ribosomal peptide synthetase

mru0351 non-ribosomal peptide synthetase

**Other**

mru0514 4'-phosphopantetheinyl transferase family protein

mru0512 acyltransferase

mru0067 anti-sigma factor antagonist

mru0513 anti-sigma factor antagonist

mru0516 anti-sigma regulatory factor serine/threonine protein kinase

mru0069 MatE efflux family protein

mru0352 MatE efflux family protein

mru0066 serine phosphatase

mru0071 serine phosphatase

mru0515 serine phosphatase

*************************************

**TRANSCRIPTION**

**RNA polymerase**

mru1482 DNA-dependent RNA polymerase subunit E' RpoE1

mru1481 DNA-dependent RNA polymerase subunit E'' RpoE2

mru1815 DNA-directed RNA polymerase subunit A' RpoA1

mru1814 DNA-directed RNA polymerase subunit A'' RpoA2

mru1816 DNA-directed RNA polymerase subunit B' RpoB1

mru1817 DNA-directed RNA polymerase subunit B'' RpoB2

mru0908 DNA-directed RNA polymerase subunit D RpoD

mru0161 DNA-directed RNA polymerase subunit F RpoF

mru1818 DNA-directed RNA polymerase subunit H RpoH

mru0913 DNA-directed RNA polymerase subunit K RpoK

mru0169 DNA-directed RNA polymerase subunit L RpoL

mru0912 DNA-directed RNA polymerase subunit N RpoN

mru1350 DNA-directed RNA polymerase subunit P RpoP

**Translation factors**

mru0387 TATA-box binding protein Tbp

mru1809 transcription elongation factor NusA-like protein

mru0546 transcription factor S Tfs1

mru0171 transcription factor S Tfs2

mru0714 transcription initiation factor TFIIB Tfb1

mru1600 transcription initiation factor TFIIB Tfb2

mru0478 transcription initiation factor TFIIE alpha subunit Tfe

**Other**

mru1282 LSM domain-containing protein

mru2102 LSM domain-containing protein

mru1278 ribonuclease III Rnc

mru0837 RNA-binding protein

mru2179 RNA-binding S1 domain-containing protein

mru1119 glutamyl aminopeptidase PepA

*************************************

**TRANSPORTERS**

**Amino acids**

mru1775 amino acid ABC transporter ATP-binding protein

mru1776 amino acid ABC transporter permease protein

mru1777 amino acid ABC transporter substrate-binding protein

mru1945 amino acid ABC transporter substrate-binding protein

mru1759 amino acid carrier protein AGCS family

**Anions**

mru0467 voltage-gated chloride channel protein

**Cations**

mru1802 cation-transporting P-type ATPase

mru0205 copper ion binding protein

mru2208 divalent cation transporter mgtE family

mru0536 ferrous iron transport protein A FeoA

mru1340 ferrous iron transport protein B FeoB1

mru0537 ferrous iron transport protein B FeoB2

mru0206 heavy metal translocating P-type ATPase

mru1612 heavy metal translocating P-type ATPase

mru1861 heavy metal translocating P-type ATPase

mru1333 heavy metal-translocating P-type ATPase

mru0789 ion transport protein

mru0808 ion transport protein

mru1083 ion transport protein

mru1953 K+-dependent Na+/Ca+ exchanger

mru1615 nickel ABC transporter ATP-binding protein NikD1

mru1706 nickel ABC transporter ATP-binding protein NikD2

mru1614 nickel ABC transporter ATP-binding protein NikE1

mru1705 nickel ABC transporter ATP-binding protein NikE2

mru1617 nickel ABC transporter permease protein NikB1

mru1709 nickel ABC transporter permease protein NikB2

mru1616 nickel ABC transporter permease protein NikC1

mru1708 nickel ABC transporter permease protein NikC2

mru1618 nickel ABC transporter substrate-binding protein NikA1

mru1710 nickel ABC transporter substrate-binding protein NikA2

mru2020 potassium channel protein

mru2025 potassium uptake protein TrkA family

mru0207 potassium uptake protein TrkH family

mru2024 potassium uptake protein TrkH family

mru0821 transporter CDF family

mru0827 transporter CDF family

mru0405 transporter Na+/H+ antiporter family

mru2209 TrkA domain-containing protein

**Other**

mru0002 ABC transporter ATP-binding protein

mru0253 ABC transporter ATP-binding protein

mru1438 ABC transporter ATP-binding protein

mru1701 ABC transporter ATP-binding protein

mru1714 ABC transporter ATP-binding protein

mru2207 ABC transporter ATP-binding protein

mru0366 ABC transporter ATP-binding/permease protein

mru1627 ABC transporter ATP-binding/permease protein

mru1628 ABC transporter ATP-binding/permease protein

mru0003 ABC transporter permease protein

mru0252 ABC transporter permease protein

mru1437 ABC transporter permease protein

mru1702 ABC transporter permease protein

mru1713 ABC transporter permease protein

mru2206 ABC transporter permease protein

mru0251 ABC transporter substrate-binding protein

mru0216 MatE efflux family protein

mru0609 MatE efflux family protein

mru1439 MatE efflux family protein

mru1658 MatE efflux family protein

mru1735 MatE efflux family protein

mru1751 MatE efflux family protein

mru1765 MatE efflux family protein

mru0616 mechanosensitive ion channel protein

mru0046 MFS transporter

mru0139 MFS transporter

mru0140 MFS transporter

mru0215 MFS transporter

mru0379 MFS transporter

mru0559 MFS transporter

mru1002 MFS transporter

mru1191 MFS transporter

mru1201 MFS transporter

mru1968 MFS transporter

mru0329 MotA/TolQ/ExbB proton channel family protein

mru0705 MotA/TolQ/ExbB proton channel family protein

mru1082 MotA/TolQ/ExbB proton channel family protein

mru2045 MotA/TolQ/ExbB proton channel family protein

mru2051 MotA/TolQ/ExbB proton channel family protein

mru1332 Na+ dependent transporter SBF family

mru0008 Na+-dependent transporter SNF family

mru0406 Na+-dependent transporter SNF family

mru0407 Na+-dependent transporter SNF family

mru0636 Na+-dependent transporter SNF family

mru1202 Na+-dependent transporter SNF family

mru1285 Na+-dependent transporter SNF family

mru2197 Na+-dependent transporter SNF family

mru0116 transporter

mru0141 transporter

mru1840 transporter ExbD/Tol family

mru1370 transporter MIP family

mru1841 transporter MotA/TolQ/ExbB proton channel family

mru2176 transporter permease family protein

mru2177 transporter permease family protein

mru0986 transporter SDF family

mru1789 transporter SDF family

mru0358 transporter small multidrug resistance (SMR) family

mru0369 transporter small multidrug resistance (SMR) family

mru0993 transporter TDT family

*************************************

**UNKNOWN FUNCTION**

**Enzyme**

mru0422 acetyltransferase

mru0455 acetyltransferase

mru1473 acetyltransferase

mru1758 acetyltransferase

mru1881 acetyltransferase

mru2170 acetyltransferase

mru0500 acetyltransferase GNAT family

mru0574 acetyltransferase GNAT family

mru0612 acetyltransferase GNAT family

mru0633 acetyltransferase GNAT family

mru1374 acetyltransferase GNAT family

mru1707 acetyltransferase GNAT family

mru1712 acetyltransferase GNAT family

mru2032 acetyltransferase GNAT family

mru2198 acetyltransferase GNAT family

mru1328 acyl-CoA synthetase

mru0248 acyltransferase

mru1534 acyltransferase

mru0087 amidohydrolase

mru0203 amidohydrolase

mru0610 amidohydrolase

mru0664 amidohydrolase

mru0826 aminotransferase

mru1940 aminotransferase

mru1959 aminotransferase DegT/DnrJ/EryC1/StrS family

mru1032 AMP-binding enzyme

mru1298 archaeal ATPase

mru2140 archaeal ATPase

mru0014 ATPase

mru0040 ATPase

mru0075 ATPase

mru0534 ATPase

mru0560 ATPase

mru1104 ATPase

mru1170 ATPase

mru1854 ATPase

mru1860 ATPase

mru2070 ATPase

mru0415 calcineurin-like phosphoesterase

mru0820 calcineurin-like phosphoesterase

mru2019 carbohydrate kinase

mru0246 carbohydrate kinase PfkB family

mru1329 carbohydrate kinase PfkB family

mru1392 carbohydrate kinase PfkB family

mru0708 CobB/CobQ-like glutamine amidotransferase domain-containing protein

mru1040 D-alanine-D-alanine ligase

mru0712 demethylmenaquinone methyltransferase

mru0825 Fe-S oxidoreductase

mru0052 glycyl-radical enzyme activating protein

mru2192 glycyl-radical enzyme activating protein

mru1723 GMC oxidoreductase family protein

mru0491 hydrolase alpha/beta fold family

mru0511 hydrolase alpha/beta fold family

mru0771 hydrolase alpha/beta fold family

mru1036 hydrolase alpha/beta fold family

mru1508 hydrolase alpha/beta fold family

mru1554 hydrolase HAD superfamily

mru2163 hydrolase HAD superfamily

mru0226 hydrolase TatD family

mru0648 hydrolase TatD family

mru0721 hydrolase TatD family

mru1006 hydrolase TatD family

mru0929 manganese-dependent inorganic pyrophosphatase PpaC

mru0834 metallo-beta-lactamase superfamily protein

mru1562 metallo-beta-lactamase superfamily protein

mru2026 metallo-beta-lactamase superfamily protein

mru1502 methyltransferase

mru2164 NADH:flavin oxidoreductase/NADH oxidase family protein

mru1757 NADH-dependent flavin oxidoreductase

mru0151 NUDIX domain-containing protein

mru0170 NUDIX domain-containing protein

mru0737 NUDIX domain-containing protein

mru0148 oxidoreductase aldo/keto reductase family

mru0354 oxidoreductase aldo/keto reductase family

mru0416 oxidoreductase aldo/keto reductase family

mru0579 oxidoreductase aldo/keto reductase family

mru0773 oxidoreductase aldo/keto reductase family

mru0932 oxidoreductase aldo/keto reductase family

mru1120 oxidoreductase aldo/keto reductase family

mru1747 oxidoreductase GFO/IDH/MOCA family

mru0594 phosphodiesterase MJ0936 family

mru1623 phosphodiesterase MJ0936 family

mru0845 pyridoxal phosphate enzyme

mru0568 radical SAM domain-containing protein

mru0646 radical SAM domain-containing protein

mru1453 radical SAM domain-containing protein

mru1675 radical SAM domain-containing protein

mru2028 radical SAM domain-containing protein

mru2152 radical SAM domain-containing protein

mru0195 SAM dependent methyltransferase

mru0779 SAM dependent methyltransferase

mru0925 SAM dependent methyltransferase

mru0637 SAM-dependent methyltransferase

mru0933 SAM-dependent methyltransferase

mru0934 SAM-dependent methyltransferase

mru0935 SAM-dependent methyltransferase

mru1001 SAM-dependent methyltransferase

mru1011 SAM-dependent methyltransferase

mru1026 SAM-dependent methyltransferase

mru1520 SAM-dependent methyltransferase

mru1613 SAM-dependent methyltransferase

mru1643 SAM-dependent methyltransferase

mru0443 short-chain dehydrogenase family protein

mru1958 short-chain dehydrogenase family protein

**Other**

mru1443 ABM family protein

mru1254 ACT domain-containing protein

mru1788 ACT domain-containing protein

mru1728 AMMECR1 domain-containing protein

mru1231 ATP-binding protein

mru0709 ATP-grasp domain-containing protein

mru0598 band 7 family protein

mru1019 C_GCAxxG_C_C family protein

mru1611 C_GCAxxG_C_C family protein

mru0230 CAAX amino terminal protease family protein

mru0231 CAAX amino terminal protease family protein

mru0237 CAAX amino terminal protease family protein

mru0547 CAAX amino terminal protease family protein

mru1738 CAAX amino terminal protease family protein

mru0665 CBS domain-containing protein

mru0823 CBS domain-containing protein

mru1390 CBS domain-containing protein

mru1952 CBS domain-containing protein

mru1993 CBS domain-containing protein

mru1994 CBS domain-containing protein

mru2030 CBS domain-containing protein

mru0566 cytidyltransferase-related domain-containing protein

mru1982 DGC domain-containing protein

mru1308 FHA domain-containing protein

mru0221 Fic family protein

mru0392 GTP-binding protein

mru1729 GTP-binding protein

mru0474 HD domain-containing protein

mru1274 HD domain-containing protein

mru0194 HEAT repeat-containing protein

mru0601 HEAT repeat-containing protein

mru1034 HEAT repeat-containing protein

mru0095 isoprenylcysteine carboxyl methyltransferase family protein

mru1194 LemA family protein

mru1767 Met-10+ like-protein

mru1976 Met-10+ like-protein

mru0668 methanogenesis marker protein 1

mru1929 methanogenesis marker protein 10

mru0097 methanogenesis marker protein 11

mru2109 methanogenesis marker protein 12

mru0181 methanogenesis marker protein 13

mru1915 methanogenesis marker protein 14

mru1771 methanogenesis marker protein 15

mru1934 methanogenesis marker protein 16

mru1770 methanogenesis marker protein 17

mru1778 methanogenesis marker protein 2

mru1774 methanogenesis marker protein 3

mru1004 methanogenesis marker protein 4

mru1772 methanogenesis marker protein 5

mru1773 methanogenesis marker protein 6

mru1931 methanogenesis marker protein 7

mru0436 methanogenesis marker protein 8

mru1851 methanogenesis marker protein 9

mru0531 NIF3 family protein

mru1009 PHP domain-containing protein

mru2157 PHP domain-containing protein

mru1250 PIN domain-containing protein

mru0562 PP-loop family protein

mru0666 PP-loop family protein

mru1956 PP-loop family protein

mru1913 PRC-barrel domain-containing protein

mru0191 pyridoxamine 5'-phosphate oxidase family protein

mru0228 pyridoxamine 5'-phosphate oxidase family protein

mru1678 redox-active disulfide protein

mru1848 TfoX C-terminal domain-containing protein

mru0517 TfoX N-terminal domain-containing protein

mru0667 TfuA-like protein

mru1756 thioesterase family protein

mru1145 toxic anion resistance protein

mru1950 TraB family protein

mru0561 von Willebrand factor type A domain-containing protein

mru1593 von Willebrand factor type A domain-containing protein

mru1304 WD40 repeat-containing protein

mru1251 xylose isomerase-like TIM barrel domain-containing protein

mru1252 xylose isomerase-like TIM barrel domain-containing protein

mru2162 xylose isomerase-like TIM barrel domain-containing protein

mru0659 YhgE/Pip-like protein

mru1510 YhgE/Pip-like protein

mru0627 ZPR1 zinc-finger domain-containing protein

*************************************

**VITAMINS AND COFACTORS**

**Biotin**

mru2041 6-carboxyhexanoate-CoA ligase BioW

mru2042 8-amino-7-oxononanoate synthase BioF

mru2084 adenosylmethionine-8-amino-7-oxononanoate aminotransferase BioA

mru2087 biotin synthase BioB1

mru0527 biotin synthase BioB2

mru0846 biotin-acetyl-CoA-carboxylase ligase BirA

mru2086 dethiobiotin synthetase BioD

**Cobalamin**

mru1218 adenosylcobinamide amidohydrolase CbiZ

mru1891 alpha-ribazole phosphatase CobZ

mru2218 cobalamin biosynthesis protein CbiB

mru0887 cobalamin biosynthesis protein CbiD

mru0889 cobalamin biosynthesis protein CbiG

mru0539 cobalamin biosynthesis protein CbiM1

mru0885 cobalamin biosynthesis protein CbiM2

mru2200 cobalamin biosynthesis protein CbiX

mru1892 cobalamin-5-phosphate synthase CobS

mru0882 cobalt ABC transporter ATP-binding protein CbiO1

mru1217 cobalt ABC transporter ATP-binding protein CbiO2

mru1220 cobalt ABC transporter ATP-binding protein CbiO3

mru0541 cobalt ABC transporter permease protein CbiQ1

mru0883 cobalt ABC transporter permease protein CbiQ2

mru1221 cobalt ABC transporter permease protein CbiQ3

mru0895 cobalt chelatase CbiK

mru0540 cobalt transport protein CbiN1

mru0884 cobalt transport protein CbiN2

mru1638 cobyric acid synthase CbiP

mru0360 cobyrinic acid a,c-diamide synthase CbiA1

mru0371 cobyrinic acid a,c-diamide synthase CbiA2

mru0893 cobyrinic acid a,c-diamide synthase CbiA3

mru2151 cobyrinic acid a,c-diamide synthase CbiA4

mru1560 delta-aminolevulinic acid dehydratase HemB

mru0999 glutamate-1-semialdehyde-2,1-aminomutase HemL

mru1853 glutamyl-tRNA reductase HemA

mru1914 GTP:adenosylcobinamide-phosphate guanylyltransferase CobY

mru2047 magnesium chelatase H subunit BchH

mru1543 magnesium-protoporphyrin IX monomethyl ester anaerobic oxidative cyclase BchE

mru2101 nicotinate-nucleotide-dimethylbenzimidazole phosphoribosyltransferase CobT

mru1746 porphobilinogen deaminase HemC

mru0886 precorrin-2 C20-methyltransferase CbiL

mru2210 precorrin-3B C17-methyltransferase CbiH

mru0890 precorrin-3B C17-methyltransferase CbiH1

mru0888 precorrin-4 C11-methyltransferase CbiF

mru0891 precorrin-6x reductase CbiJ

mru1276 precorrin-6Y C5,15- methyltransferase (decarboxylating) CbiT

mru0892 precorrin-6Y C5,15-methyltransferase (decarboxylating) CbiET

mru0894 precorrin-8X methylmutase CbiC

mru1852 siroheme synthase CysG

mru1541 uroporphyrin-III C-methyltransferase CobA

mru1544 uroporphyrinogen-III synthase HemD

**Coenzyme B**

mru0384 homoaconitase large subunit AksD

mru1689 homoaconitase small subunit AksE

mru0385 homocitrate synthase AksA

mru1033 isohomocitrate dehydrogenase AksF

**Coenzyme F_420_**

mru0953 2-phospho-L-lactate guanylyltransferase CofC

mru1253 coenzyme F390 synthetase FtsA1

mru1787 coenzyme F390 synthetase FtsA2

mru0479 F_420_-0:gamma-glutamyl ligase

mru1842 F_420_-0:gamma-glutamyl ligase CofE

mru1974 FO synthase subunit 1 CofG

mru1266 FO synthase subunit 2 CofH

mru2213 fuculose 1-phosphate aldolase FucA

mru0672 lactaldehyde dehydrogenase CofA

mru1844 LPPG:FO 2-phospho-L-lactate transferase CofD

**Coenzyme M**

mru1949 2-phosphosulfolactate phosphatase ComB

mru1980 L-sulfolactate dehydrogenase ComC

**Glutathione**

mru0465 bifunctional glutamate-cysteine ligase/glutathione synthetase gshF

mru0463 gamma-glutamylcysteine synthetase GshA1

mru0464 gamma-glutamylcysteine synthetase GshA2

mru0462 glutamine amidotransferase

mru1935 glutathione peroxidase GpxA

mru0472 glutathione-disulfide reductase Gor1

mru1377 glutathione-disulfide reductase Gor2

**Metal-binding pterin**

mru0348 molybdate ABC transporter ATP-binding protein ModC

mru0200 molybdate ABC transporter permease protein ModB

mru0201 molybdate ABC transporter substrate-binding protein ModA

mru2137 molybdate transport system regulatory protein ModE

mru1782 molybdenum cofactor biosynthesis protein B MoaB

mru1680 molybdenum cofactor biosynthesis protein C MoaC

mru1691 molybdenum cofactor biosynthesis protein MoaA

mru1268 molybdenum cofactor biosynthesis protein MoaE

mru1269 molybdenum-pterin binding protein Mop1

mru1270 molybdenum-pterin binding protein Mop2

mru1271 molybdenum-pterin binding protein Mop3

mru1272 molybdenum-pterin binding protein Mop4

mru1273 molybdenum-pterin binding protein Mop5

mru0128 molybdopterin biosynthesis protein MoeA1

mru1870 molybdopterin biosynthesis protein MoeA2

mru0353 molybdopterin biosynthesis protein MoeB

mru0337 molybdopterin cofactor biosynthesis protein A MobA1

mru1277 molybdopterin-guanine dinucleotide biosynthesis protein A MobA2

mru0335 molybdopterin-guanine dinucleotide biosynthesis protein B MobB

**Methanofuran**

mru1896 L-tyrosine decarboxylase MfnA

**Methanopterin**

mru1690 beta-ribofuranosylaminobenzene 5'-phosphate synthase MptG

mru1962 GTP cyclohydrolase MptA

mru1283 creatinine amidohydrolase ArfB

mru1559 ATP:dephospho-CoA triphosphoribosyl transferase CitG

**Nicotinate**

mru0189 ATP-NAD kinase

mru0675 L-aspartate dehydrogenase

mru1430 NAD+ synthetase NadE

mru1704 NADH pyrophosphatase NudC

mru1267 nicotinamide-nucleotide adenylyltransferase

mru1750 nicotinate phosphoribosyltransferase

mru0618 nicotinate-nucleotide pyrophosphorylase NadC

mru0615 quinolinate synthetase A protein NadA

**Others**

mru0734 5-formyltetrahydrofolate cyclo-ligase

mru0557 dihydropteroate synthase-related protein

mru1912 FeS assembly ATPase SufC

mru1911 FeS assembly protein SufBD

mru1769 nitrogenase cofactor biosynthesis protein NifB

mru0466 dinitrogenase iron-molybdenum cofactor biosynthesis protein

**Pantothenate and coenzyme A**

mru1320 2-dehydropantoate 2-reductase PanE

mru1989 coenzyme A biosynthesis bifunctional protein CoaBC

mru1224 dephospho-CoA kinase CoaE

mru1010 pantothenate kinase CoaA

mru1225 pantothenate synthase PanC

mru0829 phosphopantetheine adenylyltransferase CoaD

**Riboflavin**

mru0089 3,4-dihydroxy-2-butanone 4-phosphate synthase RibB

mru0098 6,7-dimethyl-8- ribityllumazine synthase RibH

mru1007 diaminohydroxyphosphoribosylaminopyrimidine reductase RibD

mru1845 GTP cyclohydrolase III ArfA

mru2174 riboflavin kinase RibK

mru1215 riboflavin synthase RibC

**Thiamine**

mru1568 cysteine desulfurase NifS

mru1819 hydroxyethylthiazole kinase ThiM

mru0198 hydroxymethylpyrimidine transporter CytX

mru0199 phosphomethylpyrimidine kinase ThiD1

mru0952 phosphomethylpyrimidine kinase ThiD2

mru0494 thiamine biosynthesis ATP pyrophosphatase ThiI

mru0247 thiamine biosynthesis protein ThiC1

mru0444 thiamine biosynthesis protein ThiC2

mru0563 thiamine biosynthesis protein ThiS

mru1820 thiamine monophosphate synthase ThiE

mru2193 thiamine monphosphate kinase ThiL

mru0227 ThiF family protein

mru0532 ThiF family protein

**Ubiquinone**

mru0622 2-polyprenylphenol 6- hydroxylase UbiB1

mru0746 2-polyprenylphenol 6- hydroxylase UbiB2

mru1578 2-polyprenylphenol 6- hydroxylase UbiB3

mru1969 2-polyprenylphenol 6- hydroxylase UbiB4

mru1275 3-polyprenyl-4-hydroxybenzoate decarboxylase UbiX

mru0897 SAM-dependent methyltransferase UbiE family

mru2187 UbiD family decarboxylase

*************************************
