# Supplementary material for: The Genome Sequence of the Rumen Methanogen Methanobrevibacter ruminantium Reveals New Possibilities for Controlling Ruminant Methane Emissions
Source: PLoS One. 2010 Jan 28;5(1):e8926. doi: 10.1371/journal.pone.0008926 (PMC2812497; doi:10.1371/journal.pone.0008926)
Supplement: Table S2 — HGT analysis. (0.46 MB DOC) [file pone.0008926.s002.doc]

| **Table S2**. M1 genes postulated to have originated from horizontal gene transfer events based on analysis by the Darkhorse [S5] algorithm. Hits with a lineage probability score (LPI) of less than 0.6 are shown. | | | | | | | |
| --- | --- | --- | --- | --- | --- | --- | --- |
| **Locus_tag** | **Sequin Annotation** | **Functional Classification** | **Sub Classification** | **LPI** | **E-value** | **Species** | **Lineage** |
|  |  |  |  |  |  |  |  |
| mru2096 | cysteine synthase CysKM2 | Amino acid biosynthesis | Cysteine | 0.134 | 7E-65 | *Clostridium leptum* DSM 753 | Bacteria; Firmicutes |
| mru0810 | glutamate synthase domain-containing protein | Amino acid biosynthesis | glutamate/glutamine | 0.13 | 2E-177 | *Anaerofustis stercorihominis* DSM 17244 | Bacteria; Firmicutes |
| mru0213 | tryptophan synthase beta subunit TrpB1 | Amino acid biosynthesis | Tryptophan | 0.134 | 1E-179 | *Clostridium thermocellum* ATCC 27405 | Bacteria; Firmicutes |
| mru1667 | HIRAN domain-containing protein | Cell cycle | Chromosome replication | 0.138 | 0.00000001 | *Anaerococcus prevotii* DSM 20548 | Bacteria; Firmicutes |
| mru1158 | RecF/RecN/SMC N terminal domain-containing protein | Cell cycle | Genome segregation | 0.088 | 4E-13 | *Desulfatibacillum alkenivorans* AK-01 | Bacteria; Proteobacteria |
| mru0004 | adhesin-like protein | Cell envelope | Cell surface proteins | 0.003 | 1E-22 | *Trypanosoma brucei* TREU927 | Eukaryota; Kinetoplastida |
| mru1299 | adhesin-like protein | Cell envelope | Cell surface proteins | 0.003 | 2E-36 | *Trichoplax adhaerens* | Eukaryota; Metazoa |
| mru0687 | adhesin-like protein | Cell envelope | Cell surface proteins | 0.082 | 1E-25 | *Planctomyces maris* DSM 8797 | Bacteria; Planctomycetes |
| mru1263 | adhesin-like protein | Cell envelope | Cell surface proteins | 0.082 | 2E-22 | *Planctomyces maris* DSM 8797 | Bacteria; Planctomycetes |
| mru1417 | adhesin-like protein | Cell envelope | Cell surface proteins | 0.082 | 1E-10 | *Planctomyces maris* DSM 8797 | Bacteria; Planctomycetes |
| mru2178 | adhesin-like protein | Cell envelope | Cell surface proteins | 0.085 | 5E-96 | *Polaribacter sp.* MED152 | Bacteria; Bacteroidetes |
| mru1312 | adhesin-like protein | Cell envelope | Cell surface proteins | 0.088 | 2E-17 | *Chloroflexus aurantiacus* J-10-fl | Bacteria; Chloroflexi |
| mru0036 | adhesin-like protein | Cell envelope | Cell surface proteins | 0.112 | 8E-33 | *Lactobacillus johnsonii* NCC 533 | Bacteria; Firmicutes |
| mru1315 | adhesin-like protein | Cell envelope | Cell surface proteins | 0.112 | 2E-13 | *Listeria monocytogenes* str. 4b H7858 | Bacteria; Firmicutes |
| mru2134 | adhesin-like protein | Cell envelope | Cell surface proteins | 0.13 | 6E-165 | *Coprococcus eutactus* ATCC 27759 | Bacteria; Firmicutes |
| mru1051 | UDP-glucose/GDP-mannose dehydrogenase | Cell envelope | Exopolysaccharides | 0.084 | 3E-146 | *Bifidobacterium adolescentis* L2-32 | Bacteria; Actinobacteria |
| mru0099 | glycosyl transferase GT4 family | Cell envelope | Exopolysaccharides | 0.087 | 8E-62 | *Dinoroseobacter shibae* DFL 12 | Bacteria; Proteobacteria |
| mru1074 | glycosyl transferase GT2 family | Cell envelope | Exopolysaccharides | 0.087 | 0.000000001 | *Hydrogenobaculum sp*. Y04AAS1 | Bacteria; Aquificae |
| mru1527 | glycosyl transferase | Cell envelope | Exopolysaccharides | 0.087 | 2E-61 | *Bacteroides fragilis* NCTC 9343 | Bacteria; Bacteroidetes |
| mru1879 | sialyltransferase | Cell envelope | Exopolysaccharides | 0.087 | 3E-54 | *Hahella chejuensis* KCTC 2396 | Bacteria; Proteobacteria |
| mru1528 | glycosyl transferase | Cell envelope | Exopolysaccharides | 0.134 | 2E-55 | *Clostridium botulinum* A2 str. Kyoto | Bacteria; Firmicutes |
| mru1876 | CMP-N-acetylneuraminic acid synthetase NeuA | Cell envelope | Exopolysaccharides | 0.134 | 0 | *Clostridium botulinum* A3 str. Loch Maree | Bacteria; Firmicutes |
| mru1878 | N-acetyl neuramic acid synthetase NeuB | Cell envelope | Exopolysaccharides | 0.134 | 1E-124 | *Clostridium botulinum* B1 str. Okra | Bacteria; Firmicutes |
| mru2183 | glycosyl transferase GT2 family | Cell envelope | Exopolysaccharides | 0.134 | 5E-40 | *Clostridium sp*. SS2/1 | Bacteria; Firmicutes |
| mru1075 | UDP-glucose/GDP-mannose dehydrogenase | Cell envelope | Exopolysaccharides | 0.138 | 3E-139 | *Anaerococcus prevotii* DSM 20548 | Bacteria; Firmicutes |
| mru2181 | glycosyl transferase GT2 family/CDP-glycerol:poly(glycerophosphate) glycerophosphotransferase | Cell envelope | Other | 0.134 | 2E-129 | *Clostridium sp.* SS2/1 | Bacteria; Firmicutes |
| mru2182 | glycosyl transferase GT2 family/CDP-glycerol:poly(glycerophosphate) glycerophosphotransferase | Cell envelope | Other | 0.134 | 2E-127 | *Clostridium sp.* SS2/1 | Bacteria; Firmicutes |
| mru2191 | CDP-glycerol:poly(glycerophosphate) glycerophosphotransferase | Cell envelope | Other | 0.134 | 5E-123 | *Clostridium sp*. SS2/1 | Bacteria; Firmicutes |
| mru1260 | NADPH-dependent FMN reductase | Cellular processes | Electron transport | 0.088 | 3E-43 | *Treponema denticola* ATCC 35405 | Bacteria; Spirochaetes |
| mru1369 | NADPH-dependent FMN reductase | Cellular processes | Electron transport | 0.129 | 2E-24 | *Heliobacterium modesticaldum* Ice1 | Bacteria; Firmicutes |
| mru0580 | NADPH-dependent FMN reductase | Cellular processes | Electron transport | 0.134 | 4E-72 | *Clostridium leptum* DSM 753 | Bacteria; Firmicutes |
| mru1732 | NADPH-dependent FMN reductase | Cellular processes | Electron transport | 0.134 | 2E-55 | *Clostridium beijerinckii* NCIMB 8052 | Bacteria; Firmicutes |
| mru1609 | NADPH-dependent FMN reductase | Cellular processes | Electron transport | 0.141 | 2E-76 | *Clostridiales bacterium* 1_7_47_FAA | Bacteria; Firmicutes |
| mru1258 | rubredoxin Rub2 | Cellular processes | Oxidative stress response | 0.088 | 1E-13 | *Syntrophus aciditrophicus* SB | Bacteria; Proteobacteria |
| mru1367 | rubrerythrin Rbr2 | Cellular processes | Oxidative stress response | 0.093 | 4E-65 | *Slackia heliotrinireducens* DSM 20476 | Bacteria; Actinobacteria |
| mru1259 | rubredoxin Rub3 | Cellular processes | Oxidative stress response | 0.13 | 3E-11 | *Oribacterium sinus* F0268 | Bacteria; Firmicutes |
| mru1564 | desulfoferrodoxin Dfx | Cellular processes | Oxidative stress response | 0.138 | 2E-31 | *Anaerocellum thermophilum* DSM 6725 | Bacteria; Firmicutes |
| mru1519 | pyruvate-formate lyase Pfl | Central carbon metabolism | Formate | 0.129 | 2E-54 | *Desulfitobacterium hafniense* Y51 | Bacteria; Firmicutes |
| mru0635 | pyruvate kinase PykA | Central carbon metabolism | Gluconeogenesis | 0.138 | 6E-103 | *Anaerocellum thermophilum* DSM 6725 | Bacteria; Firmicutes |
| mru1310 | 3-hexulose-6-phosphate isomerase Phi2 | Central carbon metabolism | RUMP pathway | 0.424 | 4E-23 | *Staphylothermus marinus* F1 | Archaea; Crenarchaeota |
| mru0155 | iron-sulfur cluster binding protein | Energy metabolism | Electron transport | 0.13 | 4E-35 | *Dorea longicatena* DSM 13814 | Bacteria; Firmicutes |
| mru1711 | 4Fe-4S binding domain-containing protein | Energy metabolism | Electron transport | 0.13 | 2E-40 | *Dorea longicatena* DSM 13814 | Bacteria; Firmicutes |
| mru0009 | flavodoxin domain containing protein | Energy metabolism | Electron transport | 0.134 | 5E-54 | *Clostridium kluyveri* NBRC 12016 | Bacteria; Firmicutes |
| mru2036 | 4Fe-4S binding domain-containing protein | Energy metabolism | Electron transport | 0.134 | 2E-87 | *Clostridium perfringens* CPE str. F4969 | Bacteria; Firmicutes |
| mru0065 | NADP-dependent alcohol dehydrogenase Adh1 | Energy metabolism | Ethanol | 0.134 | 6E-112 | *Clostridium sporogenes* ATCC 15579 | Bacteria; Firmicutes |
| mru1297 | hypothetical protein | Hypothetical | Conserved | 0.003 | 2E-110 | *Ralstonia* phage RSL1 | Viruses; Caudovirales |
| mru0643 | hypothetical protein | Hypothetical | Conserved | 0.081 | 2E-26 | *Mycoplasma hyopneumoniae* J | Bacteria; Tenericutes |
| mru0899 | hypothetical protein | Hypothetical | Conserved | 0.081 | 1E-13 | *Chlorobium chlorochromatii* CaD3 | Bacteria; Chlorobi |
| mru0751 | hypothetical protein | Hypothetical | Conserved | 0.085 | 8E-11 | *Corynebacterium urealyticum* DSM 7109 | Bacteria; Actinobacteria |
| mru0945 | hypothetical protein | Hypothetical | Conserved | 0.085 | 1E-20 | *Psychroflexus torquis* ATCC 700755 | Bacteria; Bacteroidetes |
| mru1579 | hypothetical protein | Hypothetical | Conserved | 0.085 | 1E-33 | *Geodermatophilus obscurus* DSM 43160 | Bacteria; Actinobacteria |
| mru1964 | hypothetical protein | Hypothetical | Conserved | 0.085 | 8E-57 | *Actinomyces odontolyticus* ATCC 17982 | Bacteria; Actinobacteria |
| mru2185 | hypothetical protein | Hypothetical | Conserved | 0.085 | 0.000003 | *Flavobacterium psychrophilum* JIP02/86 | Bacteria; Bacteroidetes |
| mru0021 | hypothetical protein | Hypothetical | Conserved | 0.087 | 8E-47 | *Bacteroides dorei* DSM 17855 | Bacteria; Bacteroidetes |
| mru0134 | hypothetical protein | Hypothetical | Conserved | 0.087 | 2E-35 | *Tolumonas auensis* DSM 9187 | Bacteria; Proteobacteria |
| mru0223 | hypothetical protein | Hypothetical | Conserved | 0.087 | 2E-29 | *Dichelobacter nodosus* | Bacteria; Proteobacteria |
| mru0644 | hypothetical protein | Hypothetical | Conserved | 0.087 | 4E-13 | *Roseobacter* sp. AzwK-3b | Bacteria; Proteobacteria |
| mru0836 | hypothetical protein | Hypothetical | Conserved | 0.087 | 9E-14 | *Roseobacter* sp. AzwK-3b | Bacteria; Proteobacteria |
| mru1073 | hypothetical protein | Hypothetical | Conserved | 0.087 | 5E-15 | *Opitutus terrae* PB90-1 | Bacteria; Verrucomicrobia |
| mru1290 | hypothetical protein | Hypothetical | Conserved | 0.087 | 1E-13 | *Hahella chejuensis* KCTC 2396 | Bacteria; Proteobacteria |
| mru1932 | hypothetical protein | Hypothetical | Conserved | 0.087 | 1E-132 | *Janthinobacterium* sp. Marseille | Bacteria; Proteobacteria |
| mru1966 | hypothetical protein | Hypothetical | Conserved | 0.087 | 5E-10 | *Bacteroides cellulosilyticus* DSM 14838 | Bacteria; Bacteroidetes |
| mru0028 | hypothetical protein | Hypothetical | Conserved | 0.088 | 5E-11 | *Desulfovibrio vulgaris* str. Miyazaki F | Bacteria; Proteobacteria |
| mru0118 | hypothetical protein | Hypothetical | Conserved | 0.088 | 2E-28 | *Leptotrichia buccalis* DSM 1135 | Bacteria; Fusobacteria |
| mru0573 | hypothetical protein | Hypothetical | Conserved | 0.088 | 1E-12 | *Syntrophobacter fumaroxidans* MPOB | Bacteria; Proteobacteria |
| mru0780 | hypothetical protein | Hypothetical | Conserved | 0.088 | 0.0000002 | *Rhizobium etli* CIAT 652 | Bacteria; Proteobacteria |
| mru0803 | hypothetical protein | Hypothetical | Conserved | 0.088 | 5E-34 | *Nitrobacter sp*. Nb-311A | Bacteria; Proteobacteria |
| mru1063 | hypothetical protein | Hypothetical | Conserved | 0.088 | 1E-28 | *Rhodopseudomonas palustris* HaA2 | Bacteria; Proteobacteria |
| mru1172 | hypothetical protein | Hypothetical | Conserved | 0.088 | 0.0000002 | *Rhizobium leguminosarum bv. trifolii* WSM1325 | Bacteria; Proteobacteria |
| mru1749 | hypothetical protein | Hypothetical | Conserved | 0.088 | 1E-45 | *Desulfovibrio salexigens* DSM 2638 | Bacteria; Proteobacteria |
| mru2194 | hypothetical protein | Hypothetical | Conserved | 0.088 | 1E-55 | *Geobacter bemidjiensis* Bem | Bacteria; Proteobacteria |
| mru1389 | hypothetical protein | Hypothetical | Conserved | 0.096 | 0.0000002 | *Microcoleus chthonoplastes* PCC 7420 | Bacteria; Cyanobacteria |
| mru0642 | hypothetical protein | Hypothetical | Conserved | 0.108 | 1E-31 | *Mollicutes bacterium* D7 | Bacteria; Tenericutes |
| mru0745 | hypothetical protein | Hypothetical | Conserved | 0.112 | 8E-11 | *Staphylococcus carnosus* subsp. *carnosus* TM300 | Bacteria; Firmicutes |
| mru1886 | hypothetical protein | Hypothetical | Conserved | 0.112 | 2E-30 | *Brevibacillus brevis* NBRC 100599 | Bacteria; Firmicutes |
| mru1937 | hypothetical protein | Hypothetical | Conserved | 0.112 | 7E-43 | *Lactobacillus vaginalis* ATCC 49540 | Bacteria; Firmicutes |
| mru1242 | hypothetical protein | Hypothetical | Conserved | 0.117 | 2E-24 | *Eubacterium biforme* DSM 3989 | Bacteria; Firmicutes |
| mru2130 | hypothetical protein | Hypothetical | Conserved | 0.117 | 5E-112 | *Eubacterium biforme* DSM 3989 | Bacteria; Firmicutes |
| mru0791 | hypothetical protein | Hypothetical | Conserved | 0.12 | 8E-10 | *Coprothermobacter proteolyticus* DSM 5265 | Bacteria; Firmicutes |
| mru0078 | hypothetical protein | Hypothetical | Conserved | 0.129 | 2E-100 | *Veillonella parvula* DSM 2008 | Bacteria; Firmicutes |
| mru0776 | hypothetical protein | Hypothetical | Conserved | 0.129 | 3E-80 | *Dethiobacter alkaliphilus* AHT 1 | Bacteria; Firmicutes |
| mru0785 | hypothetical protein | Hypothetical | Conserved | 0.129 | 9E-81 | *Dethiobacter alkaliphilus* AHT 1 | Bacteria; Firmicutes |
| mru1229 | hypothetical protein | Hypothetical | Conserved | 0.129 | 1E-24 | *Desulfotomaculum acetoxidans* DSM 771 | Bacteria; Firmicutes |
| mru1323 | hypothetical protein | Hypothetical | Conserved | 0.129 | 4E-23 | *Desulfitobacterium hafniense* Y51 | Bacteria; Firmicutes |
| mru1724 | hypothetical protein | Hypothetical | Conserved | 0.129 | 2E-23 | *Desulfotomaculum reducens* MI-1 | Bacteria; Firmicutes |
| mru1967 | hypothetical protein | Hypothetical | Conserved | 0.129 | 2E-33 | *Ruminococcus gnavus* ATCC 29149 | Bacteria; Firmicutes |
| mru2143 | hypothetical protein | Hypothetical | Conserved | 0.129 | 1E-39 | *Ruminococcus obeum* ATCC 29174 | Bacteria; Firmicutes |
| mru0100 | hypothetical protein | Hypothetical | Conserved | 0.13 | 1E-43 | *Roseburia intestinalis* L1-82 | Bacteria; Firmicutes |
| mru0156 | hypothetical protein | Hypothetical | Conserved | 0.13 | 3E-47 | *Anaerofustis stercorihominis* DSM 17244 | Bacteria; Firmicutes |
| mru0185 | hypothetical protein | Hypothetical | Conserved | 0.13 | 4E-112 | *Anaerofustis stercorihominis* DSM 17244 | Bacteria; Firmicutes |
| mru0558 | hypothetical protein | Hypothetical | Conserved | 0.13 | 0.0000004 | *Anaerofustis stercorihominis* DSM 17244 | Bacteria; Firmicutes |
| mru0753 | hypothetical protein | Hypothetical | Conserved | 0.13 | 8E-112 | *Dorea longicatena* DSM 13814 | Bacteria; Firmicutes |
| mru0806 | hypothetical protein | Hypothetical | Conserved | 0.13 | 1E-56 | *Dorea formicigenerans* ATCC 27755 | Bacteria; Firmicutes |
| mru1065 | hypothetical protein | Hypothetical | Conserved | 0.13 | 1E-32 | *Roseburia intestinalis* L1-82 | Bacteria; Firmicutes |
| mru1227 | hypothetical protein | Hypothetical | Conserved | 0.13 | 0.000008 | *Coprococcus eutactus* ATCC 27759 | Bacteria; Firmicutes |
| mru1287 | hypothetical protein | Hypothetical | Conserved | 0.13 | 1E-24 | *Eubacterium siraeum* DSM 15702 | Bacteria; Firmicutes |
| mru1938 | hypothetical protein | Hypothetical | Conserved | 0.13 | 4E-44 | *Coprococcus eutactus* ATCC 27759 | Bacteria; Firmicutes |
| mru0047 | hypothetical protein | Hypothetical | Conserved | 0.134 | 3E-44 | *Clostridium hiranonis* DSM 13275 | Bacteria; Firmicutes |
| mru0150 | hypothetical protein | Hypothetical | Conserved | 0.134 | 6E-39 | *Clostridium botulinum* E3 str. Alaska E43 | Bacteria; Firmicutes |
| mru0167 | hypothetical protein | Hypothetical | Conserved | 0.134 | 3E-29 | *Clostridium scindens* ATCC 35704 | Bacteria; Firmicutes |
| mru0186 | hypothetical protein | Hypothetical | Conserved | 0.134 | 9E-158 | *Clostridium acetobutylicum* ATCC 824 | Bacteria; Firmicutes |
| mru0572 | hypothetical protein | Hypothetical | Conserved | 0.134 | 3E-32 | *Clostridium* sp. M62/1 | Bacteria; Firmicutes |
| mru0947 | hypothetical protein | Hypothetical | Conserved | 0.134 | 4E-136 | *Clostridium hiranonis* DSM 13275 | Bacteria; Firmicutes |
| mru1173 | hypothetical protein | Hypothetical | Conserved | 0.134 | 5E-30 | *Clostridium thermocellum* ATCC 27405 | Bacteria; Firmicutes |
| mru1177 | hypothetical protein | Hypothetical | Conserved | 0.134 | 3E-42 | *Clostridium thermocellum* ATCC 27405 | Bacteria; Firmicutes |
| mru1311 | hypothetical protein | Hypothetical | Conserved | 0.134 | 2E-122 | *Clostridium thermocellum* ATCC 27405 | Bacteria; Firmicutes |
| mru1330 | hypothetical protein | Hypothetical | Conserved | 0.134 | 1E-14 | *Clostridium asparagiforme* DSM 15981 | Bacteria; Firmicutes |
| mru2085 | hypothetical protein | Hypothetical | Conserved | 0.134 | 5E-53 | *Clostridium hylemonae* DSM 15053 | Bacteria; Firmicutes |
| mru2154 | hypothetical protein | Hypothetical | Conserved | 0.134 | 0.00000002 | *Clostridium beijerinckii* NCIMB 8052 | Bacteria; Firmicutes |
| mru0835 | hypothetical protein | Hypothetical | Conserved | 0.138 | 1E-33 | *Blautia hydrogenotrophica* DSM 10507 | Bacteria; Firmicutes |
| mru0939 | hypothetical protein | Hypothetical | Conserved | 0.141 | 5E-38 | *Clostridiales bacterium* 1_7_47_FAA | Bacteria; Firmicutes |
| mru2205 | hypothetical protein | Hypothetical | Conserved | 0.424 | 0.00000004 | *Sulfolobus tokodaii* str. 7 | Archaea; Crenarchaeota |
| mru1521 | hypothetical protein | Hypothetical | Hypothetical | 0.086 | 0.00000002 | *Parabacteroides johnsonii* DSM 18315 | Bacteria; Bacteroidetes |
| mru0017 | hypothetical protein | Hypothetical | Hypothetical | 0.087 | 5E-21 | *Bacteroides* sp. D1 | Bacteria; Bacteroidetes |
| mru0018 | hypothetical protein | Hypothetical | Hypothetical | 0.087 | 0.000000003 | *Bacteroides* sp. D1 | Bacteria; Bacteroidetes |
| mru0053 | hypothetical protein | Hypothetical | Hypothetical | 0.087 | 2E-15 | *Bacteroides ovatus* ATCC 8483 | Bacteria; Bacteroidetes |
| mru0432 | hypothetical protein | Hypothetical | Hypothetical | 0.093 | 2E-100 | *Slackia heliotrinireducens* DSM 20476 | Bacteria; Actinobacteria |
| mru1582 | hypothetical protein | Hypothetical | Hypothetical | 0.093 | 0.0000004 | *Slackia heliotrinireducens* DSM 20476 | Bacteria; Actinobacteria |
| mru2173 | hypothetical protein | Hypothetical | Hypothetical | 0.112 | 0.0000002 | *Lactobacillus salivarius* ATCC 11741 | Bacteria; Firmicutes |
| mru1161 | hypothetical protein | Hypothetical | Hypothetical | 0.114 | 2E-10 | *Bacillus cereus* BGSC 6E1 | Bacteria; Firmicutes |
| mru0024 | hypothetical protein | Hypothetical | Hypothetical | 0.138 | 6E-26 | *Anaerococcus lactolyticus* ATCC 51172 | Bacteria; Firmicutes |
| mru0025 | hypothetical protein | Hypothetical | Hypothetical | 0.138 | 0.00000004 | *Anaerococcus lactolyticus* ATCC 51172 | Bacteria; Firmicutes |
| mru1031 | 3-oxoacyl-(acyl-carrier-protein) reductase FabG1 | Lipid metabolism | Bacterial | 0.112 | 3E-48 | *Paenibacillus sp*. JDR-2 | Bacteria; Firmicutes |
| mru1289 | diacylglycerol kinase DagK | Lipid metabolism | Bacterial | 0.134 | 7E-19 | *Clostridium scindens* ATCC 35704 | Bacteria; Firmicutes |
| mru1630 | 3-oxoacyl-(acyl-carrier-protein) reductase FabG2 | Lipid metabolism | Bacterial | 0.134 | 2E-74 | *Clostridium cellulolyticum* H10 | Bacteria; Firmicutes |
| mru1178 | CRISPR-associated protein TIGR02710 family | Mobile elements | CRISPR-associated genes | 0.088 | 1E-28 | *Syntrophus aciditrophicus* SB | Bacteria; Proteobacteria |
| mru0256 | phage integrase | Mobile elements | Prophage | 0.003 | 1E-11 | *Methanobacterium* phage psiM2 | Viruses; Caudovirales |
| mru0307 | phage-related protein | Mobile elements | Prophage | 0.003 | 3E-16 | *Methanothermobacter* phage psiM100 | Viruses; Caudovirales |
| mru0324 | type II restriction enzyme, methylase subunit | Mobile elements | Prophage | 0.085 | 5E-110 | *Robiginitalea biformata* HTCC2501 | Bacteria; Bacteroidetes |
| mru0282 | phage-related protein | Mobile elements | Prophage | 0.086 | 2E-16 | *Alistipes putredinis* DSM 17216 | Bacteria; Bacteroidetes |
| mru0285 | terminase large subunit | Mobile elements | Prophage | 0.087 | 4E-32 | *Burkholderia pseudomallei* Pasteur 52237 | Bacteria; Proteobacteria |
| mru0298 | hypothetical protein | Mobile elements | Prophage | 0.087 | 1E-13 | *Sulfurospirillum deleyianum* DSM 6946 | Bacteria; Proteobacteria |
| mru0267 | hypothetical protein | Mobile elements | Prophage | 0.111 | 9E-13 | *Ricinus communis* | Eukaryota; Viridiplantae |
| mru0280 | ParB-like nuclease domain-containing protein | Mobile elements | Prophage | 0.112 | 3E-19 | *Lactobacillus reuteri* CF48-3A | Bacteria; Firmicutes |
| mru0287 | phage portal protein | Mobile elements | Prophage | 0.113 | 1E-67 | *Geobacillus* sp. G11MC16 | Bacteria; Firmicutes |
| mru0308 | phage-related protein | Mobile elements | Prophage | 0.113 | 1E-40 | *Geobacillus* sp. G11MC16 | Bacteria; Firmicutes |
| mru0310 | phage-related protein | Mobile elements | Prophage | 0.113 | 0.000002 | *Geobacillus thermodenitrificans* NG80-2 | Bacteria; Firmicutes |
| mru0057 | phage-related protein | Mobile elements | Prophage | 0.114 | 7E-39 | *Bacillus coahuilensis* m4-4 | Bacteria; Firmicutes |
| mru0058 | phage-related protein | Mobile elements | Prophage | 0.114 | 0.000001 | *Bacillus coahuilensis* m4-4 | Bacteria; Firmicutes |
| mru0288 | phage-related protein | Mobile elements | Prophage | 0.114 | 2E-51 | *Bacillus coahuilensis* m4-4 | Bacteria; Firmicutes |
| mru0311 | phage-related protein | Mobile elements | Prophage | 0.114 | 2E-17 | *Bacillus coahuilensis* m4-4 | Bacteria; Firmicutes |
| mru0313 | phage-related protein | Mobile elements | Prophage | 0.114 | 0.0000003 | *Bacillus coahuilensis* m4-4 | Bacteria; Firmicutes |
| mru0321 | dnd system-associated protein 3 | Mobile elements | Prophage | 0.114 | 5E-107 | *Bacillus cereus* MM3 | Bacteria; Firmicutes |
| mru0322 | dnd system-associated protein 1 | Mobile elements | Prophage | 0.114 | 3E-73 | *Bacillus cereus* BDRD-ST196 | Bacteria; Firmicutes |
| mru0323 | dnd system-associated protein 2 | Mobile elements | Prophage | 0.114 | 0 | *Bacillus cereus* m1293 | Bacteria; Firmicutes |
| mru0299 | hypothetical protein | Mobile elements | Prophage | 0.13 | 2E-13 | *Anaerostipes caccae* DSM 14662 | Bacteria; Firmicutes |
| mru0296 | hypothetical protein | Mobile elements | Prophage | 0.138 | 2E-17 | *Blautia hansenii* DSM 20583 | Bacteria; Firmicutes |
| mru0094 | nitroreductase family protein | Nitrogen metabolism | General | 0.134 | 3E-38 | *Clostridium cellulolyticum* H10 | Bacteria; Firmicutes |
| mru0995 | nitroreductase family protein | Nitrogen metabolism | General | 0.134 | 3E-40 | *Clostridium* sp. M62/1 | Bacteria; Firmicutes |
| mru1580 | ADP-ribosylglycohydrolase family protein | Nitrogen metabolism | Other | 0.087 | 2E-64 | *Bacteroides caccae* ATCC 43185 | Bacteria; Bacteroidetes |
| mru0749 | ADP-ribosylglycohydrolase family protein | Nitrogen metabolism | Other | 0.097 | 1E-71 | *Synechococcus* sp. PCC 7002 | Bacteria; Cyanobacteria |
| mru1455 | ADP-ribosylglycohydrolase family protein | Nitrogen metabolism | Other | 0.097 | 8E-58 | *Synechococcus* sp. PCC 7002 | Bacteria; Cyanobacteria |
| mru1157 | helicase RecD/TraA family | Nucleic acid metabolism | Helicase | 0.087 | 3E-46 | *Bacteroides dorei* DSM 17855 | Bacteria; Bacteroidetes |
| mru0620 | helicase SNF2 family | Nucleic acid metabolism | Helicase | 0.088 | 1E-152 | *Wolbachia endosymbiont of Culex quinquefasciatus* JHB | Bacteria; Proteobacteria |
| mru0778 | DEAD/DEAH box helicase domain-containing protein | Nucleic acid metabolism | Helicase | 0.134 | 0 | *Clostridium butyricum* 5521 | Bacteria; Firmicutes |
| mru1429 | DNA mismatch endonuclease Vsr | Nucleic acid metabolism | Recombination and repair | 0.084 | 6E-49 | Bifidobacterium longum subsp. *infantis* ATCC 15697 | Bacteria; Actinobacteria |
| mru1575 | 6-O-methylguanine DNA methyltransferase Ogt | Nucleic acid metabolism | Recombination and repair | 0.088 | 3E-26 | *Syntrophus aciditrophicus* SB | Bacteria; Proteobacteria |
| mru1256 | excinuclease ABC A subunit UvrA2 | Nucleic acid metabolism | Recombination and repair | 0.109 | 0 | *Catenibacterium mitsuokai* DSM 15897 | Bacteria; Firmicutes |
| mru0813 | exodeoxyribonuclease VII small subunit XseB | Nucleic acid metabolism | Recombination and repair | 0.111 | 3E-12 | *Enterococcus faecium* DO | Bacteria; Firmicutes |
| mru0218 | uracil-DNA glycosylase Ung | Nucleic acid metabolism | Recombination and repair | 0.113 | 2E-71 | *Geobacillus kaustophilus* HTA426 | Bacteria; Firmicutes |
| mru0812 | exodeoxyribonuclease VII large subunit XseA | Nucleic acid metabolism | Recombination and repair | 0.114 | 3E-87 | *Bacillus* sp. SG-1 | Bacteria; Firmicutes |
| mru2068 | DNA-3-methyladenine glycosylase I Tag | Nucleic acid metabolism | Recombination and repair | 0.13 | 4E-66 | *Anaerofustis stercorihominis* DSM 17244 | Bacteria; Firmicutes |
| mru0770 | exonuclease | Nucleic acid metabolism | Recombination and repair | 0.138 | 2E-41 | *Blautia hansenii* DSM 20583 | Bacteria; Firmicutes |
| mru1165 | restriction enzyme methylase subunit | Nucleic acid metabolism | Restriction and modification | 0.097 | 0 | *Synechococcus* sp. PCC 7002 | Bacteria; Cyanobacteria |
| mru1166 | type II restriction endonuclease | Nucleic acid metabolism | Restriction and modification | 0.111 | 3E-119 | *Streptococcus equi* subsp. *equi* 4047 | Bacteria; Firmicutes |
| mru0027 | DNA-cytosine methyltransferase | Nucleic acid metabolism | Restriction and modification | 0.114 | 2E-70 | *Bacillus cereus* 03BB102 | Bacteria; Firmicutes |
| mru1167 | DNA modification methylase | Nucleic acid metabolism | Restriction and modification | 0.13 | 2E-128 | *Eubacterium hallii* DSM 3353 | Bacteria; Firmicutes |
| mru0026 | DNA-cytosine methyltransferase | Nucleic acid metabolism | Restriction and modification | 0.138 | 5E-91 | *Anaerococcus lactolyticus* ATCC 51172 | Bacteria; Firmicutes |
| mru0029 | 5-methylcytosine restriction system component protein | Nucleic acid metabolism | Restriction and modification | 0.138 | 9E-72 | *Bryantella formatexigens* DSM 14469 | Bacteria; Firmicutes |
| mru2021 | transglutaminase domain-containing protein | Protein fate | Protein degradation | 0.084 | 4E-42 | *Bifidobacterium breve* DSM 20213 | Bacteria; Actinobacteria |
| mru1305 | DnaK-related protein | Protein fate | Protein folding | 0.096 | 3E-78 | *Chthoniobacter flavus* Ellin428 | Bacteria; Verrucomicrobia |
| mru1812 | DnaK-related protein | Protein fate | Protein folding | 0.12 | 1E-100 | *Coprothermobacter proteolyticus* DSM 5265 | Bacteria; Firmicutes |
| mru1610 | rRNA methylase | Protein synthesis | Other | 0.087 | 1E-42 | *Thermosipho africanus* TCF52B | Bacteria; Thermotogae |
| mru0437 | queuosine biosynthesis protein QueC | Protein synthesis | RNA processing | 0.087 | 6E-45 | *Idiomarina loihiensis* L2TR | Bacteria; Proteobacteria |
| mru0439 | 7-cyano-7-deazaguanosine biosynthesis protein QueE | Protein synthesis | RNA processing | 0.087 | 4E-21 | *Erythrobacter sp.* SD-21 | Bacteria; Proteobacteria |
| mru0438 | queuosine biosynthesis protein QueD | Protein synthesis | RNA processing | 0.088 | 4E-11 | *Beijerinckia indica* subsp. *indica* ATCC 9039 | Bacteria; Proteobacteria |
| mru1025 | RNA ligase DRB0094 family | Protein synthesis | RNA processing | 0.09 | 8E-30 | *Chitinophaga pinensis* DSM 2588 | Bacteria; Bacteroidetes |
| mru0096 | tRNA-dihydrouridine synthase DusA1 | Protein synthesis | RNA processing | 0.117 | 3E-94 | *Clostridium ramosum* DSM 1402 | Bacteria; Firmicutes |
| mru1286 | CMP/dCMP deaminase | Purines & Pyrimidines | Pyrimidine interconversion | 0.111 | 2E-29 | *Abiotrophia defectiva* ATCC 49176 | Bacteria; Firmicutes |
| mru0576 | sugar fermentation stimulation protein SfsA1 | Regulation | Other | 0.096 | 4E-55 | *Dehalococcoides ethenogenes* 195 | Bacteria; Chloroflexi |
| mru0187 | carbon starvation protein CstA | Regulation | Other | 0.134 | 9E-126 | *Clostridium bartlettii* DSM 16795 | Bacteria; Firmicutes |
| mru1316 | TPR repeat-containing protein | Regulation | Protein interactions | 0.003 | 6E-17 | *Paramecium tetraurelia* strain d4-2 | Eukaryota; Intramacronucleata |
| mru0066 | serine phosphatase | Regulation | Protein interactions | 0.085 | 1E-47 | *Eggerthella lenta* DSM 2243 | Bacteria; Actinobacteria |
| mru1306 | serine/threonine protein kinase | Regulation | Protein interactions | 0.085 | 3E-38 | *Frankia* sp. EAN1pec | Bacteria; Actinobacteria |
| mru0513 | anti-sigma factor antagonist | Regulation | Protein interactions | 0.087 | 8E-10 | *Sphingomonas wittichii* RW1 | Bacteria; Proteobacteria |
| mru0516 | anti-sigma regulatory factor serine/threonine protein kinase | Regulation | Protein interactions | 0.087 | 2E-13 | *Bacteroides fragilis* YCH46 | Bacteria; Bacteroidetes |
| mru1168 | serine/threonine protein kinase with TPR repeats | Regulation | Protein interactions | 0.088 | 1E-33 | *Roseiflexus* sp. RS-1 | Bacteria; Chloroflexi |
| mru1288 | serine/threonine protein phosphatase | Regulation | Protein interactions | 0.088 | 3E-20 | *Stigmatella aurantiaca* DW4/3-1 | Bacteria; Proteobacteria |
| mru2116 | TPR repeat-containing protein | Regulation | Protein interactions | 0.096 | 8E-23 | *Cyanothece* sp. PCC 7424 | Bacteria; Cyanobacteria |
| mru2166 | TPR repeat-containing protein | Regulation | Protein interactions | 0.096 | 0.000002 | *Microcoleus chthonoplastes* PCC 7420 | Bacteria; Cyanobacteria |
| mru0514 | 4'-phosphopantetheinyl transferase family protein | Regulation | Protein interactions | 0.114 | 5E-25 | *Bacillus mycoides* Rock1-4 | Bacteria; Firmicutes |
| mru1295 | phosphotyrosine protein phosphatase | Regulation | Protein interactions | 0.12 | 4E-25 | *Halothermothrix orenii* H 168 | Bacteria; Firmicutes |
| mru0044 | serine phosphatase | Regulation | Protein interactions | 0.129 | 2E-15 | *Syntrophomonas wolfei* subsp. *wolfei* str. Goettingen | Bacteria; Firmicutes |
| mru0067 | anti-sigma factor antagonist | Regulation | Protein interactions | 0.129 | 3E-13 | Syntrophomonas wolfei subsp. *wolfei* str. Goettingen | Bacteria; Firmicutes |
| mru0039 | biotin-binding and phosphotyrosine protein phosphatase domain-containing protein | Regulation | Protein interactions | 0.134 | 1E-13 | *Clostridium thermocellum* ATCC 27405 | Bacteria; Firmicutes |
| mru0515 | serine phosphatase | Regulation | Protein interactions | 0.134 | 1E-55 | *Clostridium bolteae* ATCC BAA-613 | Bacteria; Firmicutes |
| mru0577 | transcriptional regulator TetR family | Regulation | Transcriptional regulators | 0.088 | 8E-22 | *Fusobacterium nucleatum* subsp. *polymorphum* ATCC 10953 | Bacteria; Fusobacteria |
| mru1338 | iron dependent repressor | Regulation | Transcriptional regulators | 0.088 | 6E-35 | *Pelobacter propionicus* DSM 2379 | Bacteria; Proteobacteria |
| mru1629 | transcriptional regulator MarR family | Regulation | Transcriptional regulators | 0.088 | 5E-18 | *Sebaldella termitidis* ATCC 33386 | Bacteria; Fusobacteria |
| mru1160 | transcriptional regulator | Regulation | Transcriptional regulators | 0.111 | 6E-12 | *Abiotrophia defectiva* ATCC 49176 | Bacteria; Firmicutes |
| mru0662 | transcriptional regulator LytR family | Regulation | Transcriptional regulators | 0.13 | 6E-21 | *Shuttleworthia satelles* DSM 14600 | Bacteria; Firmicutes |
| mru1739 | transcriptional regulator TetR family | Regulation | Transcriptional regulators | 0.13 | 6E-57 | *Oribacterium sinus* F0268 | Bacteria; Firmicutes |
| mru0351 | non-ribosomal peptide synthetase | Secondary metabolites | NRPS | 0.112 | 0 | *Brevibacillus parabrevis* | Bacteria; Firmicutes |
| mru0068 | non-ribosomal peptide synthetase | Secondary metabolites | NRPS | 0.129 | 0 | *Syntrophomonas wolfei* subsp. *wolfei* str. Goettingen | Bacteria; Firmicutes |
| mru1759 | amino acid carrier protein AGCS family | Transporters | Amino acids | 0.129 | 2E-164 | *Acidaminococcus* sp. D21 | Bacteria; Firmicutes |
| mru1945 | amino acid ABC transporter substrate-binding protein | Transporters | Amino acids | 0.13 | 3E-70 | *Dorea longicatena* DSM 13814 | Bacteria; Firmicutes |
| mru1340 | ferrous iron transport protein B FeoB1 | Transporters | Cations | 0.088 | 5E-158 | *Geobacter uraniireducens* Rf4 | Bacteria; Proteobacteria |
| mru0821 | transporter CDF family | Transporters | Cations | 0.093 | 4E-90 | *Slackia heliotrinireducens* DSM 20476 | Bacteria; Actinobacteria |
| mru0405 | transporter Na+/H+ antiporter family | Transporters | Cations | 0.134 | 3E-174 | *Clostridium* sp. 7_2_43FAA | Bacteria; Firmicutes |
| mru0358 | transporter small multidrug resistance (SMR) family | Transporters | Other | 0.087 | 2E-16 | *Yersinia bercovieri* ATCC 43970 | Bacteria; Proteobacteria |
| mru0369 | transporter small multidrug resistance (SMR) family | Transporters | Other | 0.087 | 2E-16 | *Yersinia bercovieri* ATCC 43970 | Bacteria; Proteobacteria |
| mru1201 | MFS transporter | Transporters | Other | 0.111 | 9E-49 | *Lactococcus lactis* subsp. *lactis* | Bacteria; Firmicutes |
| mru0559 | MFS transporter | Transporters | Other | 0.112 | 2E-29 | *Staphylococcus saprophyticus* subsp. *saprophyticus* ATCC 15305 | Bacteria; Firmicutes |
| mru0366 | ABC transporter ATP-binding/permease protein | Transporters | Other | 0.117 | 1E-21 | *Eubacterium biforme* DSM 3989 | Bacteria; Firmicutes |
| mru2176 | transporter permease family protein | Transporters | Other | 0.117 | 6E-22 | *Eubacterium biforme* DSM 3989 | Bacteria; Firmicutes |
| mru2177 | transporter permease family protein | Transporters | Other | 0.117 | 5E-83 | *Clostridium spiroforme* DSM 1552 | Bacteria; Firmicutes |
| mru1002 | MFS transporter | Transporters | Other | 0.129 | 4E-46 | *Thermosinus carboxydivorans* Nor1 | Bacteria; Firmicutes |
| mru1332 | Na+ dependent transporter SBF family | Transporters | Other | 0.129 | 4E-47 | *Acidaminococcus* sp. D21 | Bacteria; Firmicutes |
| mru0986 | transporter SDF family | Transporters | Other | 0.13 | 2E-121 | Eubacterium hallii DSM 3353 | Bacteria; Firmicutes |
| mru1628 | ABC transporter ATP-binding/permease protein | Transporters | Other | 0.13 | 6E-175 | *Anaerofustis stercorihominis* DSM 17244 | Bacteria; Firmicutes |
| mru0069 | MatE efflux family protein | Transporters | Other | 0.134 | 7E-28 | *Clostridium hiranonis* DSM 13275 | Bacteria; Firmicutes |
| mru0352 | MatE efflux family protein | Transporters | Other | 0.134 | 2E-22 | Clostridium sp. L2-50 | Bacteria; Firmicutes |
| mru0993 | transporter TDT family | Transporters | Other | 0.134 | 2E-26 | *Clostridium* sp. 7_2_43FAA | Bacteria; Firmicutes |
| mru1627 | ABC transporter ATP-binding/permease protein | Transporters | Other | 0.134 | 1E-112 | *Clostridium difficile* 630 | Bacteria; Firmicutes |
| mru0141 | transporter | Transporters | Other | 0.138 | 4E-58 | *Blautia hansenii* DSM 20583 | Bacteria; Firmicutes |
| mru1789 | transporter SDF family | Transporters | Other | 0.138 | 3E-107 | *Epulopiscium* sp. N.t. morphotype B | Bacteria; Firmicutes |
| mru0248 | acyltransferase | Unknown function | Enzyme | 0.084 | 2E-15 | *Sphingobacterium spiritivorum* ATCC 33300 | Bacteria; Bacteroidetes |
| mru1847 | NADP-dependent alcohol dehydrogenase Adh2 | Unknown function | Enzyme | 0.085 | 5E-65 | *Saccharopolyspora erythraea* NRRL 2338 | Bacteria; Actinobacteria |
| mru0052 | glycyl-radical enzyme activating protein | Unknown function | Enzyme | 0.087 | 6E-35 | *Bacteroides thetaiotaomicron* VPI-5482 | Bacteria; Bacteroidetes |
| mru0574 | acetyltransferase GNAT family | Unknown function | Enzyme | 0.087 | 5E-50 | *Oxalobacter formigenes* HOxBLS | Bacteria; Proteobacteria |
| mru0579 | oxidoreductase aldo/keto reductase family | Unknown function | Enzyme | 0.087 | 1E-131 | *Bacteroides capillosus* ATCC 29799 | Bacteria; Bacteroidetes |
| mru1757 | NADH-dependent flavin oxidoreductase | Unknown function | Enzyme | 0.087 | 1E-54 | *Campylobacter curvus* 525.92 | Bacteria; Proteobacteria |
| mru1758 | acetyltransferase | Unknown function | Enzyme | 0.087 | 3E-49 | *Bacteroides capillosus* ATCC 29799 | Bacteria; Bacteroidetes |
| mru1881 | acetyltransferase | Unknown function | Enzyme | 0.087 | 1E-51 | *Idiomarina loihiensis* L2TR | Bacteria; Proteobacteria |
| mru0612 | acetyltransferase GNAT family | Unknown function | Enzyme | 0.088 | 3E-40 | *Geobacter* sp. FRC-32 | Bacteria; Proteobacteria |
| mru1036 | hydrolase alpha/beta fold family | Unknown function | Enzyme | 0.088 | 3E-24 | *Nostoc punctiforme* PCC 73102 | Bacteria; Cyanobacteria |
| mru1502 | methyltransferase | Unknown function | Enzyme | 0.109 | 4E-57 | *Catenibacterium mitsuokai* DSM 15897 | Bacteria; Firmicutes |
| mru2170 | acetyltransferase | Unknown function | Enzyme | 0.111 | 1E-52 | *Pediococcus pentosaceus* ATCC 25745 | Bacteria; Firmicutes |
| mru0929 | manganese-dependent inorganic pyrophosphatase PpaC | Unknown function | Enzyme | 0.114 | 3E-85 | *Bacillus* sp. SG-1 | Bacteria; Firmicutes |
| mru1508 | hydrolase alpha/beta fold family | Unknown function | Enzyme | 0.114 | 2E-14 | *Bacillus clausii* KSM-K16 | Bacteria; Firmicutes |
| mru1534 | acyltransferase | Unknown function | Enzyme | 0.114 | 2E-16 | *Bacillus thuringiensis* *serovar konkukian* str. 97-27 | Bacteria; Firmicutes |
| mru0637 | SAM-dependent methyltransferase | Unknown function | Enzyme | 0.117 | 2E-34 | *Eubacterium biforme* DSM 3989 | Bacteria; Firmicutes |
| mru0512 | acyltransferase | Unknown function | Enzyme | 0.129 | 9E-25 | *Ruminococcus obeum* ATCC 29174 | Bacteria; Firmicutes |
| mru0610 | amidohydrolase | Unknown function | Enzyme | 0.13 | 1E-67 | *Eubacterium siraeum* DSM 15702 | Bacteria; Firmicutes |
| mru0779 | SAM dependent methyltransferase | Unknown function | Enzyme | 0.13 | 2E-42 | *Anaerostipes caccae* DSM 14662 | Bacteria; Firmicutes |
| mru0443 | short-chain dehydrogenase family protein | Unknown function | Enzyme | 0.132 | 7E-72 | *Alkaliphilus oremlandii* OhILAs | Bacteria; Firmicutes |
| mru0195 | SAM dependent methyltransferase | Unknown function | Enzyme | 0.134 | 3E-39 | *Clostridium sporogenes* ATCC 15579 | Bacteria; Firmicutes |
| mru0511 | hydrolase alpha/beta fold family | Unknown function | Enzyme | 0.134 | 5E-39 | *Clostridium kluyveri* DSM 555 | Bacteria; Firmicutes |
| mru0633 | acetyltransferase GNAT family | Unknown function | Enzyme | 0.134 | 8E-14 | *Clostridium acetobutylicum* ATCC 824 | Bacteria; Firmicutes |
| mru0646 | radical SAM domain-containing protein | Unknown function | Enzyme | 0.134 | 4E-102 | *Clostridium botulinum* A3 str. Loch Maree | Bacteria; Firmicutes |
| mru1120 | oxidoreductase aldo/keto reductase family | Unknown function | Enzyme | 0.134 | 3E-93 | *Clostridium kluyveri* DSM 555 | Bacteria; Firmicutes |
| mru1374 | acetyltransferase GNAT family | Unknown function | Enzyme | 0.134 | 7E-50 | *Clostridium scindens* ATCC 35704 | Bacteria; Firmicutes |
| mru1958 | short-chain dehydrogenase family protein | Unknown function | Enzyme | 0.134 | 2E-45 | *Clostridium cellulolyticum* H10 | Bacteria; Firmicutes |
| mru2164 | NADH:flavin oxidoreductase/NADH oxidase family protein | Unknown function | Enzyme | 0.134 | 2E-77 | *Clostridium cellulolyticum* H10 | Bacteria; Firmicutes |
| mru2198 | acetyltransferase GNAT family | Unknown function | Enzyme | 0.134 | 1E-19 | *Clostridium methylpentosum* DSM 5476 | Bacteria; Firmicutes |
| mru0491 | hydrolase alpha/beta fold family | Unknown function | Enzyme | 0.138 | 2E-55 | *Bryantella formatexigens* DSM 14469 | Bacteria; Firmicutes |
| mru0771 | hydrolase alpha/beta fold family | Unknown function | Enzyme | 0.138 | 2E-93 | *Blautia hydrogenotrophica* DSM 10507 | Bacteria; Firmicutes |
| mru1001 | SAM-dependent methyltransferase | Unknown function | Enzyme | 0.138 | 8E-60 | *Bryantella formatexigens* DSM 14469 | Bacteria; Firmicutes |
| mru0226 | hydrolase TatD family | Unknown function | Enzyme | 0.141 | 2E-38 | *Parvimonas micra* ATCC 33270 | Bacteria; Firmicutes |
| mru1304 | WD40 repeat-containing protein | Unknown function | General | 0.003 | 4E-26 | *Branchiostoma floridae* | Eukaryota; Metazoa |
| mru0547 | CAAX amino terminal protease family protein | Unknown function | General | 0.085 | 1E-17 | *Kocuria rhizophila* DC2201 | Bacteria; Actinobacteria |
| mru1593 | von Willebrand factor type A domain-containing protein | Unknown function | General | 0.087 | 2E-43 | *Campylobacter curvus* 525.92 | Bacteria; Proteobacteria |
| mru0095 | isoprenylcysteine carboxyl methyltransferase family protein | Unknown function | General | 0.111 | 9E-56 | *Streptococcus infantarius* subsp. *infantarius* ATCC BAA-102 | Bacteria; Firmicutes |
| mru0228 | pyridoxamine 5'-phosphate oxidase family protein | Unknown function | General | 0.129 | 2E-32 | *Mitsuokella multacida* DSM 20544 | Bacteria; Firmicutes |
| mru1738 | CAAX amino terminal protease family protein | Unknown function | General | 0.13 | 5E-83 | *Oribacterium sinus* F0268 | Bacteria; Firmicutes |
| mru1756 | thioesterase family protein | Unknown function | General | 0.13 | 4E-22 | *Eubacterium siraeum* DSM 15702 | Bacteria; Firmicutes |
| mru1860 | ATPase | Unknown function | General | 0.13 | 3E-92 | *Coprococcus eutactus* ATCC 27759 | Bacteria; Firmicutes |
| mru0517 | TfoX N-terminal domain-containing protein | Unknown function | General | 0.134 | 7E-29 | *Clostridium hiranonis* DSM 13275 | Bacteria; Firmicutes |
| mru1848 | TfoX C-terminal domain-containing protein | Unknown function | General | 0.134 | 3E-23 | *Clostridium kluyveri* DSM 555 | Bacteria; Firmicutes |
| mru0191 | pyridoxamine 5'-phosphate oxidase family protein | Unknown function | General | 0.138 | 6E-23 | *Bryantella formatexigens* DSM 14469 | Bacteria; Firmicutes |
| mru2084 | adenosylmethionine-8-amino-7-oxononanoate aminotransferase BioA | Vitamins and cofactors | Biotin | 0.088 | 3E-179 | *Brachyspira hyodysenteriae* WA1 | Bacteria; Spirochaetes |
| mru2087 | biotin synthase BioB1 | Vitamins and cofactors | Biotin | 0.088 | 3E-95 | *Brachyspira hyodysenteriae* WA1 | Bacteria; Spirochaetes |
| mru2041 | 6-carboxyhexanoate-CoA ligase BioW | Vitamins and cofactors | Biotin | 0.129 | 2E-47 | *Thermosinus carboxydivorans* Nor1 | Bacteria; Firmicutes |
| mru2042 | 8-amino-7-oxononanoate synthase BioF | Vitamins and cofactors | Biotin | 0.129 | 3E-73 | Acidaminococcus sp. D21 | Bacteria; Firmicutes |
| mru2086 | dethiobiotin synthetase BioD | Vitamins and cofactors | Biotin | 0.134 | 2E-59 | *Clostridium butyricum* 5521 | Bacteria; Firmicutes |
| mru0893 | cobyrinic acid a,c-diamide synthase CbiA3 | Vitamins and cofactors | Cobalamin | 0.087 | 2E-121 | *Bacteroides capillosus* ATCC 29799 | Bacteria; Bacteroidetes |
| mru0889 | cobalamin biosynthesis protein CbiG | Vitamins and cofactors | Cobalamin | 0.129 | 4E-67 | *Faecalibacterium prausnitzii* M21/2 | Bacteria; Firmicutes |
| mru0890 | precorrin-3B C17-methyltransferase CbiH1 | Vitamins and cofactors | Cobalamin | 0.129 | 1E-86 | *Ruminococcus gnavus* ATCC 29149 | Bacteria; Firmicutes |
| mru1543 | magnesium-protoporphyrin IX monomethyl ester anaerobic oxidative cyclase BchE | Vitamins and cofactors | Cobalamin | 0.129 | 0 | *Anaerotruncus colihominis* DSM 17241 | Bacteria; Firmicutes |
| mru0886 | precorrin-2 C20-methyltransferase CbiL | Vitamins and cofactors | Cobalamin | 0.134 | 1E-51 | *Clostridium phytofermentans* ISDg | Bacteria; Firmicutes |
| mru0887 | cobalamin biosynthesis protein CbiD | Vitamins and cofactors | Cobalamin | 0.134 | 1E-87 | *Clostridium phytofermentans* ISDg | Bacteria; Firmicutes |
| mru0888 | precorrin-4 C11-methyltransferase CbiF | Vitamins and cofactors | Cobalamin | 0.138 | 3E-102 | *Blautia hydrogenotrophica* DSM 10507 | Bacteria; Firmicutes |
| mru0891 | precorrin-6x reductase CbiJ | Vitamins and cofactors | Cobalamin | 0.138 | 2E-55 | Blautia hansenii DSM 20583 | Bacteria; Firmicutes |
| mru0892 | precorrin-6Y C5,15-methyltransferase (decarboxylating) CbiET | Vitamins and cofactors | Cobalamin | 0.138 | 3E-87 | *Blautia hansenii* DSM 20583 | Bacteria; Firmicutes |
| mru0894 | precorrin-8X methylmutase CbiC | Vitamins and cofactors | Cobalamin | 0.138 | 7E-82 | *Bryantella formatexigens* DSM 14469 | Bacteria; Firmicutes |
| mru0895 | cobalt chelatase CbiK | Vitamins and cofactors | Cobalamin | 0.138 | 2E-82 | *Bryantella formatexigens* DSM 14469 | Bacteria; Firmicutes |
| mru1377 | glutathione-disulfide reductase Gor2 | Vitamins and cofactors | Glutathione metabolism | 0.112 | 3E-94 | *Staphylococcus haemolyticus* JCSC1435 | Bacteria; Firmicutes |
| mru1935 | glutathione peroxidase GpxA | Vitamins and cofactors | Glutathione metabolism | 0.134 | 5E-54 | *Clostridium phytofermentans* ISDg | Bacteria; Firmicutes |
| mru1430 | NAD+ synthetase NadE | Vitamins and cofactors | Nicotinate | 0.129 | 1E-66 | *Veillonella parvula* DSM 2008 | Bacteria; Firmicutes |
| mru0189 | ATP-NAD kinase | Vitamins and cofactors | Nicotinate | 0.13 | 1E-37 | *Coprococcus comes* ATCC 27758 | Bacteria; Firmicutes |
| mru0089 | 3,4-dihydroxy-2-butanone 4-phosphate synthase RibB | Vitamins and cofactors | Riboflavin | 0.088 | 4E-84 | *Desulfovibrio salexigens* DSM 2638 | Bacteria; Proteobacteria |
| mru0198 | hydroxymethylpyrimidine transporter CytX | Vitamins and cofactors | Thiamine | 0.13 | 3E-61 | *Dorea formicigenerans* ATCC 27755 | Bacteria; Firmicutes |
| mru0199 | phosphomethylpyrimidine kinase ThiD1 | Vitamins and cofactors | Thiamine | 0.13 | 4E-59 | *Eubacterium ventriosum* ATCC 27560 | Bacteria; Firmicutes |
| mru0227 | ThiF family protein | Vitamins and cofactors | Thiamine | 0.134 | 5E-68 | *Clostridium thermocellum* ATCC 27405 | Bacteria; Firmicutes |
