# Supplementary material for: The Genome Sequence of the Rumen Methanogen Methanobrevibacter ruminantium Reveals New Possibilities for Controlling Ruminant Methane Emissions
Source: PLoS One. 2010 Jan 28;5(1):e8926. doi: 10.1371/journal.pone.0008926 (PMC2812497; doi:10.1371/journal.pone.0008926)
Supplement: Table S3 — Selection of upregulated genes of the M1 genome when grown in co-culture with Butyrivibrio proteoclasticus B316. (0.04 MB DOC) [file pone.0008926.s003.doc]

| **Table S3.** Selection of upregulated genes of the M1 genome when grown in co-culture with *Butyrivibrio proteoclasticus* B316. | | |
| --- | --- | --- |
| **Locus tag** | **Annotation** | **Fold difference** |
|  | **Energy metabolism - formate metabolism** |  |
| mru0333 | formate dehydrogenase alpha subunit FdhA | 3.46 |
| mru0334 | formate dehydrogenase beta subunit FdhB | 2.34 |
|  | **Energy metabolism - methanogenesis** |  |
| mru1928 | methyl-coenzyme M reductase beta subunit McrB | 2.26 |
| mru1926 | methyl-coenzyme M reductase C subunit McrC | 2.39 |
| mru1927 | methyl-coenzyme M reductase D subunit McrD | 2.23 |
| mru1925 | methyl-coenzyme M reductase gamma subunit McrG | 3.41 |
| mru1919 | tetrahydromethanopterin S-methyltransferase subunit A MtrA | 2.02 |
| mru1920 | tetrahydromethanopterin S-methyltransferase subunit B MtrB | 2.23 |
| mru1921 | tetrahydromethanopterin S-methyltransferase subunit C MtrC | 3.23 |
| mru1916 | tetrahydromethanopterin S-methyltransferase subunit H MtrH | 2.14 |
| mru1907 | methyl viologen-reducing hydrogenase gamma subunit MvhG | 2.25 |
| mru0344 | tungsten formylmethanofuran dehydrogenase subunit A FwdA | 2.12 |
|  | **Cell envelope - Cell surface** |  |
| mru2090 | adhesin-like protein | 2.31 |
| mru2134 | adhesin-like protein | 2.28 |
| mru1222 | adhesin-like protein | 4.08 |
| mru0076 | adhesin-like protein | 2.01 |
| mru1499 | adhesin-like protein with transglutaminase domain | 2.14 |
| mru0828 | adhesin-like protein with transglutaminase domain | 2.50 |
