# Supplementary material for: The Genome Sequence of the Rumen Methanogen Methanobrevibacter ruminantium Reveals New Possibilities for Controlling Ruminant Methane Emissions
Source: PLoS One. 2010 Jan 28;5(1):e8926. doi: 10.1371/journal.pone.0008926 (PMC2812497; doi:10.1371/journal.pone.0008926)
Supplement: Table S4 — Predicted cell surface associated adhesin-like proteins in M1. (1.00 MB RTF) [file pone.0008926.s004.rtf]

	Pfam1 & TigrFam domains2	
	TM C-terminal	M1 C-terminal domain (M1-C)	Transglutaminase domain	Cysteine protease	Chlamydial POMP repeat	DUF11	B_ant_repeat	Haemagluttinin	DUF1565	Big_1	Big_2	Pseudomurein-binding repeat	String of G's	YD repeat	Cna protein B-type domain	LPxTG	Cell wall binding repeat	Alpha-2-macroglobulin	Morn repeat variant	RNA polymerase Rpb1 C-terminal repeat	GLUG motif	Staphylocoagulase repeat	
Locus tag	Annotation	Size (bp)	Signal3	TM4		
Group 1: Transmembrane C-terminal Group	
mru0019	adhesin-like protein	1220	SP	2	 																						
mru0327	adhesin-like protein	2090	SP	2	 																						
mru0687	adhesin-like protein	2963	SP	2	 				 																		
mru1210	adhesin-like protein	7250	SP	2	 				 	 	 																
mru1222	adhesin-like protein	4055	SP	2	 																						
mru1506	adhesin-like protein	857	SP	2	 					 																	
mru2053	adhesin-like protein	3494	SP	2	 																						
mru2134	adhesin-like protein	17957	SP	2	 				 	 	 							 	 								
mru2147	adhesin-like protein	16955	SP	4	 				 	 	 									 	 						
mru2178	adhesin-like protein	9239	SP	2	 				 	 	 																
mru0031	adhesin-like protein	4415	CW	1	 					 	 																
mru0704	adhesin-like protein	2858	SP	1	 																						
mru0963	adhesin-like protein	8159	CW	0	 																						
mru0976/0977	adhesin-like protein 	4775	SP	2	 				 								 										
Group2: M1-Big_1 like C-terminal Group	
mru0020	adhesin-like protein with cysteine protease domain	6014	SP	1		 		 	 																		
mru0064	adhesin-like protein	3536	SP	1		 			 																		
mru0072	adhesin-like protein	2918	SP	1		 																					
mru0076	adhesin-like protein	6605	SP	1		 																					
mru0077	adhesin-like protein	9161	SP	1		 																					
mru0079	adhesin-like protein	3560	SP	1		 																					
mru0083	adhesin-like protein	839	SP	1		 																					
mru0084	adhesin-like protein	14477	SP	1		 			 			 															
mru0085	adhesin-like protein	8030	SP	1		 			 																		
mru0086	adhesin-like protein	10175	SP	1		 			 				 														
mru0143	adhesin-like protein with cysteine protease domain	3284	SP	0		 		 	 																		
mru0160	adhesin-like protein	3176	SP	1		 																					
mru0222	adhesin-like protein with cysteine protease domain	3302	SP	1		 		 	 																		
mru0327	adhesin-like protein	2060	SP	1		 																					
mru0338	adhesin-like protein	6929	SP	1		 			 																		
mru0417/0418	adhesin-like protein	1391	SP	1		 			 					 													
mru0419	adhesin-like protein	4175	SP	1		 			 						 												
mru0727	adhesin-like protein with cysteine protease domain	3788	SP	0		 		 	 																		
mru0772	adhesin-like protein with cysteine protease domain	3281	SP	1		 		 																			
mru0839	adhesin-like protein with cysteine protease domain	8639	SP	1		 		 	 																		
mru0842	adhesin-like protein with cysteine protease domain	3977	SP	1		 		 	 																		
mru0978	adhesin-like protein	6606	SP	0		 			 																		
mru0979	adhesin-like protein	8753	SP	1		 			 					 								 					
mru1076	adhesin-like protein	2681	SP	1		 			 					 													
mru1077	adhesin-like protein	2273	SP	1		 			 																		
mru1246	adhesin-like protein	4619	SP	1		 			 	 	 																
mru1247	adhesin-like protein	5060	SP	1		 			 	 	 				 												
mru1465	adhesin-like protein	2882	SP	1		 			 																		
mru1513	adhesin-like protein	1853	SP	1		 			 																		
mru1650	adhesin-like protein	9161	SP	1		 			 					 					 								
mru1726	adhesin-like protein	6767	SP	1		 			 						 												
mru1971	adhesin-like protein	1937	SP	1		 																					
mru1996	adhesin-like protein	4496	SP	1		 																					
mru2043	adhesin-like protein	9530	SP	1		 			 														 	 			
mru2048	adhesin-like protein	5417	SP	1		 																					
mru2049	adhesin-like protein	10355	SP	1		 			 																		
mru2052	adhesin-like protein	4112	SP	1		 																					
mru2054	adhesin-like protein	5054	SP	1		 																					
mru2055	adhesin-like protein	8906	SP	1		 			 	 	 																
mru2059	adhesin-like protein	4415	SP	0		 																					
mru2090	adhesin-like protein	15200	SP	1		 			 			 													 		
mru0004	adhesin-like protein	2237	SP	1		 																					
mru0331	adhesin-like protein	1622	SP	1		 								 													
mru0843	adhesin-like protein with cysteine protease domain	6197	SP	1		 		 	 																		
Group 3 : Other	
mru0015	adhesin-like protein with cysteine protease domain	3845	SP	1				 	 																		
mru0090	adhesin-like protein	2063	SP	1					 																		
mru0255	adhesin-like protein	4187	SP	1																							
mru0450	adhesin-like protein	803	SP	1																							
mru0723	adhesin-like protein	7727	SP	1										 												 	
mru0962	adhesin-like protein	14789	SP	1																							
mru0970	adhesin-like protein	2483	SP	1																							
mru1263	adhesin-like protein	2585	SP	1					 																		
mru1358	adhesin-like protein	2243	SP	1																							
mru1386	adhesin-like protein	1841	SP	1																							
mru1387	adhesin-like protein with cysteine protease domain	2957	SP	1				 																			
mru1424	adhesin-like protein	1445	SP	1																							
mru1500	adhesin-like protein	3896	SP	1					 																		
Group 4:  Pseudomurein-binding Group 	
mru0493	adhesin-like protein	2447	SP	1												 											
mru0824	adhesin-like protein with transglutaminase domain	2027	SP	1			 									 											
mru1499	adhesin-like protein with transglutaminase domain	3032	SP	0			 							 		 											
mru1604	adhesin-like protein with transglutaminase domain	2996	SP	0			 							 		 											

1Pfam: Transglutaminase (PF01841), Papain family cysteine protease (PF00112), Domain of unknown function DUF11 (PF01345), Haemagluttinin repeat (PF05594), Protein of unknown function DUF1565 (PF07602), Group 1 Bacterial Ig-like domain Big_1 (PF02369), Group 2 Bacterial Ig-like domain Big_2 (PF02368), Pseudomurein-binding repeat (PF09373), CNA protein B-type domain (PF05738), Cell wall binding repeat (PF01473), Alpha-2-macroglobulin (PF01835), Morn repeat variant (PF07661), RNA polymerase Rpb1 C-terminal repeat (PF05001), GLUG motif (PF07581), Staphylocoagulase repeat (PF04022).
2TigrFam: Chlamydial POMP repeat (TIGR01376), conserved repeat domain B_ant_repeat (TIGR01451), YD repeat (TIGR01643).
3Signal: SP, signal peptide as determined by SignalP3.0; CW, cell wall as determined by PSORT
4TM: TM, transmembrane domain predictions completed using www.cbs.dtu.dk/services/TMHMM/	
