# Supplementary material for: The Genome Sequence of the Rumen Methanogen Methanobrevibacter ruminantium Reveals New Possibilities for Controlling Ruminant Methane Emissions
Source: PLoS One. 2010 Jan 28;5(1):e8926. doi: 10.1371/journal.pone.0008926 (PMC2812497; doi:10.1371/journal.pone.0008926)
Supplement: Table S5 — Potential chemogenomic gene targets of the M1 genome based on in-depth literature and metabolic analyses. (0.17 MB DOC) [file pone.0008926.s005.doc]

| **Table S5**. Potential chemogenomic gene targets of the M1 genome based on in-depth literature and metabolic analyses. | | |
| --- | --- | --- |
| **Locus** | **Annotation** | **Reference** |
|  | | |
| **AMINO ACID METABOLISM** | | |
|  | | |
| mru0997 | phospho-2-dehydro-3-deoxyheptonate aldolase/ fructose- bisphosphate aldolase | [S8,9] |
| mru0998 | AroB | [S9-11] |
| mru1577 | AroA | [S9,10,12] |
| mru1676 | AroK | [S13] |
| mru0350  mru2078 | GlnA1  GlnA2 | [S14] |
| mru0122 | GlyA | [S15-17] |
| mru2139 | HisB | [S18] |
| mru0152 | LysA | [S19-21] |
| mru0153 | DapF | [S21,22] |
| mru1743 | PdaD | [S23, 24] |
| mru0208 | TrpE | [S25] |
| mru0410  mru2112  mru2111 | IlvB1  IlvB2  IlvN | [S26, 27] |
| mru1414 | CimA | [S28, 29] |
|  | | |
| **CELL CYCLE** | | |
|  | | |
| mru0481 | FtsZ | [S30, 31] |
| mru0240  mru2212 | PolD2  PolD1 | [S32] |
| mru1864  mru1865 | DNA topoisomerase VI subunit A  DNA topoisomerase VI subunit B | [S33, 34] |
|  | | |
| **CELL ENVELOPE** | | |
|  | | |
| mru0824  mru0828  mru1497  mru1499  mru1604 | adhesin-like protein with transglutaminase domain  adhesin-like protein with transglutaminase domain  adhesin-like protein with transglutaminase domain  adhesin-like protein with transglutaminase domain  adhesin-like protein with transglutaminase domain | [S35- 44] |
| mru1836 | cell shape determining protein MreB/Mrl family | [S45-47] |
| mru1047 | poly-gamma-glutamate biosynthesis protein | [S48,49] |
| mru2175 | cell wall biosynthesis glycosyl transferase |  |
| mru0707  mru1042  mru1118  mru1745  mru2091  mru2092 | cell wall biosynthesis protein Mur ligase family  cell wall biosynthesis protein Mur ligase family  cell wall biosynthesis protein Mur ligase family  cell wall biosynthesis protein Mur ligase family  cell wall biosynthesis protein Mur ligase family  cell wall biosynthesis protein Mur ligase family | [S50-54] |
| mru0964  mru1041 | cell wall biosynthesis protein phospho-N-acetylmuramoyl-pentapeptide-transferase family  cell wall biosynthesis protein phospho-N-acetylmuramoyl-pentapeptide-transferase family | [S55-57] |
| mru2126 | cell wall biosynthesis protein UDP-glycosyltransferase family |  |
| mru1293  mru1536 | GlmS1  GlmS2 | [S52, 58] |
| mru1388  mru1413 | NAD dependent epimerase/dehydratase  NAD dependent epimerase/dehydratase | [S59] |
| mru0458  mru0449 | GlmM1  GlmM2 | [S52, 58] |
| mru1733 | phosphosugar-binding protein |  |
| mru2136 | polysaccharide biosynthesis protein |  |
| mru1470 | GalE | [S59] |
| mru0456 | GlmU | [S52, 58] |
| mru1005 | UppS | [S60, 61] |
| mru2108 | UppP | [S52,62-64] |
| mru1524 | polysaccharide biosynthesis protein | [S65] |
|  | | |
| **CENTRAL CARBON METABOLISM** | | |
|  | | |
| mru1434 | AcsA | [S66] |
| mru1570 | Acs | [S66-68] |
| mru0550mru0551  mru0549  mru0548  mru0552  mru0553 | PorA  PorB  PorD  PorD  PorE  PorF | [S69-74] |
| mru0957 | RpiA | [S75-77] |
| mru1634 | Prs | [S78] |
| mru0250  mru1310 | Phi1  Phi2 | [S75,77, 79-81] |
| mru2131 | Fae/Hps | [S75-77,81, 82] |
| mru1255 | Mdh | [S83-86] |
| mru0847  mru1888 | PycA  PycB | [S87-90] |
| mru0088  mru0655 | SdhA  SdhB | [S91, 92] |
|  | | |
| **ENERGY METABOLISM** | | |
|  | | |
| mru0701  mru0702  mru0699  mru0703  mru0698  mru0700  mru0695  mru0696  mru0697 | AhaA  AhaB  AhaC  AhaD  AhaE  AhaF  AhaH  AhaI  AhaK | [S93-105] |
| mru2064  mru2061 mru2081 mru2063 mru2062 | FrhA  FrhB1  FrhB2  FrhD  FrhG | [S106, 107] |
| mru1412  mru1411  mru1410  mru1409  mru1408  mru1407  mru1406  mru1405  mru1404  mru1403  mru1402  mru1401  mru1400  mru1399  mru1398  mru1397  mru1396  mru1394 | EhaA  EhaB  EhaC  EhaD  EhaE  EhaF  EhaG  EhaH  EhaI  EhaJ  EhaK  EhaL  EhaM  EhaN  EhaO  EhaP  EhaQ  EhaR | [S108, 109] |
| mru2014  mru2013  mru2012  mru2011  mru2010  mru2009  mru2008  mru2007  mru2006  mru2005  mru2004  mru2003  mru2002  mru2001  mru2000  mru1999  mru1998 | EhbA  EhbB  EhbC  EhbD  EhbE  EhbF  EhbG  EhbH  EhbI  EhbJ  EhbK  EhbL  EhbM  EhbN  EhbO  EhbP  EhbQ | [S108, 110] |
| mru1906  mru1905  mru1908  mru2076  mru1907 | MvhA  MvhB  MvhD1  MvhD2  MvhG | [S111-113] |
| mru0569 | Mer | [S106, 114-116] |
| mru0117  mru0817  mru1212  mru0816 | HdrA  HdrB  HdrB2  HdrC | [S106, 115,  ^117-122]^ |
| mru0526 | Hmd | [S106, 123-127] |
| mru2142 | Mtd | [S126, 128-131] |
| mru1393  mru2022 | Ftr1  Ftr2 | [S106, 115-116, 132-133] |
| mru1619 | Mch | [S106, 114, 115, 129,  ^134-136]^ |
| mru1924  mru1928  mru1926  mru1927  mru1925  mru1262  mru1850 | McrA  McrB  McrC  McrD  McrG  AtwA1  AtwA2 | [S106, 114, 115, 137-151] |
| mru1919  mru0441  mru1920  mru1921  mru1922  mru1923  mru1918  mru1917  mru1916 | MtrA1  MtrA2  MtrB  MtrC  MtrD  MtrE  MtrF  MtrG  MtrH | [S106, 115,  ^121, 152-155]^ |
| mru0344  mru0343  mru0345  mru0342  mru0254  mru0340  mru0341  mru0339 | FwdA  FwdB  FwdC  FwdD  FwdE  FwdF  FwdG  FwdH | S121, 156-162] |
|  | | |
| **LIPID METABOLISM** | | |
|  | | |
| mru1031  mru1630 | FabG1  FabG2 | [S163-167] |
| mru0955 | EgsA | [S77, 168, 169] |
| mru1092 | HmgA | [S170-175] |
| mru1640 | hydroxymethylglutaryl-CoA synthase | [S169, 172, 173, 176, 177] |
| mru0922 | Fni | [S77, 176, 178-180] |
| mru0921 | isopentenyl diphosphate kinase | [S77, 181] |
| mru0920 | Mvk | [S170-175] |
| mru0919 | phosphomevalonate decarboxylase | [S77] |
| mru1102 | digeranylgeranylglyceryl phosphate synthase | [S182] |
| mru0924 | IdsA | [S177] |
|  | | |
| **MOBILE ELEMENTS** | | |
|  | | |
| mru0317 | phage-related protein | [S35] |
| mru0320 | endoisopeptidase PeiR | [S171] |
|  | | |
| **PROTEIN FATE** | | |
|  | | |
| mru2021 | transglutaminase domain-containing protein | [S35] |
| mru0391 | oligosaccharyl transferase |  |
| mru1832 | sortase family protein | [S183, 184] |
|  | | |
| **PROTEIN SYNTHESIS** | | |
| mru2169  mru2029  mru1142 | GatA  GatB  GatC | [S185-189] |
| mru1571 | CysS | [S185, 186,  ^190]^ |
| mru1427  mru1426 | GatD  GatE | [S185-187, 191-193] |
| mru0126 | IleS | [S186, 194-196] |
| mru0242 | LysS | [S185-187] |
| mru0954 | ProS | [S197, 198] |
| mru1947 | SerS | [S186, 199, 200] |
|  | | |
| **PURINES AND PYRIMIDINES** | | |
|  | | |
| mru1839 | PurO | [S77, 201, 202] |
| mru0595 | PurP | [S77, 203] |
| mru1055 | PyrF | [S204, 205] |
|  | | |
| **TRANSCRIPTION** | | |
|  | | |
| mru1482  mru1481  mru1815  mru1814  mru1816  mru1817  mru0908  mru0161  mru1818  mru0913  mru0169  mru0912  mru1350 | RpoE1  RpoE2  RpoA1  RpoA2  RpoB1  RpoB2  RpoD  RpoF  RpoH  RpoK  RpoL  RpoN  RpoP | [S206, 207] |
|  | | |
| **TRANSPORTERS** | | |
|  | | |
| mru0405 | transporter Na+/H+ antiporter family | [S208] |
|  |  |  |
| **UNKNOWN FUNCTION** | | |
|  | | |
| mru0668  mru1929  mru0097  mru0181  mru1915  mru1771mru1778  mru1774  mru1931  mru0436  mru1695 | methanogenesis marker protein 1  methanogenesis marker protein 10  methanogenesis marker protein 11  methanogenesis marker protein 13  methanogenesis marker protein 14  methanogenesis marker protein 15  methanogenesis marker protein 2  methanogenesis marker protein 3  methanogenesis marker protein 7  methanogenesis marker protein 8  H4MPT-linked C1 transfer pathway protein | [S209] |
|  | | |
| VITAMINS AND COFACTORS | | |
| mru1560 | HemB | [S153, 210-212] |
| mru1541 | CobA | [S153, 210-212] |
| mru1853 | HemA | [S153, 210-215] |
| mru1544 | HemD | [S153, 210-212, 216] |
| mru0999 | HemL | [S210-214] |
| mru1746 | HemC | [S153, 211-212] |
| mru0384 | AksD | [S217-219] |
| mru1689 | AksE | [S217-219] |
| mru0385 | AksA | [S217, 219, 220] |
| mru1033 | AksF | [S217, 219,  ^221]^ |
| mru1283 | ArfB | [S222] |
| mru0953 | CofC | [S217, 223] |
| mru1253  mru1787 | FtsA1  FtsA2 | [S217, 224-226] |
| mru0479 | F_420_-0:gamma-glutamyl ligase | [S217] |
| mru1842 | CofE | [S217, 227-229] |
| mru1974  mru1266 | CofG  CofH | [S230-232] |
| mru2213 | FucA | [S233-236] |
| mru0672 | CofA | [S235] |
| mru1844 | CofD | [S237, 238] |
| mru1949 | ComB | [S239, 240] |
| mru1980 | ComC | [S82, 217, 241, 242] |
| mru1896 | MfnA | [S217, 243] |
| mru1690 | MptG | [S244-249] |
| mru1962 | MptA | [S250, 251] |
| mru1559 | CitG | [S252-254] |
| mru1845 | ArfA | [S255, 256] |
| mru1215 | RibC | [S257-262] |
| mru0098 | RibH | [S257-259] |
| mru1007 | RibD | [S263] |
| mru2174 | RibK | [S264, 265] |
|  | | |
