# Supplementary material for: The Genome Sequence of the Rumen Methanogen Methanobrevibacter ruminantium Reveals New Possibilities for Controlling Ruminant Methane Emissions
Source: PLoS One. 2010 Jan 28;5(1):e8926. doi: 10.1371/journal.pone.0008926 (PMC2812497; doi:10.1371/journal.pone.0008926)
Supplement: Table S6 — Summary of the functional genome distribution analysis. (0.06 MB DOC) [file pone.0008926.s006.doc]

| **Table S6.** Summary of the Functional Genome Distribution Analysis. Identification of conserved M1 specific (left table panel) and sub-cluster 1.1 specific gene sets (right table panel). Conserved and cluster specific gene sets are referenced to M1. Cluster designations refer to those shown in Figure S5. Cut-offs indicate the e-value thresholds for conserved (low cut-off) and unique (high cut-off) gene selection criteria. Mismatch tolerance indicates the number of organisms specific ORFs allowed outside the respective thresholds. | | **Conserved genes, *M. ruminantium*-centric** | | | |  | **Cluster specific genes, *M. ruminantium*-centric^(A)^** | | | |
| --- | --- | --- | --- | --- | --- | --- | --- | --- | --- | --- |
|  |  | ***M. ruminantium* --- Cluster 1.1^(B)^** | **Cluster 1.1  --- Cluster 1 + 2** | ***M. ruminantium* --- Cluster 1 + 2** | **PMP^(C)^ --- Cluster 1 + 2** |  | ***M. ruminantium* --- Cluster 1.1** | **Cluster 1.1  --- Cluster 1 + 2** | ***M. ruminantium* --- Cluster 1 + 2** | **PMP^(C)^ --- Cluster 1 + 2** |
| **Thresholds:** | **Low cutoff High cutoff Mismatch tolerance** | **1e-100  0** | **1e-100  0** | **1e-100  0** | **1e-100  0** |  | **1e-100 1e-10 0** | **1e-60 1e-10 2** | **1e-60 1e-10 2** | **1e-60 1e-10 2** |
| **Classification** | **Sub classification** |  |  |  |  |  |  |  |  |  |
| Amino acid biosynthesis |  | 41 | 10 | 10 | 10 |  |  |  |  |  |
| Amino acid degradation |  | 2 | 1 | 1 | 2 |  |  |  |  |  |
| Cell cycle |  | 18 | 3 | 3 | 3 |  | 2 | 1 | 2 | 1 |
| Cell envelope | Pseudomurein biosynthesis Exopolysaccharides Cell surface proteins other | 10 4 | 1 | 1 | 1 |  | 4 7 | 5 5 9 3 | 5 10 | 5 4 2 1 |
| Cellular processes | Oxidative stress response Stress response | 2 |  |  |  |  | 1 | 1 | 1 | 1 |
| Central carbon metabolism |  | 24 | 2 | 2 | 2 |  | 1 | 4 | 1 | 3 |
| Energy metabolism | Methanogenesis Hydrogen metabolism Electron transport Formate | 22 7 4 | 6 1 2 | 6 1 2 | 5  1  2 |  | 3 | 2 2 | 1 3 | 2 |
| Hypothetical | Conserved | 15 | 1 | 1 | 1 |  | 306 | 22 | 330 | 18 |
| Lipid metabolism |  | 6 | 2 | 2 | 2 |  | 1 | 1 | 1 | 1 |
| Mobile elements | Transposase Prophage CRISPR-associated genes |  |  |  |  |  | 3 52 1 | 1 | 3 55 7 | 1 |
| Nitrogen metabolism |  | 3 |  |  |  |  |  |  | 1 |  |
| Nucleic acid metabolism |  | 16 | 4 | 4 | 4 |  | 9 | 2 | 8 |  |
| Protein fate |  | 15 | 3 | 3 | 4 |  | 1 | 4 | 2 |  |
| Protein synthesis |  | 46 | 23 | 23 | 24 |  | 3 | 1 | 6 | 1 |
| Purines & pyrimidines |  | 17 | 4 | 4 | 4 |  |  |  |  |  |
| Regulation |  | 1 |  |  |  |  | 22 |  | 23 |  |
| Transcription |  | 5 | 3 | 3 | 3 |  |  |  |  |  |
| Transporters |  | 6 |  |  |  |  | 10 | 3 | 4 | 1 |
| Unknown function | Enzyme General | 9 8 | 1 | 1 | 1 1 |  | 20 15 | 3 2 | 13 11 | 2 2 |
| Vitamins and cofactors | Cobalamin Coenzyme F_420_ Methanopterin Ubiquinone Thiamine Other Metal-binding pterin Methanofuran Coenzyme B Coenzyme M Biotin Glutathione metabolism Nicotinate | 6 3 1 2 3 3 1 1 2 1 | 1    1 | 1    1 | 1    1 |  | 1    2      3 1 | 1      1 | 2    2 1      4 | 1 |

(A) All cluster-specific analyses refer to *M. ruminantium* or the *M. ruminantium* functional cluster, (B) Cluster designations follow Figure S5**,** (C) PMP: Pseudomurein Producers
