# Supplementary material for: The Genome Sequence of the Rumen Methanogen Methanobrevibacter ruminantium Reveals New Possibilities for Controlling Ruminant Methane Emissions
Source: PLoS One. 2010 Jan 28;5(1):e8926. doi: 10.1371/journal.pone.0008926 (PMC2812497; doi:10.1371/journal.pone.0008926)
Supplement: Table S7 — Genome sequences used in this study. (0.04 MB DOC) [file pone.0008926.s007.doc]

| **Table S7. Genome sequences used in this study** | |
| --- | --- |
| **Organism** | **Genome accession number** |
| *Methanobrevibacter ruminantium* M1 | CP001719 |
| *Methanobrevibacter smithii* PS | CP000678 |
| *Methanobrevibacter smithii* F1 | ABYV00000000 |
| *Methanobrevibacter smithii* ALI | ABYW00000000 |
| *Methanocaldococcus jannaschii* JAL-1 | L77117 |
| *Methanococcoides burtonii* ACE-M | NC_007955 |
| *Methanococcus aeolicus Nankai*-3 | CP000743 |
| *Methanococcus maripaludis* C5 | CP000609 |
| *Methanococcus maripaludis* C6 | CP000867 |
| *Methanococcus maripaludis* C7 | CP000745 |
| *Methanococcus maripaludis* S2 | BX950229 |
| *Methanococcus vannielii* SB | CP000742 |
| *Methanococcus voltae* A3 | ABHB00000000 |
| *Methanocorpusculum labreanum* Z | CP000559 |
| *Methanoculleus marisnigri* JR1 | CP000562 |
| *Methanopyrus kandleri* AV19 | AE009439 |
| *Candidatus* Methanoregula boonei 6A8 | CP000780 |
| *Methanosaeta thermophila* PT | CP000477 |
| *Methanosarcina acetivorans* C2A | AE010299 |
| *Methanosarcina barkeri* Fusaro | CP000099 |
| *Methanosarcina mazei* strain Goe1 | AE008384 |
| *Methanosphaera stadtmanae* MCB-3 | CP000102 |
| *Candidatus Methanosphaerula palustris* E1-9c | CP001338 |
| *Methanospirillum hungatei* JF-1 | CP000254 |
| *Methanothermobacter thermautotrophicus* ΔH | NC_000916 |
| Uncultured methanogenic archaeon RC-I | AM114193 |
| *Syntrophomonas wolfei* subsp. *wolfei* Goettingen | CP000448 |
