# Supplementary figures and images for: The Genome Sequence of the Rumen Methanogen Methanobrevibacter ruminantium Reveals New Possibilities for Controlling Ruminant Methane Emissions
Source: PLoS One. 2010 Jan 28;5(1):e8926. doi: 10.1371/journal.pone.0008926 (PMC2812497; doi:10.1371/journal.pone.0008926)

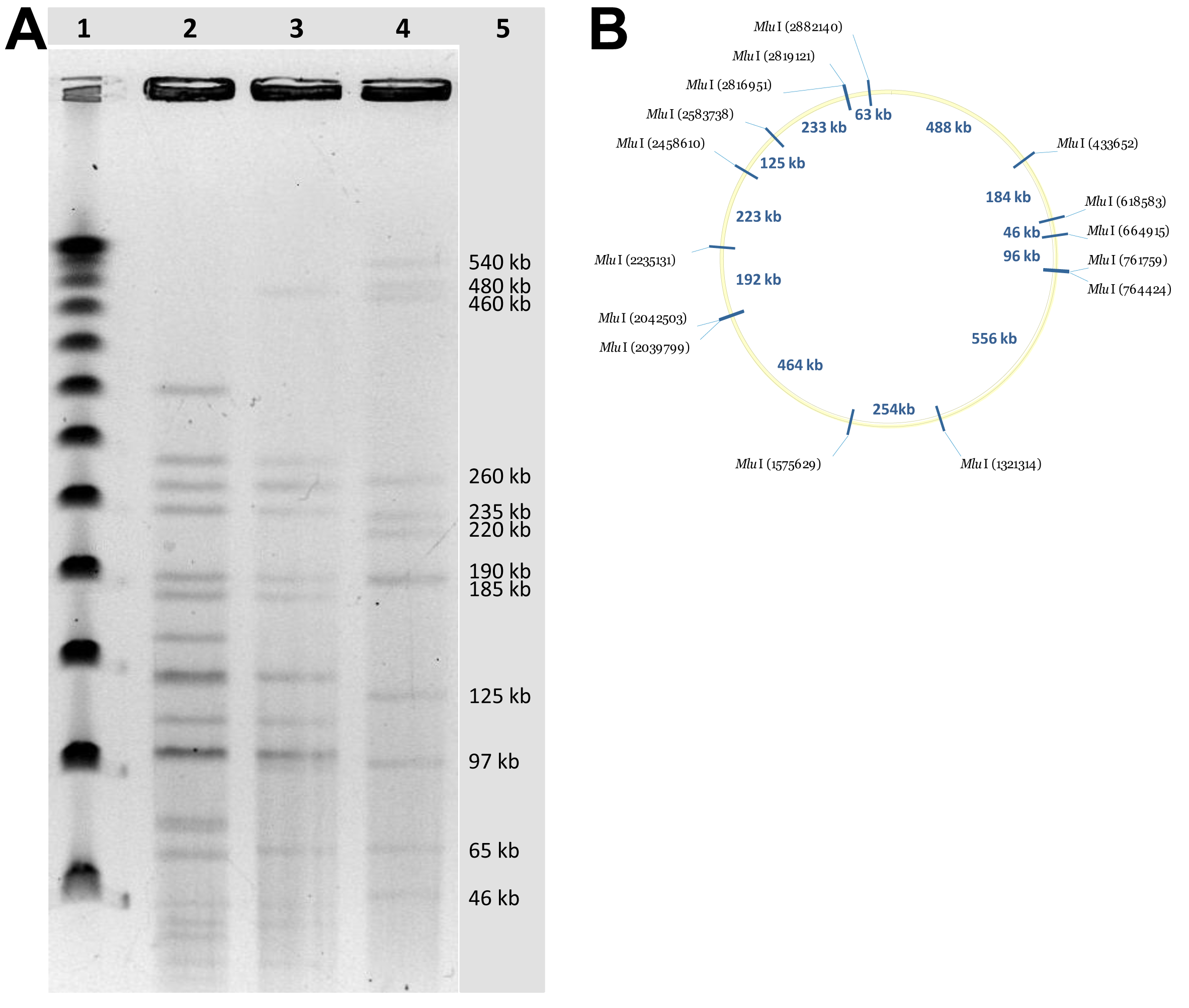

Supplement: Figure S1 — (A) PFGE of genomic DNA from M1. Lane 1, λ ladder (New England Biolabs); Lane 2, ApaI/BssHII double digest; Lane 3, ApaI digest; Lane 4, MluI digest; Lane 5, Sizes of MluI fragments. The bands in the λ ladder are multiples of 48.5 kb. (B) In silico restriction map of the M1 chromosome showing the position and fragment size of the Mlu1 digest. (0.98 MB TIF) [file pone.0008926.s008.tif]

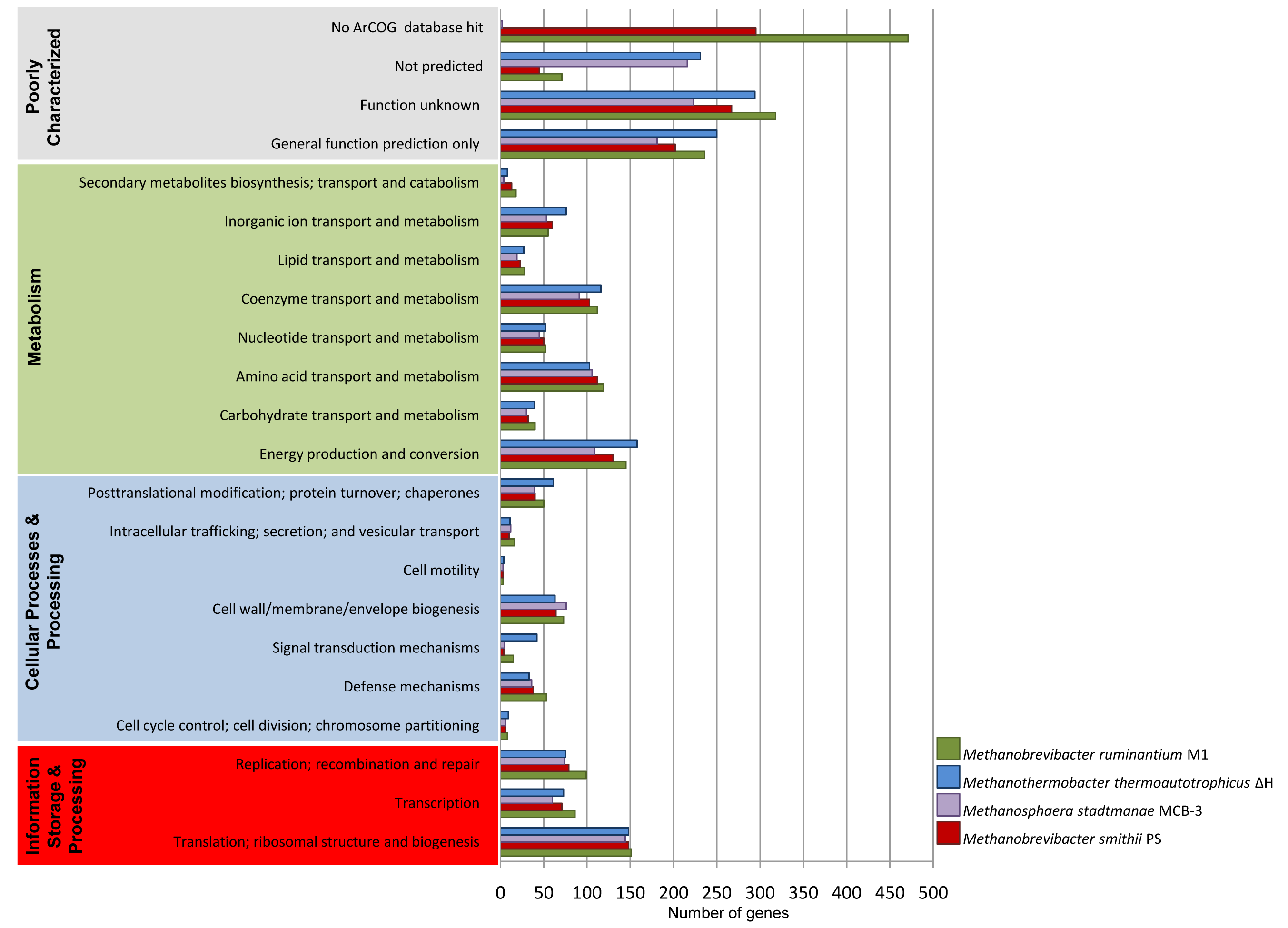

Supplement: Figure S2 — Distribution of genes in the predicted ORFeomes of members of the Methanobacteriales. ORFs are classified according to functional categories in the archaeal COG database [S1]. (0.36 MB TIF) [file pone.0008926.s009.tif]

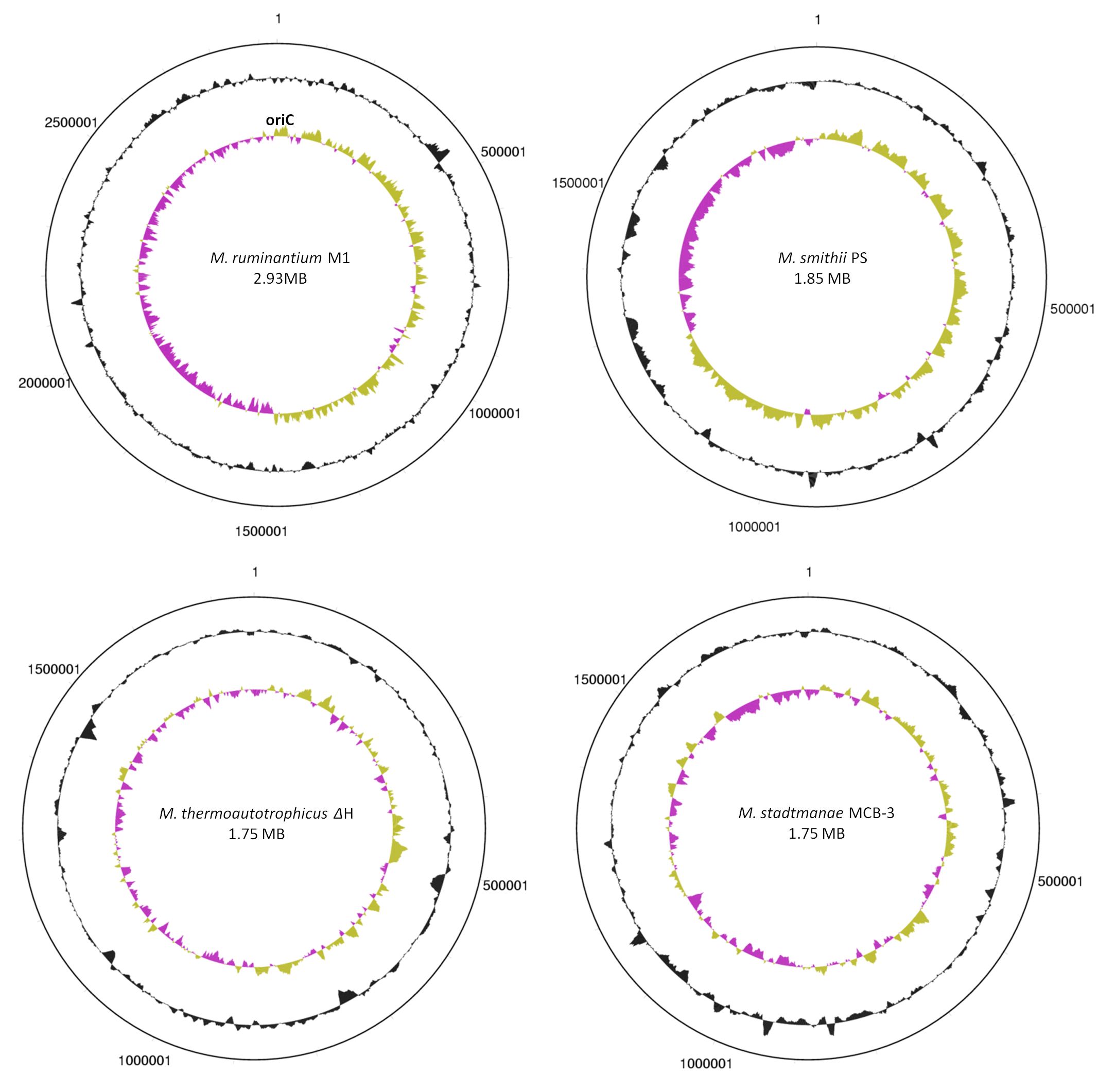

Supplement: Figure S3 — GC analysis. Base-pair scale (outer circle), G+C content (middle circle) and GC skew (inner circle, (G-C/G+C), green indicates values >1, purple <1). Genomes of members of the Methanobacteriales display a DNA skew similar to bacterial chromosomes. In M1, the origin of replication (oriC) was identified as being immediately upstream of the cdc6-1 gene (mru0001), based on GC skew analysis and homology to the origin of replication experimentally verified for M. thermoautotrophicus [S2]. As with genomes from related methanogens, M1 contains a second cdc6-2 homolog (mru0423). It also contains a truncated third cdc6-3 homolog (mru0259) within the prophage sequence. (0.93 MB TIF) [file pone.0008926.s010.tif]

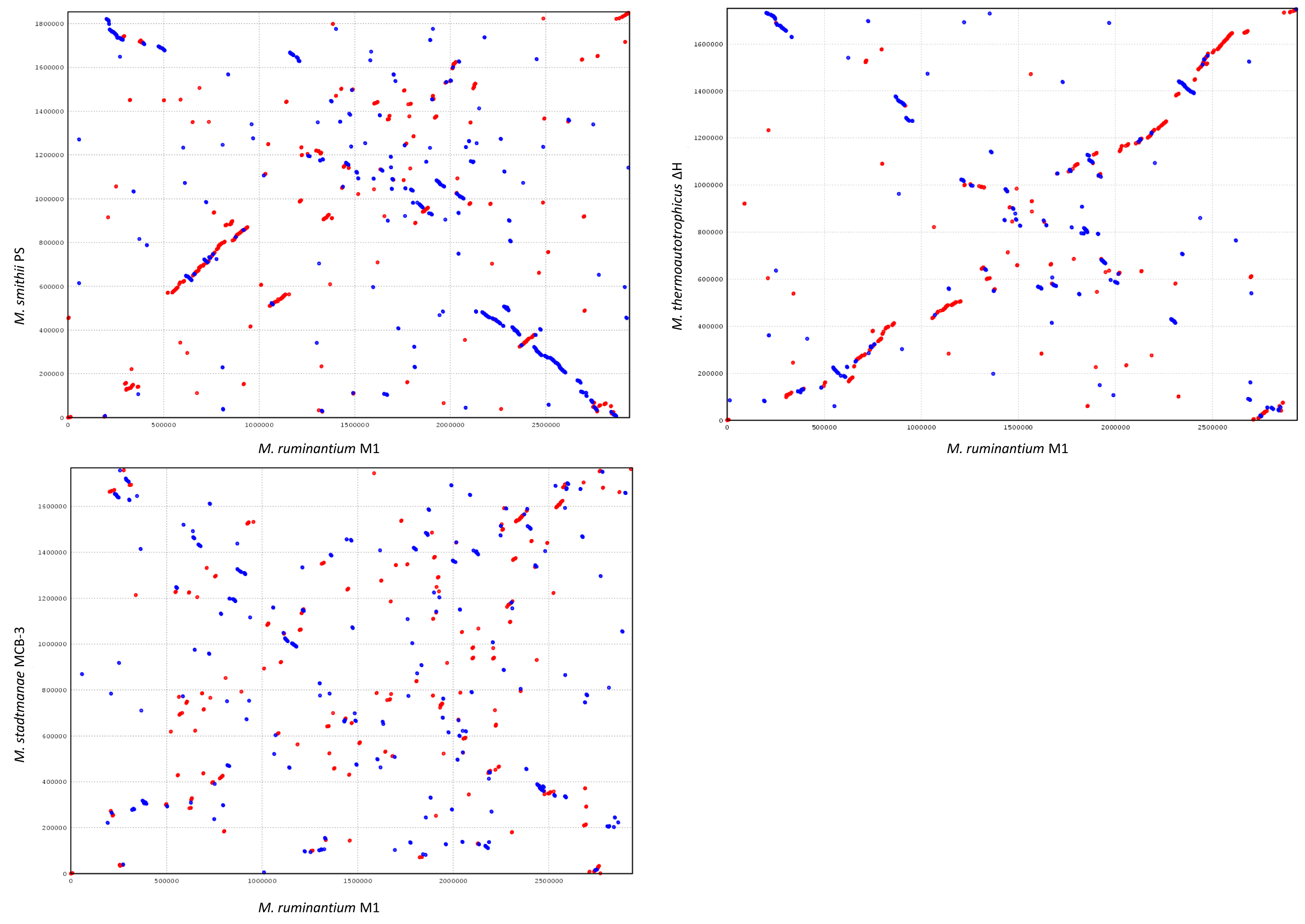

Supplement: Figure S4 — Synteny analysis. PROmer [S3] alignment of the genome of M1 against genomes from members of the Methanobacteriales. Whenever the two sequences agree, a coloured line or dot is plotted. The forward matches are displayed in red, while the reverse matches are plotted in blue. If the two sequences were perfectly identical, a single red line would go from the bottom left to the top right. An X-shape pattern is visible is all three synteny plots. It has been proposed that the X-pattern is generated by symmetric chromosomal inversions around the origin of replication [S4]. Units displayed in base-pairs. (0.49 MB TIF) [file pone.0008926.s011.tif]

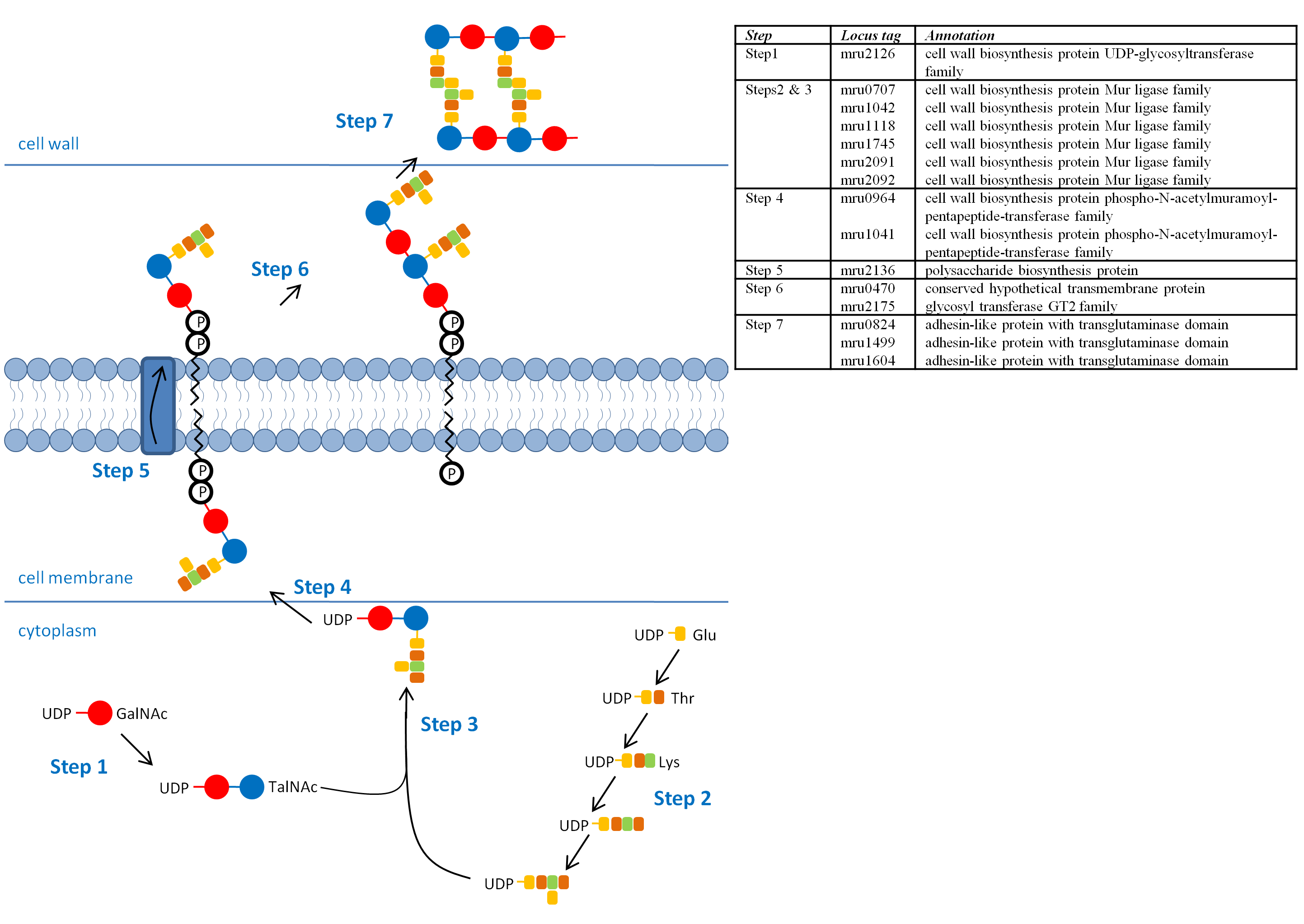

Supplement: Figure S6 — Proposed biosynthetic pathway for pseudomurein in M1 [S63, S266]. The disaccharide backbone of M1 pseudomurein consists of N-acetylgalactosamine (GalNAc) and N-acetyltalosaminuronic acid (TalNAc) in a β(1–3) linkage. TalNac has not been detected as a monomer and it is believed to be formed during the synthesis of the disaccharide probably by epimerization and oxidation of UDP-GalNAc (Step 1). Synthesis of the pentapeptide involved in crosslinking is believed to start with UDP-glutamic acid followed by stepwise addition of L-amino acids (Step 2). The amino acids found in the pentapeptide are usually alanine, lysine (Lys) and glutamic acid (Glu), but M1 is reported to contain threonine (Thr) instead of alanine [S267]. The UDP activated pentapetide is linked to the disaccharide to give a UDP-disaccharide pentapeptide (Step 3) which is subsequently translocated to the membrane via covalent bond formation with a membrane embedded undecaprenyl phosphate (Step 4). Following their intracellular biosynthesis the pseudomurein repeating units must be exported and assembled. Homologues of the Escherichia coli peptidoglycan lipid II flippase (MurJ) have been reported for pseudomurein producing methanogens [S65] (Step 5), but there are no genes similar to the penicillin binding proteins that carry out the transglycosylation (Step 6) and transpeptidation reactions in bacterial peptidoglycan assembly. Peptide crosslinking of pseudomurein requires removal of a terminal residue of one peptide and linkage from a glutamic acid to the lysine of an adjacent peptide (Step 7), and is probably carried out by transglutaminases. None of the enzymes involved in pseudomurein biosynthesis have been characterized, but analysis of the genome sequence has suggested candidates to carry out several of the steps. Several of these have homologues only in those methanogens with pseudomurein-containing cell walls. Two other transmembrane proteins of unknown function (mru1585 and mru1635) are also only fo [file pone.0008926.s013.tif]

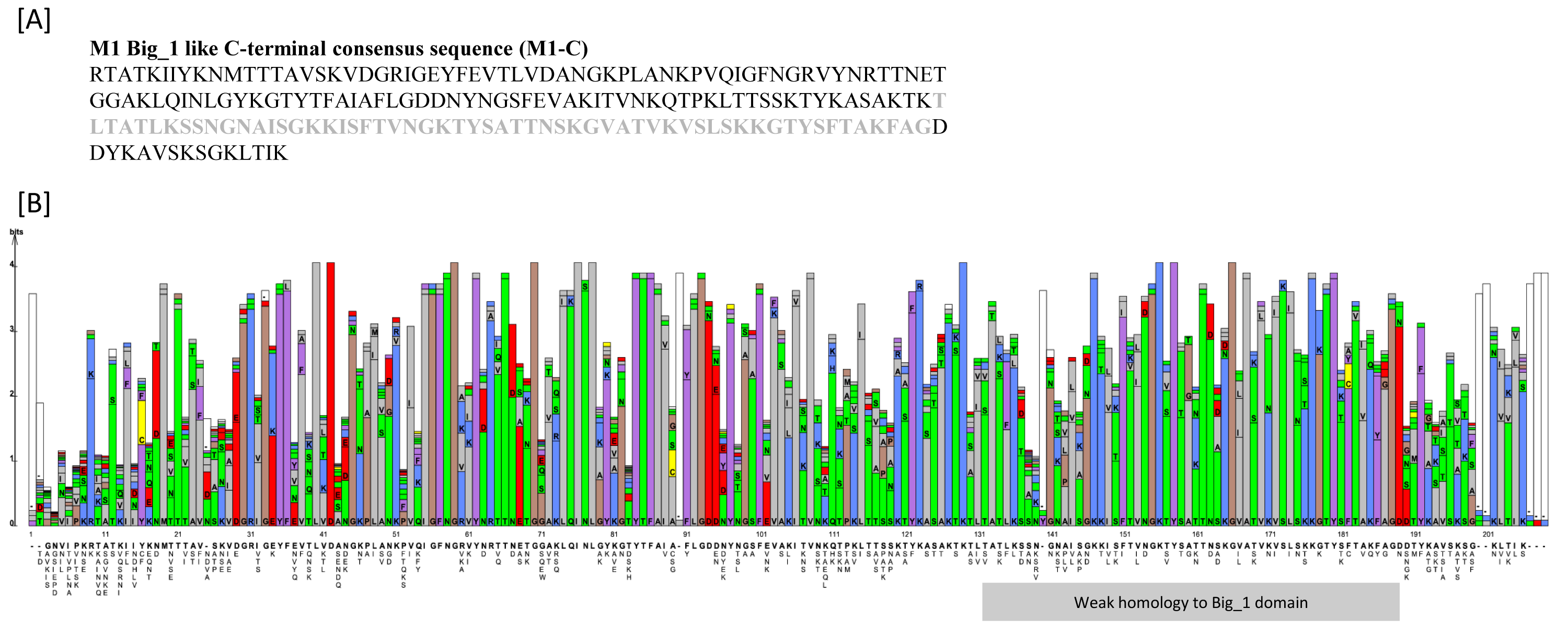

Supplement: Figure S7 — M1 M1-C domain. (A) Consensus sequence of forty-four C-terminal regions (200 amino acids) from adhesin-like proteins of M1 (M1-C). (B) LogoBar [S268] display of this consensus. In both figures the region of homology to Big_1 domain (PF02369) is highlighted in grey. (1.27 MB TIF) [file pone.0008926.s014.tif]

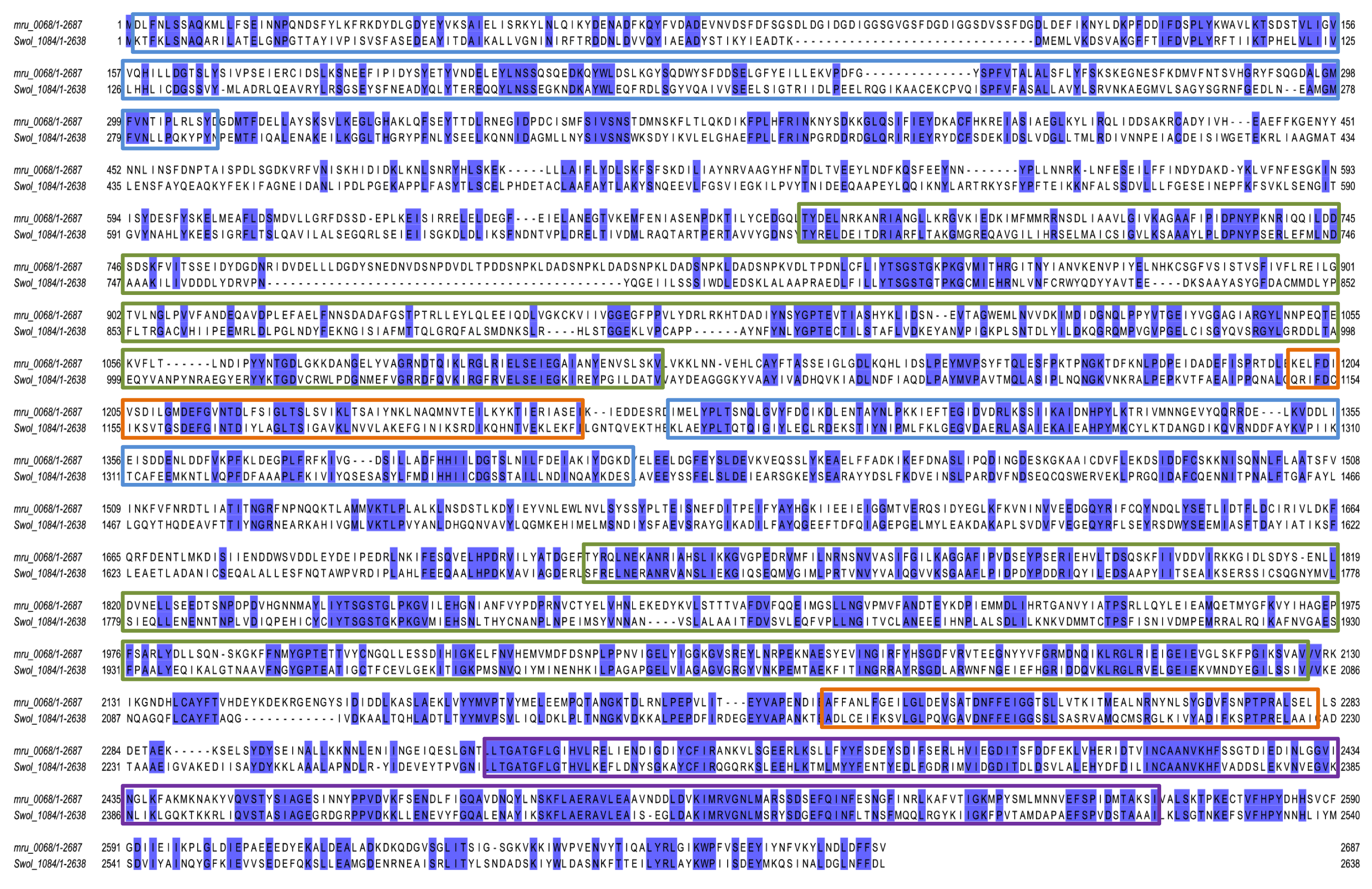

Supplement: Figure S8 — ClustalW [S269] alignment of non-ribosomal peptide synthetases from M1 (mru0068) and Syntrophomonas wolfei subsp. wolfei str. Goettingen (swol1094). Alignment was visualized using Jalview [S270]. Conserved residues are shown in blue. NRPS domain organisation of M1 is displayed via coloured boxes (light blue -condensation domain; green - adenylation domain; orange - phosphopantetheine attachment site; purple - thioester reductase domain). (2.50 MB TIF) [file pone.0008926.s015.tif]

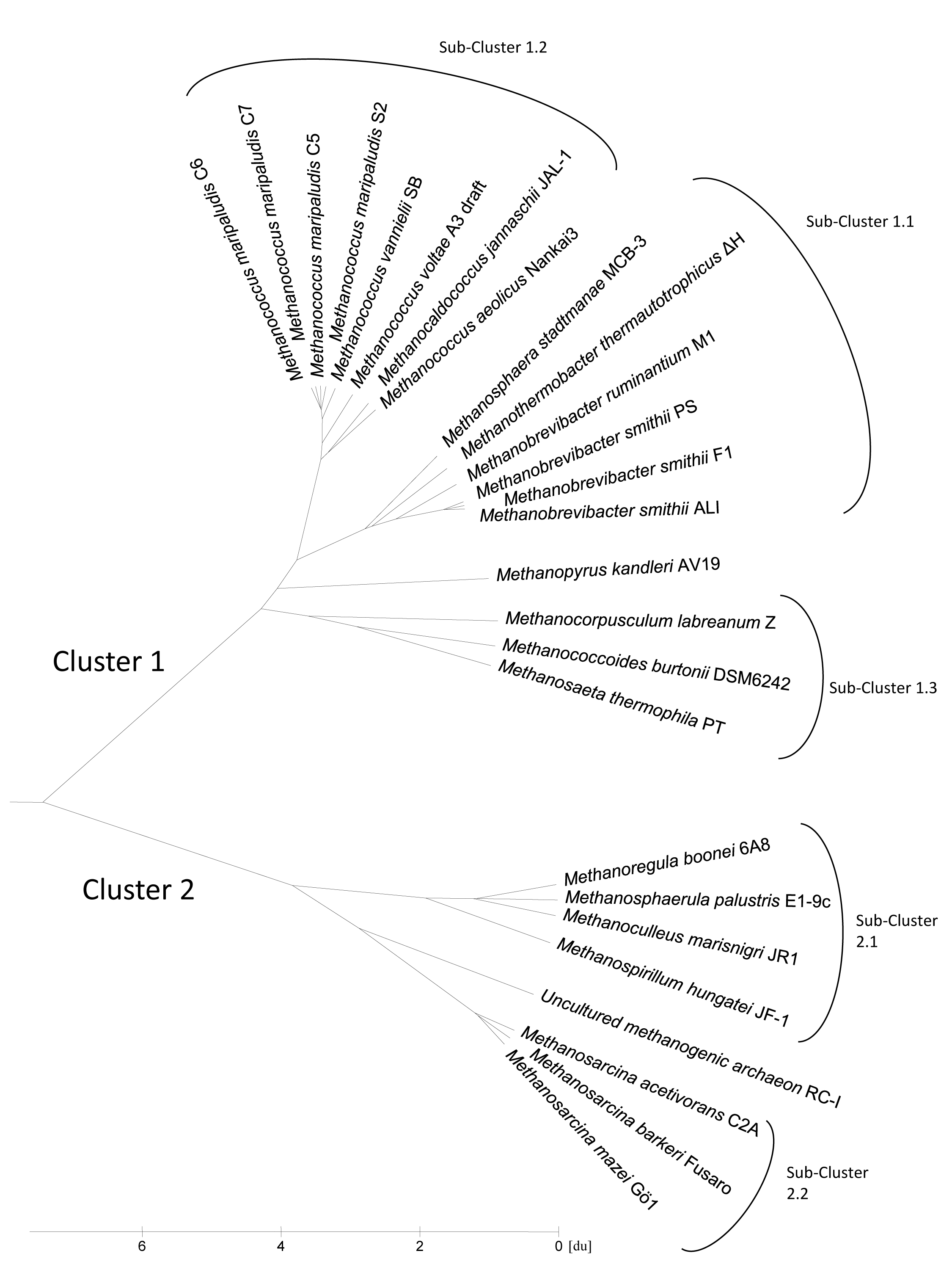

Supplement: Figure S9 — Functional Genome Distribution of 26 methanogen genomes. Publicly available complete genomes were downloaded in GenBank format where possible. Publicly available draft phase genomes were downloaded in FASTA format, concatenated using a universal spacer-stop-spacer sequence (TTAGTTAGTTAG) and automatically annotated using GAMOLA. Predicted ORFeomes of all genomes were subjected to an FGD analysis and the resulting distance matrix was imported into MEGA4 [S6]. The functional distribution was visualized using the UPGMA method [S7]. The optimal tree with the sum of branch length = 49.7 is shown. The tree is drawn to scale, with branch lengths in the same units as those of the functional distances used to infer the distribution tree. Accession numbers for individual genomes can be found in Table S7. (0.39 MB TIF) [file pone.0008926.s016.tif]
