# Supplementary material for: The Genome Sequence of the Rumen Methanogen Methanobrevibacter ruminantium Reveals New Possibilities for Controlling Ruminant Methane Emissions
Source: PLoS One. 2010 Jan 28;5(1):e8926. doi: 10.1371/journal.pone.0008926 (PMC2812497; doi:10.1371/journal.pone.0008926)
Supplement: References S1 — (0.18 MB DOC) [file pone.0008926.s017.doc]

**SUPPLEMENTARY REFERENCES**

S1. Makarova KS, Sorokin AV, Novichkov PS, Wolf YI, Koonin EV (2007) Clusters of orthologous genes for 41 archaeal genomes and implications for evolutionary genomics of archaea. Biol Direct 27:2-33.

S2. Majernik AI, Chong JP (2008) A conserved mechanism for replication origin recognition and binding in archaea. Biochem J 409:511-518.

S3. Delcher AL, Salzberg SL, Phillippy AM (2003) Using MUMmer to identify similar regions in large sequence sets. Curr. Protoc. Bioinformatics, Chapter 10:Unit 10.3.

S4. Eisen JA, Heidelberg JF, White O, Salzber SL (2000) Evidence for symmetric chromosomal inversions around the replication origin in bacteria. Genome Biol 1:RESEARCH0011.

S5. Podell S, Gaasterland T (2007) Darkhorse: a method for genome-wide prediction of horizontal gene transfer. Genome Biol 8:R16.

S6. Tamura K, Dudley J, Nei M, Kumar S (2007) MEGA4: Molecular Evolutionary Genetics Analysis (MEGA) software version 4.0. Mol Biol Evol 24:1596-1599.

S7. Sneath PHA, Sokal RR (1973) *Numerical Taxonomy. Freeman*, San Francisco.

S8. White RH, Xu H (2006) Methylglyoxal is an intermediate in the biosynthesis of 6-deoxy-5-ketofructose-1-phosphate: a precursor for aromatic amino acid biosynthesis in *Methanocaldococcus jannaschii*. Biochem 45:12366-12379.

S9. Porat I, Sieprawska-Lupa M, Teng Q, Bohanon FJ, White RH et al. (2006) [Biochemical and genetic characterization of an early step in a novel pathway for the biosynthesis of aromatic amino acids and p-aminobenzoic acid in the archaeon *Methanococcus maripaludis*.](http://www.ncbi.nlm.nih.gov/pubmed/17010158?ordinalpos=4&itool=EntrezSystem2.PEntrez.Pubmed.Pubmed_ResultsPanel.Pubmed_DefaultReportPanel.Pubmed_RVDocSum) Mol Microbiol 62:1117-31.

S10. White RH (2004) L-aspartate semialdehyde and a 6-deoxy-5-ketohexose 1-phosphate are the precursors to the aromatic amino acids on *Methanocaldococcus jannaschii*. Biochem 43:7618-7627.

S11. Porat I, Waters BW, Teng Q, Whitman WB (2004) The biosynthetic pathways for aromatic amino acid in the archaeon *Methanococcus maripaludis*. J Bacteriol 186:4940-4950.

S12. Morar M, White RH, Ealick SE (2007) [Structure of 2-amino-3,7-dideoxy-D-threo-hept-6-ulosonic acid synthase, a catalyst in the archaeal pathway for the biosynthesis of aromatic amino acids.](http://www.ncbi.nlm.nih.gov/pubmed/17713928?ordinalpos=3&itool=EntrezSystem2.PEntrez.Pubmed.Pubmed_ResultsPanel.Pubmed_DefaultReportPanel.Pubmed_RVDocSum) Biochem 46:10562-71.

S13. Daugherty M, Vonstein V, Overbeek R, Ostermann A (2001) Archaeal shikimate kinase, a new member of the GHMP-kinase family. J Bacteriol 183:292-300.

S14. Possot O, Gernhardt P, Klein A, Sibold L (1998) Analysis of drug resistance in the archaebacterium *Methanococcus voltae* with respect to potential use in genetic engineering. Appl Environ Microbiol 54:734-74.

S15. Lin Z, Sparling R (1998) Investigation of serine hydroxymethyltransferase in methanogens. Can J Microbiol 44:652-656.

S16. Hoyt JC, Oren A, Escalante-Semerena JC, Wolfe RS (1986) Tetramethanopterin-dependent serine transhydroxymethylase from *Methanobacterium thermoautotrophicum*. Arch Microbiol 145, 153-158.

S17. Angelaccio S, Chiaraluce R, Consalvi V, Buchenau B, Giangiacomo L et al. (2003) [Catalytic and thermodynamic properties of tetrahydromethanopterin-dependent serine hydroxymethyltransferase from *Methanococcus jannaschii*.](http://www.ncbi.nlm.nih.gov/pubmed/12902326?ordinalpos=5&itool=EntrezSystem2.PEntrez.Pubmed.Pubmed_ResultsPanel.Pubmed_DefaultReportPanel.Pubmed_RVDocSum) J Biol Chem 278:41789-97.

S18. Sment KA, Konisky J (1989) Excretion of amino acids by 1,2,4-triazole-3-alanine-resistant mutants of *Methanococcus voltae*. Appl Environ Microbiol 55:1295-1297.

S19. Hutton CA, Perugini MA, Gerrard JA (2007) Inhibition of lysine biosynthesis: an evolving antibiotic strategy. Mol Biosyst 3:458-465.

S20. Born TL, Blanchard JS (1999) Structure/function studies on enzymes in the diaminopimelate pathway of bacterial cell wall biosynthesis. Curr Opin Chem Biol 3:607-613.

S21. Girodeau J-M, Agouridas C, Masson M, Pineau R, Le Goffic F (1986) The lysine pathway as target for a new genera of synthetic antibacterial antibiotics? J Med Chem 29:1023-1030.

S22. Pillai B, Cherney MM, Diaper CM, Sutherland A, Blanchard JS et al. (2006) [Structural insights into stereochemical inversion by diaminopimelate epimerase: an antibacterial drug target.](http://www.ncbi.nlm.nih.gov/pubmed/16723397?ordinalpos=20&itool=EntrezSystem2.PEntrez.Pubmed.Pubmed_ResultsPanel.Pubmed_DefaultReportPanel.Pubmed_RVDocSum) Proc Natl Acad Sci *USA* 103, 8668-73.

S23. Tolbert WD, Graham DE, White RH, Ealick SE (2003) [Pyruvoyl-dependent arginine decarboxylase from *Methanococcus* *jannaschii*: crystal structures of the self-cleaved and S53A proenzyme forms.](http://www.ncbi.nlm.nih.gov/pubmed/12623016?ordinalpos=6&itool=EntrezSystem2.PEntrez.Pubmed.Pubmed_ResultsPanel.Pubmed_DefaultReportPanel.Pubmed_RVDocSum) Struct 11:285-94.

S24. Graham DE, Xu H, White RH (2002) [*Methanococcus jannaschii* uses a pyruvoyl-dependent arginine decarboxylase in polyamine biosynthesis.](http://www.ncbi.nlm.nih.gov/pubmed/11980912?ordinalpos=7&itool=EntrezSystem2.PEntrez.Pubmed.Pubmed_ResultsPanel.Pubmed_DefaultReportPanel.Pubmed_RVDocSum) J Biol Chem 28:277, 23500-23507.

S25. Kalyuzhnaya MG, Korotkova N, Crowther G, Marx CJ, Lidstrom ME et al. (2005) [Analysis of gene islands involved in methanopterin-linked C1 transfer reactions reveals new functions and provides evolutionary insights.](http://www.ncbi.nlm.nih.gov/pubmed/15968072?ordinalpos=72&itool=EntrezSystem2.PEntrez.Pubmed.Pubmed_ResultsPanel.Pubmed_DefaultReportPanel.Pubmed_RVDocSum) J Bacteriol 187:4607-4614.

S26. Xing RY, Whitman WB (1987) Sulfometuron methyl-sensitive and –resistant acetolactate synthases of the archaebacteria *methanococcus* spp. J Bacteriol 169:4486-4492.

S27. Tan S, Evans R, Singh B (2006) Herbicidal inhibitors of amino acid biosynthesis and herbicide-tolerant crops. Amino Acids 30:195-204.

S28. Hernández-Montes G, Díaz-Mejía JJ, Pérez-Rueda E, Segovia L (2008) The hidden universal distribution of amino acid biosynthetic networks: a genomic perspective in their origins and evolution. Genom Biol 9:R95.

S29. Howell DM, Xu H, White RH (1999) (*R*)-citramalate synthase in methanogenic Archaea. J Bacteriol 181:331-333.

S30. Huang Q, Tonge PJ, Slayden RA, Kirikae T, Ojima I (2007) FtsZ: a novel target for tuberculosis drug discovery. Curr Top Med Chem 7:527-543.

S31. Löwe J, Amos LA (1998) Crystal structure of the bacterial cell-division protein FtsZ. Nature 391:203-206.

S32. Ishino Y, Cann IKO (1998) The Euryarchaeotes, a subdomain of Archaea, survive on a single DNA polymerase: Fact or farce? Genes Genet Syst 73:323-336.

S33. Graille M, Cladière L, Durand D, Lecointe F, Gadelle D et al. (2008) [Crystal structure of an intact type II DNA topoisomerase: insights into DNA transfer mechanisms.](http://www.ncbi.nlm.nih.gov/pubmed/18334211?ordinalpos=4&itool=EntrezSystem2.PEntrez.Pubmed.Pubmed_ResultsPanel.Pubmed_DefaultReportPanel.Pubmed_RVDocSum) Struct 16:360-370.

S34. Gadelle D, Bocs C, Graille M, Forterre P (2005) [Inhibition of archaeal growth and DNA topoisomerase VI activities by the Hsp90 inhibitor radicicol.](http://www.ncbi.nlm.nih.gov/pubmed/15849317?ordinalpos=20&itool=EntrezSystem2.PEntrez.Pubmed.Pubmed_ResultsPanel.Pubmed_DefaultReportPanel.Pubmed_RVDocSum) Nucleic Acids Res 33:2310-2317.

S35. Makarova KS, Aravind L, Koonin EV (1999) A superfamily of archaeal, bacterial, and eukaryotic proteins homologous to animal transglutaminases. Pro Sci 8:1714-1719.

S36. Esposito C, Caputo I, Troncone R (2007) [New therapeutic strategies for coeliac disease: tissue transglutaminase as a target.](http://www.ncbi.nlm.nih.gov/pubmed/17979710?ordinalpos=21&itool=EntrezSystem2.PEntrez.Pubmed.Pubmed_ResultsPanel.Pubmed_DefaultReportPanel.Pubmed_RVDocSum) Curr Med Chem 14:2572-80.

S37. Griffin M, Casadio R, Bergamini CM (2002) Transglutaminases: nature’s biological glues. Biochem J 368:377-396.

S38. Yokoyama K, Nio N, Kikuchi Y (2004) Properties and applications of microbial transglutaminase. Appl Microbiol Biotechnol 64: 447-454.

S39. Iranzo M, Aguado C, Pallotti C, Cañizares JV, Mormeneo S (2002) [Transglutaminase activity is involved in *Saccharomyces cerevisiae* wall construction.](http://www.ncbi.nlm.nih.gov/pubmed/11988506?ordinalpos=6&itool=EntrezSystem2.PEntrez.Pubmed.Pubmed_ResultsPanel.Pubmed_DefaultReportPanel.Pubmed_RVDocSum) Microbiol 148:1329-34.

S40. Kato S, Kosaka T, Watanabe K (2008) [Comparative transcriptome analysis of responses of *Methanothermobacter thermoautotrophicus* to different environmental stimuli.](http://www.ncbi.nlm.nih.gov/pubmed/18036179?ordinalpos=62&itool=EntrezSystem2.PEntrez.Pubmed.Pubmed_ResultsPanel.Pubmed_DefaultReportPanel.Pubmed_RVDocSum) Environ Microbiol 10:893-905.

S41. Hartmann E, König H (1990) Comparison of the biosynthesis of the methanobacterial pseudomurein and the eubacterial murein. Nat Wissenschaft 77:472-475.

S42. Lee KN, Fesus L, Yancey ST, Girard JE, Chung SI (1985) Development of selective inhibitors of transglutaminase. J Biol Chem 260:14689-14694.

S43. Luo Y, Pfister P, Leisinger T, Wasserfallen A (2002) [Pseudomurein endoisopeptidases PeiW and PeiP, two moderately related members of a novel family of proteases produced in *Methanothermobacter* strains.](http://www.ncbi.nlm.nih.gov/pubmed/11934493?ordinalpos=40&itool=EntrezSystem2.PEntrez.Pubmed.Pubmed_ResultsPanel.Pubmed_DefaultReportPanel.Pubmed_RVDocSum) FEMS Microbiol Lett 208: 47-51.

S44. Steenbakkers PJ, Geerts WJ, Ayman-Oz NA, Keltjens JT (2006) [Identification of pseudomurein cell wall binding domains.](http://www.ncbi.nlm.nih.gov/pubmed/17427286?ordinalpos=3&itool=EntrezSystem2.PEntrez.Pubmed.Pubmed_ResultsPanel.Pubmed_DefaultReportPanel.Pubmed_RVDocSum) Mol Microbiol 62:1618-30.

S45. Divakaruni AV, Baida C, White CL, Gober JW (2007) [The cell shape proteins MreB and MreC control cell morphogenesis by positioning cell wall synthetic complexes.](http://www.ncbi.nlm.nih.gov/pubmed/17880425?ordinalpos=3&itool=EntrezSystem2.PEntrez.Pubmed.Pubmed_ResultsPanel.Pubmed_DefaultReportPanel.Pubmed_RVDocSum) Mol Microbiol 66:174-88.

S46. Osborn MJ, Rothfield L (2007) [Cell shape determination in *Escherichia coli*.](http://www.ncbi.nlm.nih.gov/pubmed/17981077?ordinalpos=2&itool=EntrezSystem2.PEntrez.Pubmed.Pubmed_ResultsPanel.Pubmed_DefaultReportPanel.Pubmed_RVDocSum) Curr Opin Microbiol 10:606-610.

S47. Daniel RA, Errington J (2003) [Control of cell morphogenesis in bacteria: two distinct ways to make a rod-shaped cell.](http://www.ncbi.nlm.nih.gov/pubmed/12809607?ordinalpos=12&itool=EntrezSystem2.PEntrez.Pubmed.Pubmed_ResultsPanel.Pubmed_DefaultReportPanel.Pubmed_RVDocSum) Cell 113:767-76.

S48. Candela T, Fouet A (2006) Poly-gamma-glutamate in bacteria. Mol. Microbiol*.* 60:1091-1098.

S49. Scorpio A, Chabot DJ, Day WA, O’brien DK, Vietri NJ et al. (2007) [Poly-gamma-glutamate capsule-degrading enzyme treatment enhances phagocytosis and killing of encapsulated *Bacillus anthracis*.](http://www.ncbi.nlm.nih.gov/pubmed/17074794?ordinalpos=15&itool=EntrezSystem2.PEntrez.Pubmed.Pubmed_ResultsPanel.Pubmed_DefaultReportPanel.Pubmed_RVDocSum) Antimicrob. Agents Chemother 51: 215-222.

S50. Smith CA (2006) Structure, function and dynamics in the *mur* family of bacterial cell wall ligases. J Mol Biol 362:640-655.

S51. Silver LL (2006) Does the cell wall of bacteria remain a viable source of targets for novel antibiotics? Biochem. Pharm 71:996-1005.

S52. Kotnik M, Anderluh PS, Preželj A (2007) [Development of novel inhibitors targeting intracellular steps of peptidoglycan biosynthesis.](http://www.ncbi.nlm.nih.gov/pubmed/17692001?ordinalpos=15&itool=EntrezSystem2.PEntrez.Pubmed.Pubmed_ResultsPanel.Pubmed_DefaultReportPanel.Pubmed_RVDocSum) Curr Pharm Des 13:2283-309.

S53. Katz AH, Caufield CE (2003) Structure-based design approaches to cell wall biosynthesis inhibitors. Curr Pharm Design 9:857-866.

S54. Zoeiby AE, Sanschagrin F, Levesque RC (2003) Structure and function of the Mur enzymes: development of novel inhibitors. Mol Microbiol 47:1-12.

S55. de Kruijff, B, van Dam V, Breukink W (2008) Lipid II: a central component in bacterial cell wall synthesis and a target for antibiotics. Prostagland Leukot Essent Fatty Acids 79:117-121.

S56. Kimura K, Bugg TD (2003) Recent advances in antimicrobial nucleoside antibiotics targeting cell wall biosynthesis. Nat Prod Rep 20:252-273.

S57. Hilpert R, Winter J, Hammes W, Kandler O (1981) The sensitivity of archaebacteria to antibiotics. Zbl Bakt Hyg I Abt Orig *C* 2:11-20.

S58. Namboori SE, Graham DE (2008) Acetamido sugar biosynthesis in the Euryarchaea. J Bacteriol 190:2987-2996.

S59. Hartmann E, König H (1990) Comparison of the biosynthesis of the methanobacterial pseudomurein and the eubacterial murein. Nat Wissenschaft 77:472-475.

S60. Guo RT, Cao R, Liang PH, Ko TP, Chang TH et al. (2007) [Bisphosphonates target multiple sites in both cis- and trans-prenyltransferases.](http://www.ncbi.nlm.nih.gov/pubmed/17535895?ordinalpos=4&itool=EntrezSystem2.PEntrez.Pubmed.Pubmed_ResultsPanel.Pubmed_DefaultReportPanel.Pubmed_RVDocSum) Proc Natl Acad Sci USA 104:10022-7.

S61. Scholte AA, Eubanks LM, Poulter CD, Vederas JC (2004) Synthesis and biological activity of isopentenyl diphosphate analogues. Bioorg Medic Chem 12:763-770.

S62. Hammes WP, Winter J, Kandler O (1979) The sensitivity of the pseudomurein-containing genus *Methanobacterium* to inhibitors of murein synthesis. Arch Microbiol 123:275-279.

S63. Kandler O, König H (1998) Cell wall polymers in Archaea (Archaebacteria). Cell Mol Life Sci 54:305-308.

S64. Bouhss A, Trunkfield AE, Bugg TD, Mengin-Lecreulx D (2008) [The biosynthesis of peptidoglycan lipid-linked intermediates.](http://www.ncbi.nlm.nih.gov/pubmed/18081839?ordinalpos=2&itool=EntrezSystem2.PEntrez.Pubmed.Pubmed_ResultsPanel.Pubmed_DefaultReportPanel.Pubmed_RVDocSum) FEMS Microbiol Rev 32:208-33.

S65. Ruiz N (2008) Bioinformatics identification of MurJ (MviN) as the peptidoglycan lipid II flippase in *Escherichia coli*. Proc Natl Acad Sci USA 105:15553-15557.

S66. Lindahl PA, Chang B (2001) The evolution of acetyl-CoA synthase. Orig Life Evolut Biosph 31:403-434.

S67. Musfeldt M, Schönheit P (2002) Novel type of ADP-forming acetyl coenzyme A synthetase in hyperthermophilic archaea: heterologous expression and characterization of isoenzymes from the sulfate reducer *Archaeoglobus fulgidus* and the methanogen *Methanococcus jannaschii*. J Bacteriol 184:636-644.

S68. Eggen RI, Geerling AC, Boshoven AB, de Vos WM (1991) [Cloning, sequence analysis, and functional expression of the acetyl coenzyme A synthetase gene from *Methanothrix soehngenii* in *Escherichia coli.*](http://www.ncbi.nlm.nih.gov/pubmed/1680850?ordinalpos=46&itool=EntrezSystem2.PEntrez.Pubmed.Pubmed_ResultsPanel.Pubmed_DefaultReportPanel.Pubmed_RVDocSum) J Bacteriol 173:6383-6389.

S69. Ragsdale SW (2003) Pyruvate ferredoxin oxidoreductase and its radical intermediate. Chem Rev 103:2333-2346.

S70. Dermouni HL, Ansorg RAM. Isolation and antimicrobial susceptibility testing of fecal strains of the archaeon *Methanobrevibacter smithii*. Chemother 47:177-183.

S71. Ansorg R, Rath P-M, Runde V, Beelen DW (2003) Influence of intestinal decontamination using metronidazole on the detection of methanogenic Archaea in bone marrow transplant recipients. Bone Marr Transplant 31:117-119.

S72. Bock A-K, Kunow J, Glasemacher J, Schönheit P (196) Catalytic properties, molecular composition and sequence alignments of pyruvate:ferredoxin oxidoreductase from the methanogenic archaeon *Methanosarcina barkeri* (strain Fusaro). Eur J Biochem 237:35-44.

S73. Lin WC, YangY-L, Whtman WB (2003) The anabolic pyruvate oxidoreductase from *Methanococcus maripaludis.* Arch Microbiol 179:444-456.

S74. Lin W, Whitman WB (2004) The importance of *porE* and *porG* in the anabolic pyruvate oxidoreductase of *Methanococcus maripaludis.* Arch Microbiol 181:68-73.

S75. Kato N, Yurimoto H, Thauer RK (2006) The physiological role of the ribulose monophosphate pathway in bacteria and archaea. Biosci Biotechnol Biochem 70:10-21.

S76 Grochowski LL, Xu H, White RH (2005) Ribose-5′-phosphate biosynthesis in *Methanocaldocoocus jannaschii* occurs in the absence of a pentose-phosphate pathway. J Bacteriol 187:7382-7389.

S77. Grochowski LL, White RH (2008) [Promiscuous anaerobes: new and unconventional metabolism in methanogenic archaea.](http://www.ncbi.nlm.nih.gov/pubmed/18096851?ordinalpos=4&itool=EntrezSystem2.PEntrez.Pubmed.Pubmed_ResultsPanel.Pubmed_DefaultReportPanel.Pubmed_RVDocSum) Ann N Y Acad Sci 1125:190-214.

S78. Kadziola A, Jepsen CH, Johansson E, McGuire J, Larsen S et al. (2005) Novel class III phosphoribosyl diphosphate synthase: structure and properties of the tetrameric, phosphate-activated, non-allosterically inhibited enzyme from *Methanocaldococcus jannaschii*. J Mol Biol 354:815-828.

S79. Martinez-Cruz LA, Dreyer MK, Boisvert DC, Yokota H, Martinez-Chantar ML et al. (2002) Crystal structure of MJ1247 protein from *Methanocaldococcus jannaschii* at 2.0 Å resolution infers a molecular function of 3-hexulose-6-phosphate isomerase. Struct 10:195-204.

S80. Goenrich M, Thauer RK, Yurimoto H, Kato N (2005) [Formaldehyde activating enzyme (Fae) and hexulose-6-phosphate synthase (Hps) in *Methanosarcina barkeri*: a possible function in ribose-5′-phosphate biosynthesis.](http://www.ncbi.nlm.nih.gov/pubmed/16075199?ordinalpos=3&itool=EntrezSystem2.PEntrez.Pubmed.Pubmed_ResultsPanel.Pubmed_DefaultReportPanel.Pubmed_RVDocSum) Arch Microbiol 184:41-48.

S81. Werken van de HJG, Brouns SJJ, Oost J van der. (2008) Pentose metabolism in archaea. In: The Archaea, new models for prokaryotic biology. (Ed.) Blum P. Caister Academic Press 71-94 p.

S82. Soderberg T (2005) Biosynthesis of ribose-5′-phosphate and erythrose-4-phosphate in archaea: a phylogenetic analysis of archaeal genomes. Archaea 1:347-352.

S83. Lee BI, Chang C, Cho SJ, Eom SH, Kim KK et al*.* (2001) Crystal structure of the MJ0490 gene product of the hyperthermophilic archaebacterium *Methanococcus* *jannaschii*, a novel member of the lactate/malate family of dehydrogenases. Biochem 40:10310-10316.

S84. Sprott GD, McKellar RC, Shaw KM, Giroux J, Martin WG (1979) Properties of malate dehydrogenase isolated from *Methanospirillum hungatei*. Can J Microbiol 25:192-200.

S85. Storer AC, Sprott GD, Martin WG (1981) Kinetic and physical properties of the L-malate-NAD+ oxidoreductase from *Methanospirillum hungatei* and comparison with the enzyme from other sources. Biochem J 193:235-244.

S86. Thompson H, Tersteegen A, Thauer RK, Hedderich R (1998) Two malate dehydrogenases in *Methanobacterium thermoautotrophicum*. Arch Microbiol 170:38-42.

S87. Mukhopadhyay B, Stoddard SF, Wolfe RS (1998) [Purification, regulation, and molecular and biochemical characterization of pyruvate carboxylase from *Methanobacterium thermoautotrophicum* strain deltaH.](http://www.ncbi.nlm.nih.gov/pubmed/9478969?ordinalpos=14&itool=EntrezSystem2.PEntrez.Pubmed.Pubmed_ResultsPanel.Pubmed_DefaultReportPanel.Pubmed_RVDocSum) J Biol Chem 273:5155-5166.

S88. Mukhopadhyay B, Patel VJ, Wolfe RS (2000) [A stable archaeal pyruvate carboxylase from the hyperthermophile *Methanococcus jannaschii.*](http://www.ncbi.nlm.nih.gov/pubmed/11195096?ordinalpos=11&itool=EntrezSystem2.PEntrez.Pubmed.Pubmed_ResultsPanel.Pubmed_DefaultReportPanel.Pubmed_RVDocSum) Arch Microbiol 174:406-414.

S89. Mukhopadhyay B, Purwantini E, Kreder CL, Wolfe RS (2001) [Oxaloacetate synthesis in the methanarchaeon *Methanosarcina barkeri*: pyruvate carboxylase genes and a putative *Escherichia coli*-type bifunctional biotin protein ligase gene (bpl/birA) exhibit a unique organization.](http://www.ncbi.nlm.nih.gov/pubmed/11371547?ordinalpos=72&itool=EntrezSystem2.PEntrez.Pubmed.Pubmed_ResultsPanel.Pubmed_DefaultReportPanel.Pubmed_RVDocSum) J Bacteriol 183:3804-3810.

S90. Shieh JS, Whitman WB (1987) [Pathway of acetate assimilation in autotrophic and heterotrophic methanococci.](http://www.ncbi.nlm.nih.gov/pubmed/3667534?ordinalpos=18&itool=EntrezSystem2.PEntrez.Pubmed.Pubmed_ResultsPanel.Pubmed_DefaultReportPanel.Pubmed_RVDocSum) J Bacteriol 169:5327-5329.

S91. Bobik TA, Wolfe RS (1989) [An unusual thiol-driven fumarate reductase in Methanobacterium with the production of the heterodisulfide of coenzyme M and N-(7-mercaptoheptanoyl)threonine-*O*^3^-phosphate.](http://www.ncbi.nlm.nih.gov/pubmed/2509466?ordinalpos=32&itool=EntrezSystem2.PEntrez.Pubmed.Pubmed_ResultsPanel.Pubmed_DefaultReportPanel.Pubmed_RVDocSum) J Biol Chem 264:18714-18718.

S92. Heim S, Künkel A, Thauer RK, Hedderich R (1998) [Thiol:fumarate reductase (Tfr) from *Methanobacterium thermoautotrophicum*--identification of the catalytic sites for fumarate reduction and thiol oxidation.](http://www.ncbi.nlm.nih.gov/pubmed/9578488?ordinalpos=19&itool=EntrezSystem2.PEntrez.Pubmed.Pubmed_ResultsPanel.Pubmed_DefaultReportPanel.Pubmed_RVDocSum) Eur J Biochem 253:292-299.

S93. Lemker T, Ruppert C, Stöger H, Wimmers S, Müller V (2001) Overproduction of a functional A_1_ ATPase from the archaeon *Methanosarcina mazei* Gö1 in *Escherichia coli*. Eur J Biochem 268:3744-3750.

S94 Lemker T, Grüber G, Schmid R, Müller V (2003) Defined subcomplexes of the A_1_ ATPase from the archaeon *Methanosarcina mazei* Gö1: biochemical properties and redox regulation. FEBS Lett 544:206-209.

S95. Lewalter K, Müller V (2006) Bioenergetics of archaea: ancient energy conserving mechanisms developed in the early history of life. Biochim Biophys Acta 1757:437-445.

S96. Schäfer IB, Bailer SM, Düser MG, Börsch M, Bernal RA (2006) Crystal structure of the archaeal A_1_A_0_ ATPase synthase subunit B from *Methanosarcina mazei* Gö1: implications of the nucleotide-binding differences in the major A_1_A_0_ subunits A and B. J Mol Biol 358:725-740.

S97. Schäfer IB, Rössle M, Biuković G, Müller V, Grüber G (2006) Structural and functional analysis of the coupling subunit F in solution and topological arrangements of the stalk domains of the methanogenic A_1_A_0_ ATP synthase. J Bioenerg Biomem 38:83-92.

S98. Coskun U, Grüber G, Koch MH, Godovac-Simmermann J, Lemker T et al. (2002) Cross-talk in the A_1_-ATPase from *Methanosarcina mazei* Gö1 due to nucleotide binding. J Biol Chem 277:17327-17333.

S99. Coskun U, Chaban YL, Lingl A, Müller V, Keegstra W et al. (2004) Structure and subunit arrangement of the A-type ATP synthase complex from the archaeon *Methanococcus jannaschii* visualized by electron microscopy. J Biol Chem 279:38644-38648.

S100. Lingl A, Huber H, Stetter KO, Mayer F, Kellermann J et al. (2003) Isolation of a complete A_1_A_0_ ATP synthase comprising nine subunits from the hyperthermophile *Methanococcus jannaschii*. Extremophiles 7:249-257.

S101. Sprott GD, Jarrell KF (1982) Sensitivity of methanogenic bacteria to dicyclohexylcarbodiimide. Can. J Microbiol 28: 982-986.

S102. Grüber G, Marshansky V (2008) New insights into structure-function relationships between archaeal ATP synthase (A_1_A_0_) and vacuolar type ATPase (V_1_V_0_). Bioessays 30:1096-1109.

S103. Pisa KY, Weidner C, Maischak H, Kavermann H, Müller V (2007) The coupling ion in the methanoarchaeal ATP synthase: H^+^ vs Na^+^ in the A_0_A_1_ ATP synthase from the archaeon *Methanosarcina mazei* Gö1. FEMS Miccrobiol Lett 277:56-63.

S104. Müller V, Ruppert C, Lemkar T (1999) Structure and function of the A_0_A_1_-ATPase from methanogenic archaea. J Bioenerg Biomembr 31:15-27.

S105. Müller V (2004) An exceptional variability in the motor of archael A_1_A_0_ ATPases: from multimeric to monomeric rotors comprising 6-13 ion binding sites. J. Bioenerg. Biomembr. 36:115-125.

S106. Ferry JG (1999) Enzymology of one-carbon metabolism in methanogenic pathways. FEMS Microbiol Rev 23:13-38.

S107. Alex LA, Reeve JN, Orme-Johnson WH, Walsh CT (1990) Cloning, sequence determination and expression of the genes encoding the subunits of the nickel-containing 8-hydroxy-5-deazaflavin reducing hydrogenase from *Methanobacterium thermoautotrophicum*. Biochem 29:7237-7244.

S108. Tersteegen A, Hedderich R (1999) *Methanobacterium thermoautotrophicum* encodes two multisubunit membrane-bound [NiFe] hydrogenases. Transcription of the operons and sequence analysis of the deduced proteins. Eur J Biochem 264: 930-943.

S109. Anderson I, Ulrich LE, Lupa B, Susanti D, Porat I et al. (2009) [Genomic characterization of Methanomicrobiales reveals three classes of methanogens.](http://www.ncbi.nlm.nih.gov/pubmed/19495416?ordinalpos=1&itool=EntrezSystem2.PEntrez.Pubmed.Pubmed_ResultsPanel.Pubmed_DefaultReportPanel.Pubmed_RVDocSum) PLoS One 4:e5797.

S110. Porat I, Kim W, Hendrickson EL, Xia Q, Zhang Y et al. (2006) [Disruption of the operon encoding Ehb hydrogenase limits anabolic CO_2_ assimilation in the archaeon *Methanococcus maripaludis*.](http://www.ncbi.nlm.nih.gov/pubmed/16452419?ordinalpos=1&itool=EntrezSystem2.PEntrez.Pubmed.Pubmed_ResultsPanel.Pubmed_DefaultReportPanel.Pubmed_RVDocSum) J Bacteriol 188:1373-1380.

S111. Woo G-J, Wasserfallen A, Wolfe RS (1993) Methyl violgen hydrogenase II, a new member of the hydrogenase family from *Methanobacterium thermoautotrophicum* ΔH. J. Bacteriol*.* 175:5970-5977.

S112. Shah NN, Clark DS (1990) Partial purification and characterization of two hydrogenases from the extreme thermophile *Methanococcus jannaschii*. Appl Environ Microbiol 56:858-863.

S113. Stojanowic A, Mander GJ, Duin EC, Hedderich R (2003) Physiological role of the F_420_-non-reducing hydrogenase (Mvh) from *Methanothermobacter marburgensis.* Arch Microbiol 180:194-203.

S114. Shima S, Warkentin E, Thauer RK, Ermler U (2002) Structure and function of enzymes involved in the methanogenic pathway utilizing carbon dioxide and molecular hydrogen. J Biosci Bioeng 93:519-530.

S115. Thauer RK, Hedderich R, Fischer R (1993) Unusual coenzymes of methanogenesis from CO_2_ and H_2_. In Ferry, J.G. (ed.) Methanogenesis: ecology, physiology, biochemistry and genetics. Chapman and Hall, New York, 209-252 p.

S116. Aufhammer SW, Warkentin E, Ermler U, Hagemeier CH, Thauer RK et al. (2005) Crystal structure of formylmethanofuran: tetrahydromethanopterin formyltransferase in complex with coenzyme F_420_: architecture of the F_420_/FMN binding site of enzymes within the nonprolyl cis-peptide containing bacterial luciferase family. Pro Sci 14:1840-1849.

S117. Hedderich R, Hamann N, Bennati M (2005) Heterodisulfide reductase from methanogenic archaea: a new catalytic role for an iron-sulfur cluster. Biol Chem 386:961-970.

S118. Mauer J, Kuettner HC, Zhang JK, Hedderich R, Metcalf WW (2002) Genetic analysis of the archaeon *Methanosarcina barkeri* reveals a central role for Ech hydrogenase and ferredoxin in methanogenesis and carbon fixation. Proc Natl Acad Sci USA 99:5632-5637.

S119. Deppenmeier U (2002) Redox-driven proton translocation in methnaogenic Archaea. Cell Mol Life Sci 59:1513-1533.

S120. Shokes JE, Duin EC, Bauer C, Jaun B, Hedderich R et al. (2005) Direct interaction of coenzyme M with the active-site Fe-S cluster of heterodisulfide reductase. FEBS Lett 579:1741-1744.

S121. De Poorter LMI, Geerts WG, Theuvenet AP, Keltjens JT (2003) [Bioenergetics of the formyl-methanofuran dehydrogenase and heterodisulfide reductase reactions in *Methanothermobacter thermoautotrophicus*.](http://www.ncbi.nlm.nih.gov/pubmed/12492476?ordinalpos=4&itool=EntrezSystem2.PEntrez.Pubmed.Pubmed_ResultsPanel.Pubmed_DefaultReportPanel.Pubmed_RVDocSum) Eur J Biochem 270:66-75.

S122. Schäfer IB, Engelhard M, Müller V (1999) Bioenergetics of the Archaea. Microbiol Mol Biol Rev 63:570-620.

S123. Pilak O, Mamat B, Vogt S, Hagemeier CH, Thauer Rk et al. (2006) The crystal structure of the apoenzyme of the iron-sulphur cluster-free hydrogenase. J Mol Biol 358:798-809.

S124. Shima S, Pilak O, Vogt S, Schick M, Stagni MS et al. (2008) [The crystal structure of [Fe]-hydrogenase reveals the geometry of the active site.](http://www.ncbi.nlm.nih.gov/pubmed/18653896?ordinalpos=3&itool=EntrezSystem2.PEntrez.Pubmed.Pubmed_ResultsPanel.Pubmed_DefaultReportPanel.Pubmed_RVDocSum) Science 321:572-5.

S125. Vignais PM, Billoud B, Meyer J (2001) Classification and phylogeny of hydrogenases. FEMS Microbiol Rev 25:455-501.

S126. Hendrickson EL, Leigh JA (2008) Roles of coenzyme F_420_-reducing hydrogenases and hydrogen- and F_420_-dependent methylenetetrahydromethanopterin dehydrogenases in reduction of F_420_ and production of hydrogen during methanogenesis. J Bacteriol 190:4818-4821.

S127. Klein AR, Fernández VM, Thauer RK (1995) H_2_-forming *N*^5^-*N*^10^-methylenetetrahydromethanopterin dehydrogenase: mechanism of H_2_ formation analyzed using hydrogen isotopes. FEBS Lett 368:203-206.

S128. Hagemeier CH, Shima S, Thauer RK, Bourenkov G, Bartunik HD et al. (2003) Coenzyme F420-dependent methylenetetrahydromethanopterin dehydrogenase [(Mtd) from *Methanopyrus kandleri*: a methanogenic enzyme with an unusual quarternary structure.](http://www.ncbi.nlm.nih.gov/pubmed/14499608?ordinalpos=10&itool=EntrezSystem2.PEntrez.Pubmed.Pubmed_ResultsPanel.Pubmed_DefaultReportPanel.Pubmed_RVDocSum) J Mol Biol 332:1047-1057.

S129. Mukhopadhyay B, Daniels L (1989) Aerobic purification of N^5^, N^10^-methenyltetrahydromethanopterin dehydrogenase. Separated from N^5^, N^10^-methenyltetrahydromethanopterin cyclohydrolase, from *Methanobacterium thermoautotrophicum* strain Marburg. Can J Microbiol 35:499-507.

S130. Mukhopadhyay B, Purwantini E, Pihl TD, Reeve JN, Daniels L (1995) [Cloning, sequencing, and transcriptional analysis of the coenzyme F_420_-dependent methylene-5,6,7,8-tetrahydromethanopterin dehydrogenase gene from *Methanobacterium* *thermoautotrophicum* strain Marburg and functional expression in *Escherichia coli*.](http://www.ncbi.nlm.nih.gov/pubmed/7852356?ordinalpos=92&itool=EntrezSystem2.PEntrez.Pubmed.Pubmed_ResultsPanel.Pubmed_DefaultReportPanel.Pubmed_RVDocSum) J Biol Chem 270:2827-2832.

S131. Jacobson FS, Daniels L, Fox JA, Walsh CT, Orme-Johnson WH (1982) Purification and properties of an 8-hydroxy-5-deazaflavin-reducing hydrogenase from *Methanobacterium thermautotrophicum*. JBiol Chem 257:3385-3388.

S132. Acharya P, Warkentin E, Ermler U, Thauer RK, Shima S (2006) The structure of formylmethanofuran: tetrahydromethanopterin formyltransferase in complex with its coenzymes. J Mol Biol 357:870-879.

S133. Mamat B, Roth A, Grimm C, Ermler U, Tziatzios C et al. (2002) Crystal structures and enzymatic properties of three formyltransferases from archaea: environmental adaptation and evolutionary relationship. Pro Sci 11:2168-2178.

S134. DiMarco AA, Donnelly MI, Wolfe RS (1986) Purification and properties of the 5,10-methenyltetrahydromethanopterin cyclohydrolase from *Methanobacterium thermoautotrophicum*. J Bacteriol 168:1372-1377.

S135. Donnelly MI, Escalante-Semerena JC, Rinehart KL Jr, Wolfe RS (1985) Methenyl-tetrahydromethanopterin cyclohydrolase in cell extracts of *Methanobacterium.* Arch Biochem Biophys 242:430-439.

S136. Vaupel M, Dietz H, Linder D, Thauer RK (1996) Primary structure of cyclohydrolase (Mch) from *Methanobacterium thermoautotrophicum* (strain Marburg) and functional expression of the *mch* gene in *Escherichia coli*. Eur J Biochem 236:294-300.

S137. Whitman WB, Wolfe RS (1985) Activation of the methylreductase system from *Methanobacterium bryantii* by corrins, J Bacteriol 164:165-172.

S138. Whitman WB, Wolfe RS (1987) Inhibition by corrins of the ATP-dependent activation and the CO_2_ reduction by the methylreductase system in *Methanobacterium bryantii*. J Bacteriol 169:87-92.

S139. Harmer J, Finazzo C, Piskorski R, Ebner S, Duin EC et al. (2008) A nickel hydride complex in the active site of methyl-coenzyme M reductase: implications for the catalytic cycle. J Am Chem Soc 130:10907-10920.

S140. Ermler U (2005) On the mechanism of methyl-coenzyme reductase. Dalton Trans 21:3451-3458.

S141. Grabarse W, Mahlert F, Duin EC, Goubeaud M, Shima S et al. On the mechanism of biological methane formation: structural evidence for conformational changes methyl-coenzyme M reductase upon substrate binding. J Mol Biol 309:315-330.

S142. Selmer T, Kahnt J, Goubeaud M, Shima S, Grabarse W et al. The biosynthesis of methylated amino acids in the active site region of methyl-coenzyme M reductase. J Biol Chem 275:3755-3760.

S143. Ermler U, Grabarse W, Shima S, Goubeaud M, Thauer RK (1997) Crystal structure of methyl –coenzyme reductase: the key enzyme of biological methane formation. Science 278:1457-1462.

S144. Prins RA, van Nevel CJ, Demeyer DI (1972) Pure culture studies of inhibitors for methanogenic bacteria. Antonie Van Leeuwenhoek 38:281-287.

S145. Attwood G, McSweeney C (2008) Methanogen genomics to discover targets for methane mitigation technologies and options for alternative H_2_ utilisation in the rumen. Aust J Exper Agric 48:28-37.

S146. Rospert S, Voges M, Berkessel A, Albracht SPJ, Thauer RK (1992) Substrate-induced changes in the nickel-EPR spectrum of active methyl-coenzyme-M reductase from *Methanobacterium thermoautotrophicum*. Eur J Biochem 210: 101-107.

S147. Goenrich M, Mahlert F, Duin EC, Bauer C, Jaun B et al. (2004) Probing the reactivity of Ni in the active site of methyl-coenzyme M reductase with substrate analogues*.* J. Biol. Inorg. Chem. 9:691-705.

S148. Buckel W, Golding BT (2006) Radical enzymes in anaerobes. Annu Rev Microbiol 60:27-49.

S149. Ellermann J, Hedderich R, Bocher R, Thauer RK (1988) The final step in methane formation. Investigations with highly purified methyl-CoM reducatse (component C) from *Methanobacterium thermoautotrophicum* (strain Marburg). Eur J Biochem 172:669-677.

S150. Sauer FD (1991) Inhibition of methylcoenzyme M methylreductase by a uridine 5′-diphospho-acetylglucosamine derivative. *Biochem. Biophys.* Res Comm 174:619-624.

S151. Wackett LP, Honek JF, Begley TP, Wallace V, Orme-Johnson WH *et al.* Substrate analogues as mechanistic probes of methyl-S-coenzyme M reductase. Biochem 26:6012-6018.

S152. Gottschalk G, Thauer RK (2001) The Na^+^-translocating methyltransferase complex from methanogenic archaea. Biochim Biophys Acta 1505:28-36.

S153. Kenealy W, Zeikus JG (1993) Influence of corrinoid antagonists on methanogen metabolism. J Bacteriol 146:133-140.

S154. Stupperich R (1993) Recent advances in elucidation of biological corrinoid functions. FEMS Microbiol Rev 12:349-366.

S155. Becher B, Muller V, Gottschalk G (1992) N^5^-methyl-tetrahydromethanopterin:coenzyme M methyltransferase of *Methanosarcina* strain Gö1 is an Na(+)-translocating membrane protein. J Bacteriol 174:7656-7660.

S156. Andreesen JR, Makdessi K (2008) [Tungsten, the surprisingly positively acting heavy metal element for prokaryotes.](http://www.ncbi.nlm.nih.gov/pubmed/18096847?ordinalpos=1&itool=EntrezSystem2.PEntrez.Pubmed.Pubmed_ResultsPanel.Pubmed_DefaultReportPanel.Pubmed_RVDocSum) Ann N Y Acad Sci 1125:215-229.

S157. Hochheimer A, Schmitz RA, Thauer RK, Hedderich R (1995) [The tungsten formylmethanofuran dehydrogenase from *Methanobacterium thermoautotrophicum* contains sequence motifs characteristic for enzymes containing molybdopterin dinucleotide.](http://www.ncbi.nlm.nih.gov/pubmed/8575452?ordinalpos=16&itool=EntrezSystem2.PEntrez.Pubmed.Pubmed_ResultsPanel.Pubmed_DefaultReportPanel.Pubmed_RVDocSum) Eur J Biochem 234:910-920.

S158. Hochheimer A, Linder D, Thauer RK, Hedderich R (1996) The molybdenum formylmethanofuran dehydrogenase operon and the tungsten formylmethanofuran dehydrogenase operon from *Methanobacterium thermoautotrophicum*. Structures and transcriptional regulation. Eur J Biochem 242:156-162.

S159. Hochheimer A, Hedderich R, Thauer RK (1998) [The formylmethanofuran dehydrogenase isoenzymes in *Methanobacterium wolfei* and *Methanobacterium thermoautotrophicum*: induction of the molybdenum isoenzyme by molybdate and constitutive synthesis of the tungsten isoenzyme.](http://www.ncbi.nlm.nih.gov/pubmed/9818358?ordinalpos=29&itool=EntrezSystem2.PEntrez.Pubmed.Pubmed_ResultsPanel.Pubmed_DefaultReportPanel.Pubmed_RVDocSum) Arch Microbiol 170:389-393.

S160. Deppenmeier U (2002) The unique biochemistry of methanogens. Prog Nucl Acid Res Mol Biol 71:223-283.

S161. Vorholt JA (1997) The active species of ‘CO_2_’ utilized by formylmethanofuran dehydrogenase from methanogenic Archaea. Eur J Biochem 248:919-924.

S162. Wasserfallen A (1994) [Formylmethanofuran synthesis by formylmethanofuran dehydrogenase from *Methanobacterium* *thermoautotrophicum* Marburg.](http://www.ncbi.nlm.nih.gov/pubmed/8147868?ordinalpos=12&itool=EntrezSystem2.PEntrez.Pubmed.Pubmed_ResultsPanel.Pubmed_DefaultReportPanel.Pubmed_RVDocSum) Biochem Biophys Res Comm 199:1256-1261.

S163. Heath RJ, Rock CO (2004) Fatty acid biosynthesis as a target for novel antibacterials. Curr Opin Investig Drugs 5:146-153.

S164. Heath RJ, White SW, Rock CO (2001) Lipid biosynthesis as a target for antibacterial agents. Prog Lipid Res 40:467-497.

S165. Campbell JW, Cronan Jr JE (2001) Bacterial fatty acid biosynthesis: targets for antibacterial drug discovery. Annu Rev Microbiol 55:305-332.

S166. Payne DJ, Warren PV, Holmes DJ, Ji Y, Lonsdale JT (2001) Bacterial fatty acid biosynthesis: a genomics-driven target for antibacterial discovery. Drug Disc Ther 6:537-544.

S167. Payne DJ (2008) Desperately seeking new antibiotics. Science 321:1644-1645.

S168. Daiyasu H, Hiroike T, Koga Y, Toh H (2002) Analysis of membrane stereochemistry with homology modeling of *sn*-glycerol-1-phosphate dehydrogenase. Prot Eng 15:987-995.

S169. Koga Y, Morii H (2007) Biosynthesis of ether-linked polar lipids in archaea and evolutionary considerations. Microbiol Mol Biol Rev 71:97-120.

S170. Miller TL, Wolin MJ (2001) Inhibition of growth of methane-producing bacteria of the rumen forestomach by hydroxymethyl-SCoA reductase inhibitors. J Dairy Sci 84:1445-1448.

S171. Samuel BS, Hansen EE, Manchester JK, Coutinho PM, Henrissat B et al (2007) Genomic and metabolic adaptations of *Methanobrevibacter smithii* to the human gut. Proc Natl Acad Sci USA 104:10643-10648.

S172. de Ruyck J, Wouters J (2008) Structure-based design targeting biosynthesis of isoprenoids: a crystallographic state of the art of the involved enzymes. Curr Pro Pep Sci 9:117-137.

S173. Bonanno JB, Edo C, Eswar N, Pieper U, Romanowski MJ et al. Structural genomics of enzymes involved in sterol/isoprenoid biosynthesis. Proc Natl Acad Sci USA 98:12896-12901.

S174. Friesen JA, Rodwell JA (2004) The 3-hydroxy-3-methylglutaryl coenzyme-A (HMG-CoA) reductases. Genome Biol 5:248.

S175. Istvan ES (2001) Bacterial and mammalian HMG-CoA reductases: related enzymes and distinct architectures. Curr Opin Struct Biol 11:746-751.

S176. Smit A, Mushegian A (2000) Biosynthesis of isoprenoid via mevalonte in archaea: the lost pathway. Genom Res 10: 1465-1484.

S177. Boucher Y, Kamekura M, Doolittle WF (2004) Origins and evolution of isoprenoid lipid biosynthesis in archaea. Mol Microbiol 52:515-527.

S178. Barkley SJ, Cornish RM, Poulter CD (2004) [Identification of an Archaeal type II isopentenyl diphosphate isomerase in *Methanothermobacter* *thermoautotrophicus*.](http://www.ncbi.nlm.nih.gov/pubmed/14996812?ordinalpos=3&itool=EntrezSystem2.PEntrez.Pubmed.Pubmed_ResultsPanel.Pubmed_DefaultReportPanel.Pubmed_RVDocSum) J Bacteriol 186:1811-1817.

S179. Hoshino T, Tamegai H, Kakinuma K, Eguchi T (2006) Inhibition of type 2 isopentenyl diphosphate isomerase from *Methanocaldococcus jannaschii* by a mechanism-based inhibitor of type I1 isopentenyl diphosphate isomerase. Bioorg Med Chem 14:6555-6559.

S180. Wouters J, Oudjama Y, Stalon V, Droogmans L, Poulter CD (2004) [Crystal structure of the C67A mutant of isopentenyl diphosphate isomerase complexed with a mechanism-based irreversible inhibitor.](http://www.ncbi.nlm.nih.gov/pubmed/14696183?ordinalpos=6&itool=EntrezSystem2.PEntrez.Pubmed.Pubmed_ResultsPanel.Pubmed_DefaultReportPanel.Pubmed_RVDocSum) Prot 54:216-221.

S181. Grochowski LL, Xu H, White RH (2006) *Methanocaldococcus jannaschii* uses a modified mevalonate pathway for biosynthesis of isopentenyl diphosphate. J Bacteriol 188:3192-3198.

S182. Payandeh J, Fulihashi M, Gillon W, Pai EF (2006) The crystal structure of (*S*)-3-*O*-geranylgeranylglyceryl phosphate synthase reveals an ancient fold for an ancient enzyme. J Biol Chem 281:6070-6078.

S183. Mareso AW, Schneewind O (2008) Sortase as a target of anti-infective therapy. Pharmacol Rev 60:128-141.

S184. Mareso AW, Wu R, Kern JW, Zhang R, Janik D et al (2007) Activation of inhibitors by sortase triggers irreversible modification of the active site. J Biol Chem 282:23129-23139.

S185. Prætorius-Ibba M, Ibba M (2003) Aminoacyl-tRNA synthesis in archaea: different but not unique. Mol Microbiol 48: 631-637.

S186. Kim S, Lee SW, Choi EC, Choi SY (2003) Aminoacyl-tRNA synthetases and their inhibitors as a novel family of antibiotics. Appl Microbiol Biotechnol 61:278-288.

S187. Tumbula D, Vothknecht UC, Kim HS, Ibba M, et al. Archaeal amino-tRNA synthesis: diversity replaces dogma. Genet 152:1269-1276.

S188. Tumbula DL, Becker HD, Chang WZ, Söll D (2000) Domain-specific recruitment of amide amino acids for protein synthesis. Nature 407:106-110.

S189. Sheppard K, Sherrer RL, Söll D (2008) *Methanothermobacter thermoautotrophicus* tRNA^Gln^ confines the amidotransferase GatCAB to asparaginyl-tRNA^Asn^ formation. J Mol Biol 377:845-853.

S190. Klipcan L, Frenkel-Morgenstern M, Safro MG (2008) Presence of tRNA-dependent pathways correlates with high cysteine content in methanogenic Archaea. Trends Genet 24:59-63.

S191. Sheppard K, Yuan J, Hohn MJ, Jester B, Devine KM et al. From one amino acid to another: tRNA-dependent amino acid biosynthesis. Nucleic Acids Res 36:1813-1825.

S192. Schmitt E, Panvert M, Blanquet S, Mechulam Y (2005) [Structural basis for tRNA-dependent amidotransferase function.](http://www.ncbi.nlm.nih.gov/pubmed/16216574?ordinalpos=3&itool=EntrezSystem2.PEntrez.Pubmed.Pubmed_ResultsPanel.Pubmed_DefaultReportPanel.Pubmed_RVDocSum) Struct 13:1421-1433.

S193. Oshikane H, Sheppard K, Fukai S, Nakamure Y, Ishitani R et al. [Structural basis of RNA-dependent recruitment of glutamine to the genetic code.](http://www.ncbi.nlm.nih.gov/pubmed/16809540?ordinalpos=2&itool=EntrezSystem2.PEntrez.Pubmed.Pubmed_ResultsPanel.Pubmed_DefaultReportPanel.Pubmed_RVDocSum) Science 312:1950-1954.

S194. Ataide SF, Ibba M (2006) Small molecules: big players in the evolution of protein synthesis. ACS Chem Biol 1:285-297.

S195. Jenal U, Rechsteiner T, Pan PY, Bühlmann E, Meile L et al (1991) Isoleucyl-tRNA synthetase of *Methanobacterium thermautotrophicum* Marburg. J Biol Chem 266:10570-10577.

S196. Pohlmann J, Brötz-Oesterhelt H (2004) New aminoacyl-tRNA synthetase inhibitors as antibacterial agents. Curr Drug Targets-Infect Dis 4:261-272.

S197. Ambrogelly A, Kamtekar S, Stathopoulos C, Kennedy D, Söll D (2005) Asymmetric behavior of archaeal proly-tRNA synthetase. FEBS Lett 579:6017-6022.

S198. Ahel I, Stathopoulos C, Ambrogelly A, Sauerwald A, Toogood H et al (2002) Cysteine activation is an inherent *in vitro* property of prolyl-tRNA synthetases. J Biol Chem 277:34743-34748.

S199. Ahel D, Slade D, Mocibob M, Söll D, Weygand-Durasevic I (2005) Selective inhibition of divergent seryl-tRNA synthetases by serine analogues. FEBS Lett 579:4344-4348.

S200. Kim H-S, Vothknecht UC, Hedderich R, Celic I, Söll D (1998) Sequence divergence of seryl-tRNA synthetases in Archaea. J Bacteriol 180:6446-6449.

S201. Kang YN, Tran A, White RH, Ealick SE (2007) A novel function for the N-terminal nucleophile hydrolase fold demonstrated by the structure of an archaeal inosine monophosphate cyclohydrolase. Biochem 46:5050-5062.

S202. Graupner M, Xu H, White RH (2002) New class of IMP cyclohydrolase in *Methanococcus jannaschii*. J Bacteriol 184: 1471-1473.

S203. Zhang Y, White, RH, Ealick SE (2008) Crystal structure and function of 5-formaminoimidazole-4-carboxamide ribonucleotide synthetase from *Methanocaldococcus jannaschii.* Biochem 47:205-217.

S204. Bello AM, Poduch E, Liu Y, Wei L, Crandall et al (2007) [A potent, covalent inhibitor of orotidine 5'-monophosphate decarboxylase with antimalarial activity.](http://www.ncbi.nlm.nih.gov/pubmed/17290979?ordinalpos=6&itool=EntrezSystem2.PEntrez.Pubmed.Pubmed_ResultsPanel.Pubmed_DefaultReportPanel.Pubmed_RVDocSum) J Med Chem 50:915-21.

S205. Nyce GW, White RH (1996) dTMP biosynthesis in Archaea*.* J Bacteriol 178:914-916.

S206. Sarkar N, Langley D, Paulus H (1977) Biological function of gramicidin: selective inhibition of RNA polymerase. Proc Natl Acad Sci USA 74:1478-1482.

S207. Hilpert R, Winter J, Hammes W, Kandler O (1981) The sensitivity of archaebacteria to antibiotics. Zbl Bakt Hyg I Abt Orig C2:11-20.

S208. Šurín S, Cubonová L, Majernik AI, McDermott P, Chong JP et al (2007) Isolation and characterization of an amiloride-resistant mutant of *Methanothermobacter thermoautotrophicus* possessing a defective Na^+^/H^+^ antiport. FEMS Microbiol Lett 269:301-308.

S209. Hunter S, Apweiler R, Attwood TK, Bairoch A, Bateman A et al (2009) [InterPro: the integrative protein signature database.](http://www.ncbi.nlm.nih.gov/pubmed/18940856?ordinalpos=12&itool=EntrezSystem2.PEntrez.Pubmed.Pubmed_ResultsPanel.Pubmed_DefaultReportPanel.Pubmed_RVDocSum) Nucleic Acids Res 37(Database issue):D211-215.

S210. DiMarco AA, Bobik TA, Wolfe RS (1990) Unusual coenzymes of methanogenesis. Annu Rev Biochem 59:355-394.

S211. Thauer RK, Bonacher LG (1994) Biosynthesis of coenzyme F_430_, a nickel porphinoid involved in methanogenesis. In: The biosynthesis of the tetrapyrrole pigments. Wiley, Chichester (*Ciba Foundation Symposium*) 180:210-227.

S212. Vermeij P, Pennings JLA, Maassen SM, Keltjens JT, Vogels GD (1997) Cellular levels of factor 390 and methanogenic enzymes during growth of *Methanobacterium thermautotrophicum* ΔH. J Bacteriol 179:6640-6648.

S213. Pfaltz A, Kobelt A, Hüster R, Thauer RK (1987) Biosynthesis of coenzyme F_430_ in methanogenic bacteria. Eur J Biochem 170:459-467.

S214. Schulz JO, Schubert W-D, Moser J, Jahn D, Heinz DW (2006) Evolutionary relationship between initial enzymes of tetrapyrrole biosynthesis. J Mol Biol 358:1212-1220.

S215. Moser J, Schubert W-D, Heinz DW, Jahn D (2002) Tetrapyrroles: their life, birth and death. Biochem Soc Trans 30: 579-584.

S216. Gilles H, Thauer RK (1983) Uroporphyrinogen III, an intermediate in the biosynthesis of the nickel-containing factor F_430_ in *Methanobacterium thermoautotrophicum*. Eur J Biochem135:109-112.

S217. Graham DE, White RH (2002) Elucidation of methanogenic coenzyme biosyntheses: from spectroscopy to genomics. Nat Prod Rep 19:133-147.

S218. Drevland RM, Jia Y, Palmer DRJ, Graham DE (2008) Methanogen homoaconitase catalyses both hydrolase reactions in Coenzyme B biosynthesis. J Biol Chem 283:28888-28896.

S219. White RH (2001) Biosynthesis of methanogenic cofactors. Vitam Horm 61:299-337.

S220. Howell DM, Harich K, Xu H, White RH (1998) α-Keto acid chain elongation reactions involved in the biosynthesis of Coenzyme B (7-mercaptoheptanoyl threonine phosphate). Biochem 37:10108-10117.

S221. Howell DM, Graupner M, Xu H, White RH (2000) Identification of enzymes homologous to isocitrate dehydrogenase that are involved in Coenzyme B and leucine biosynthesis in Methanoarchaea. J Bacteriol 182:5013-5016.

S222. Grochowski LL, Xu H, White RH (2009) [An iron(II) dependent formamide hydrolase catalyzes the second step in the archaeal biosynthetic pathway to riboflavin and 7,8-didemethyl-8-hydroxy-5-deazariboflavin.](http://www.ncbi.nlm.nih.gov/pubmed/19309161?ordinalpos=1&itool=EntrezSystem2.PEntrez.Pubmed.Pubmed_ResultsPanel.Pubmed_DefaultReportPanel.Pubmed_RVDocSum) Biochem 48:4181-4188.

S223. Grochowski LL, Xu H, White RH (2008) Identification and characterization of the 2-phospho-L-lactate guanyltransferase involved in coenzyme F_420_ biosynthesis. Biochem 47:3033-3037.

S224. Kengen SW, von den Hoff HW, Keltjens JT, van der Drift C, Vogels GD (1991) F_390_ synthetase and F_390_ hydrolase from *Methanobacterium thermoautotrophicum* (strain delta H). Biofact 3:61-65.

S225. Vermeij P, Detmers FJM, Broers FJM, Keltjens JT, Drift C (1994) Purification and characterization of coenzyme F_390_ synthetase from *Methanobacterium thermoautrophicum* (strain ΔH). FEBS J 226:185-191.

S226. Vermeij P, Vinke E, Keltjens JT, van der Drift C (1995) Purification and properties of coenzyme F_390_ hydrolase from *Methanobacterium thermoautotrophicum* (strain Marburg). Eur J Biochem 234:592-597.

S227. Li H, Graupner M, Xu H, White RH (2003) CofE catalyses the addition of two glutamates to F_420_-0 in F_420_ coenzyme biosynthesis in *Methanococcus jannaschii*. Biochem 42:9771-9778.

S228. Kwang-Pil C, Bair T, Bae Y-M, Daniels L (2001) Use of transposon Tn5367 mutagenesis and a nitroimidazopyran-based selection system to demonstrate a requirement for *fbiA* and *fbiC* in coenzyme F_420_ biosynthesis by *Mycobacterium bovis* BCG. J Bacteriol 183:7058-7066.

S229. Nocek B, Evdokimova E, Proudfoot M, Kudritska M, Grochowski LL et al (2007) [Structure of an amide bond forming F(420):gamma-glutamyl ligase from *Archaeoglobus fulgidus* -- a member of a new family of non-ribosomal peptide synthases.](http://www.ncbi.nlm.nih.gov/pubmed/17669425?ordinalpos=6&itool=EntrezSystem2.PEntrez.Pubmed.Pubmed_ResultsPanel.Pubmed_DefaultReportPanel.Pubmed_RVDocSum) J Mol Biol 372:456-469.

S230. Kwang-Pil C, Kendrick N, Daniels L (2002) Demonstration that *fbiC* is required for *Mycobacterium bovis* BCG for coenzyme F_420_ and FO biosynthesis. J Bacteriol 184:2420-2428.

S231. Guerra-Lopez D, Daniels L, Rawat M (2007) *Mycobacterium smegmatis* mc 155 *fbiC* and MSMEG_2392 are involved in triphenylmethane dye decolorisation and coenzyme F_420_ biosynthesis. Microbiol 153:2724-2732.

S232. Graham DE, Xu H, White RH (2003) Identification of the 7, 8-didemethyl-8-hydroxy-5-deazariboflavin synthase required for coenzyme F_420_ biosynthesis. Arch Microbiol 180:455-464.

S233. Joerger AC, Mueller-Dieckmann C, Schulz GE (2002) Structures of L-fuculose-1-phosphate aldolase mutants outlining motions during catalysis. J Mol Biol 303:531-543.

S234. Schümperli M, Pellaux R, Panke S (2007) Chemical and enzymatic routes to dihydroxyacetone phosphate. Appl Microbiol Biotechnol 75:33-45.

S235. Grochowski LL, Xu H, White RH (2006) [Identification of lactaldehyde dehydrogenase in *Methanocaldococcus jannaschii* and its involvement in production of lactate for F_420_ biosynthesis.](http://www.ncbi.nlm.nih.gov/pubmed/16585745?ordinalpos=9&itool=EntrezSystem2.PEntrez.Pubmed.Pubmed_ResultsPanel.Pubmed_DefaultReportPanel.Pubmed_RVDocSum) J Bacteriol 188:2836-2844.

S236. Nam Shin J, Kim M-J, Choi J-A, Chun KO (2007) Characterization of aldolase from *Methanococcus jannaschii* by gas chromatography. J Biochem Mol Biol 40:801-804.

S237. Forouhar F, Abashidze M, Xu H, Grochowski LL, Seetharaman J et al (2008) [Molecular insights into the biosynthesis of the F_420_ coenzyme.](http://www.ncbi.nlm.nih.gov/pubmed/18252724?ordinalpos=4&itool=EntrezSystem2.PEntrez.Pubmed.Pubmed_ResultsPanel.Pubmed_DefaultReportPanel.Pubmed_RVDocSum) J Biol Chem 283:11832-11840.

S238. Graupner M, Xu H, White RH (2002) Characterization of the 2-phospho-L-lactate transferase enzyme involved in coenzyme F_420_ biosynthesis in *Methanococcus jannaschii*. Biochem 41:3754-3761.

S239. Wise EL, Graham DE, White RH, Rayment I (2003) The structural determination of phosphosulfolactate synthase from Methanococcus jannaschii at 1.7-A resolution: an enolase that is not an enolase. J Biol Chem 278:45858-45863.

S240. Graham DE, Xu H, White RH (2002) Identification of Coenzyme M biosynthetic phosphosulfolactate synthase. J Biol Chem 277:13421-13429.

S241. Graupner M, White RH (2001) The first examples of (*S*)-2-hydroxyacid dehydrogenases catalysing the transfer of the pro-4*S* hydrogen of NADH are found in the Archaea. Biochem Biophys Acta 1548:169-173.

S242. Graupner M, Xu H, White RH (2000) Identification of an archaeal 2-hydroxy acid dehydrogenase catalysing reactions involved in coenzyme biosynthesis in methanoarchaea. J Bacteriol 182:3688-3692.

S243. Kezmarsky ND, Xu H, Graham DE, White RH (2005) [Identification and characterization of a L-tyrosine decarboxylase in *Methanocaldococcus jannaschii*.](http://www.ncbi.nlm.nih.gov/pubmed/15715981?ordinalpos=64&itool=EntrezSystem2.PEntrez.Pubmed.Pubmed_ResultsPanel.Pubmed_DefaultReportPanel.Pubmed_RVDocSum) Biochim Biophys Acta 1722:175-182.

S244. Dumitru R, Palencia H, Schroeder SD, DeMontigny BA, Takacs JM et al (2003) [Targeting methanopterin biosynthesis to inhibit methanogenesis.](http://www.ncbi.nlm.nih.gov/pubmed/14660371?ordinalpos=41&itool=EntrezSystem2.PEntrez.Pubmed.Pubmed_ResultsPanel.Pubmed_DefaultReportPanel.Pubmed_RVDocSum) Appl Environ Microbiol 69:7236-7241.

S245. Dumitru RV, Ragsdale SW (2004) [Mechanism of 4-(beta-D-ribofuranosyl)aminobenzene 5'-phosphate synthase, a key enzyme in the methanopterin biosynthetic pathway.](http://www.ncbi.nlm.nih.gov/pubmed/15262968?ordinalpos=2&itool=EntrezSystem2.PEntrez.Pubmed.Pubmed_ResultsPanel.Pubmed_DefaultReportPanel.Pubmed_RVDocSum) J Biol Chem 279:39389-39395.

S246. Scott JW, Rasche ME (2002) Purification, overproduction, and partial characterisation of RFAP synthase, a key enzyme in the methanopterin biosynthesis pathway. J Bacteriol 184:4442-4448.

S247. Chistoserdova L, Vorholt J, Thauer RK, Lidstrom ME (1998) C_1_ transfer enzymes and coenzyme linking methylotrophic bacteria and methanogenic archaea. Science 281:99-102.

S248. Rasche ME, White RH (1998) [Mechanism for the enzymatic formation of 4-(beta-D-ribofuranosyl)aminobenzene 5'-phosphate during the biosynthesis of methanopterin.](http://www.ncbi.nlm.nih.gov/pubmed/9698382?ordinalpos=146&itool=EntrezSystem2.PEntrez.Pubmed.Pubmed_ResultsPanel.Pubmed_DefaultReportPanel.Pubmed_RVDocSum) Biochem 37:11343-11351.

S249. Chistoserdova L, Jenkins C, Kalyuzhnaya MG, Marx CJ, Lapidus A et al (2004) The enigmatic Planctomyctes may hold a key to the origins of methanogenesis and methylotrophy. Mol Biol Evol 21:1234-1241.

S250. Howell DM, White RH (1997) D-erythro-neopterin biosynthesis in the methanogenic archaea *Methanococcus thermophila* and *Methanobacterium* *thermoautotrophicum* ΔH. J Bacteriol 179:5165-5170.

S251. Grochowski LL, Xu H, Leung K, White RH (2007) Characterization of an Fe^2+^-dependent archaeal-specific GTP cyclohydrolase, MptA, from *Methanocaldococcus jannaschii.* Biochem 46:6658-6667.

S252. [Schneider K](http://www.ncbi.nlm.nih.gov/sites/entrez?Db=pubmed&Cmd=Search&Term="Schneider K"%5BAuthor%5D&itool=EntrezSystem2.PEntrez.Pubmed.Pubmed_ResultsPanel.Pubmed_DiscoveryPanel.Pubmed_RVAbstractPlus), [Dimroth P](http://www.ncbi.nlm.nih.gov/sites/entrez?Db=pubmed&Cmd=Search&Term="Dimroth P"%5BAuthor%5D&itool=EntrezSystem2.PEntrez.Pubmed.Pubmed_ResultsPanel.Pubmed_DiscoveryPanel.Pubmed_RVAbstractPlus), [Bott M](http://www.ncbi.nlm.nih.gov/sites/entrez?Db=pubmed&Cmd=Search&Term="Bott M"%5BAuthor%5D&itool=EntrezSystem2.PEntrez.Pubmed.Pubmed_ResultsPanel.Pubmed_DiscoveryPanel.Pubmed_RVAbstractPlus) (2000) Identification of triphosphoribosyl-dephospho-CoA as precursor of the citrate lyase prosthetic group. FEBS Lett 483:165-168.

S253. Chistoserdova L, Che S-W, Lapudis A, Lidstrom ME (2003) Methylotrophy in *Methylobacterium extorquens* AM1 from a genomic point of view. J Bacteriol185:2980-2987.

S254. Bauer M, Lombardot T, Teeling H, Ward NL, Amann RI et al. Archaea-like genes for C_1_-transfer enzymes in *Planctomycetes*: phylogenetic implications of their unexpected presence in this phylum. J Mol Evol 59:571-586.

S255. Morrison SD, Roberts SA, Zegeer AM, Montfort WR, Bandarian V (2008) [A new use for a familiar fold: the X-ray crystal structure of GTP-bound GTP cyclohydrolase III from *Methanocaldococcus jannaschii* reveals a two metal ion catalytic mechanism.](http://www.ncbi.nlm.nih.gov/pubmed/18052207?ordinalpos=1&itool=EntrezSystem2.PEntrez.Pubmed.Pubmed_ResultsPanel.Pubmed_DefaultReportPanel.Pubmed_RVDocSum) Biochem 47:230-242.

S256. Graham DE, Xu H, White RH (2002) A member of a new class of GTP cyclohydrolases produces formylaminopyrimidine nucleotide monophosphates*.* Biochem 41:15074-15084.

S257. Ungerfeld EM, Rust SR, Boone DR, Liu Y (2004) Effects of several inhibitors on pure cultures of ruminal methanogens. J Appl Microbiol 97:520-526.

S258. Ungerfeld EM, Rust SR, Burnett R (2007) Increases in microbial nitrogen production and efficiency *in vitro* with three inhibitors of ruminal methanogenesis. Can J Microbiol 53:496-503.

S259. Nagar-Anthal KR, Worrell VE, Teal R, Nagle DP (1996) The pterin lumazine inhibits growth of methanogens and methane formation. Arch Microbiol 166:136-140.

S260. Fischer M, Schott AK, Römisch W, Ramsperger A, Augustin M et al (2004) [Evolution of vitamin B_2_ biosynthesis. A novel class of riboflavin synthase in Archaea.](http://www.ncbi.nlm.nih.gov/pubmed/15381435?ordinalpos=5&itool=EntrezSystem2.PEntrez.Pubmed.Pubmed_ResultsPanel.Pubmed_DefaultReportPanel.Pubmed_RVDocSum) J Mol Biol 343:267-78.

S261. Fischer M, Römisch W, Illarionov B, Eisenreich W, Bacher A (2005) [Structures and reaction mechanisms of riboflavin synthases of eubacterial and archaeal origin.](http://www.ncbi.nlm.nih.gov/pubmed/16042598?ordinalpos=4&itool=EntrezSystem2.PEntrez.Pubmed.Pubmed_ResultsPanel.Pubmed_DefaultReportPanel.Pubmed_RVDocSum) Biochem Soc Trans 33:780-784.

S262. Osterman A, Overbeek R (2003) Missing genes in metabolic pathways: a comparative genomics approach. Curr Opin Chem Biol 7:238-251.

S263. Römisch-Margl W, Eisenreich W, Haase I, Bacher A, Fischer M (2008) 2,5-diamino-6-ribitylamino-4(*3H*)-pyrimidinone 5′-phosphate synthases of fungi and archaea. FEBS J 275:4403-4414.

S264. Mashhadi Z, Zhang H, Xu H, White RH (2008) Identification and characterisation of an archeal-specific riboflavin kinase. J Bacteriol 190:2615-2518.

S265. Ammelburg M, Hartmann MD, Djuranovic S, Alva V, Koretke KK et al (2007) [A CTP-dependent archaeal riboflavin kinase forms a bridge in the evolution of cradle-loop barrels.](http://www.ncbi.nlm.nih.gov/pubmed/18073108?ordinalpos=1&itool=EntrezSystem2.PEntrez.Pubmed.Pubmed_ResultsPanel.Pubmed_DefaultReportPanel.Pubmed_RVDocSum) Structure 15:1577-9150.

S266. Konig H, Hartmann E, Karcher U (1994) Pathways and principles of the biosynthesis of methanobacterial cell wall polymers. Syst Appl Microbiol 16:510-517.

S267. Kandler O, Konig H (1978) Chemical composition of the peptidoglycan-free cell walls of methanogenic bacteria. Arch Microbiol 118:141-152.

S268. Perez-Bercoff A, Koch J, Burglin TR (2006) LogoBar: bar graph visualization of protein logos with gaps. Bioinformatics 22:112-114.

S269. Larkin MA, Blackshields G, Brown NP, Chenna R, McGettigan PA et al (2007) Clustal W and Clustal X version 2.0. Bioinformatics 23:2947-2948.

S270. Waterhouse AM, Proctor JB, Martin DM, Clamp M, Barton GJ (2009) Jalview Version 2—a multiple sequence alignment editor and analysis workbench. Bioinformatics 25:1189-1191.
